# Effect of Selection for Low and High *Varroa destructor* Population Growth Rates on the Honey Bee Transcriptome

**DOI:** 10.3390/pathogens14111077

**Published:** 2025-10-22

**Authors:** Alvaro De la Mora, Paul H. Goodwin, Tatiana Petukhova, Ernesto Guzman-Novoa

**Affiliations:** 1 Department of Veterinary Pathology, Western College of Veterinary Medicine, University of Saskatchewan, 52 Campus Drive, Saskatoon, SK S7N 5B4, Canada; 2 School of Environmental Sciences, University of Guelph, 50 Stone Road East, Guelph, ON N1G 2W1, Canada; pgoodwin@uoguelph.ca (P.H.G.); eguzman@uoguelph.ca (E.G.-N.); 3 Department of Population Medicine, University of Guelph, 50 Stone Road East, Guelph, ON N1G 2W1, Canada; tpetukho@uoguelph.ca

**Keywords:** *Apis mellifera*, *Varroa destructor*, selective breeding, colony collapse disorder, transcriptomics, low *Varroa* population growth

## Abstract

*Varroa destructor* is a major health problem for honey bees (*Apis mellifera*). Selective breeding of *Varroa*-resistant bees is a suitable long-term solution to *Varroa* parasitism. After three generations of selecting honey bees for lower (resistant) and higher (susceptible) *V. destructor* population growth (LVG and HVG, respectively), LVG bees showed increased behavioral, cellular, and humoral immunity against *Varroa*. To further analyze resistance, the transcriptomes of both bee genotypes were examined, revealing that parasitized LVG bees had fewer differentially expressed genes (DEGs) than parasitized HVG bees, indicating a reduced impact by *Varroa* with greater resistance. Annotations of the altered DEGs showed that both genotypes were affected with an increased demand for energy, protein, and repair during parasitism. However, there were also DEGs in LVG bees, possibly related to resistance, such as up-regulation of odorant binding protein genes and down-regulation of the corazonin receptor gene, whereas DEGs in the HVG bees may be more related to stress, such as up-regulation of ATP synthase and down-regulation of the transcription factor dorsal. Overall, this work shows that selection for LVG and HVG bees resulted in genotypes with widespread differences in gene expression during *Varroa* parasitism, which may be related to resistance and susceptibility.

## 1. Introduction

Western honey bees (*Apis mellifera*) experience increased mortality due to the parasitic mite *Varroa destructor*, resulting in major losses each year [1]. For example, a review of colony losses in Europe showed that most colonies died after three years if only typical colony management was used without *Varroa* control treatment [2]. *Varroa* punctures the bee cuticle and consumes fat body tissue and hemolymph from both brood and adults [3,4], as well as injecting a variety of proteins in its saliva that can damage hemocytes [5]. It can also inject or stimulate latent viruses like deformed wing virus (DWV) [6,7]. Both *Varroa* parasitism and DWV infections decrease the immune response and lifespan of bees [1].

Parasitism by *Varroa* can also alter the transcriptome of the honey bee. For example, parasitized adult bees had 99 up-regulated differentially expressed genes (DEGs) mostly related to immune and sphingolipid mechanisms, and 79 down-regulated DEGs mostly related to oxidative stress and olfactory recognition, resulting in the conclusion that immunity was induced but detection of the parasite and stress responses were suppressed [8]. Honey bees exposed to *Varroa* showed up-regulation of 126 DEGs and down-regulation of 46 DEGs including those related to stress, immunity, and neurological system function [9]. Another example is 43 down-regulated DEGs and 57 up-regulated DEGs found in the brains of *Varroa*-parasitized bees, some of which were related to neurodegenerative disorders and viral myocarditis, indicating that the DEGs could help to explain neurological dysfunction and viral damage in parasitized bees [10]. Thus, transcriptomic studies have been very useful to better understand the interactions of bees with *Varroa*, demonstrating a diversity of functions that are affected during parasitism.

Control of *Varroa* primarily depends upon the use of synthetic acaricides, but *Varroa* can develop resistance against them [11]. Thus, alternative control methods are needed. One alternative to control *Varroa* is to breed honey bees for resistance against the mite, such as by selecting for low *Varroa* population growth (LVG) [12,13] or selecting for specific resistant mechanisms against the mite like increased hygienic behavior [14] or grooming behavior [15,16,17]. Changes in gene expression have been associated with selection for resistance to the mite, such as *Varroa*-parasitized bees selected for high *Varroa*-sensitive hygiene (VSH) versus low VSH; up-regulated DEGS were associated with neural connections and brood care behavior, whereas down-regulated DEGs were related to visual and olfactory aspects of behavior resulting in the conclusion that some aspects of neural activity and behavior were increased while others were decreased [18]. Parasitized bees selected for lower *Varroa* infestation levels, compared to non-selected bees, showed up-regulation of a transcriptional activator gene and down-regulation of an embryonic central nervous system development gene [19]. Morfin et al. [20] found that bees selected for LVG had up-regulated DEGs related to odorant binding proteins, neuron regulation, and ecdysone synthesis compared to those bees selected for high *Varroa* population growth (HVG) when undergoing grooming behavior, indicating that selection for LVG affected parasite detection and neural functions associated with increased hygienic behavior.

The goal of this work was to examine the transcriptomes of bees described by De la Mora et al. [13], who reported that three generations of selecting colonies for LVG and HVG resulted in adult LVG honey bees with 72% less mite infestation and 46% greater longevity compared to adult HVG bees. The LVG bees also differed in many aspects associated with *Varroa* resistance, including 14–61% more grooming behavior (proportion of groomer bees and mutilated mites), 35% more hygienic behavior (percentage of cleaned cells), 55–80% more hemocytes, and 62–82% higher gene expression for hymenoptaecin and defensin 2 [21]. This indicated widespread differences following selection, and this study sought to further examine the differences between the genotypes by comparing the transcriptomes of LVG and HVG bees, with and without *Varroa* parasitism, to identify gene expression differences that may be associated with resistance or susceptibility.

## 2. Materials and Methods

### 2.1. Samples of Bees Selected for LVG and HVG That Were Treated with V. destructor

Honey bee colonies were previously selected for three generations for lower (resistant) and higher (susceptible) rates of *Varroa* population growth as per De la Mora et al. [13]. Briefly, *Varroa* populations were determined by assessing the number of mites that fell onto sticky boards per day in spring and summer. Then, the rate of *Varroa* population growth was calculated by estimating the proportional difference between the *Varroa* counts in late summer (August) and those in spring (May). Colonies with the lowest *Varroa* population growth were classified as LVG, whereas colonies with the highest *Varroa* population growth were classified as HVG. Three colonies per genotype were randomly chosen from the third generation of selection for LVG and HVG [13]. Combs from each colony were incubated (Lab-Line Instruments, Inc., Dubuque, IA, USA) at 35 °C and 60% RH overnight in screened emerging cages (50.3 × 7.3 × 25.2 cm), and newly emerged bees were collected [10]. *Varroa* from highly infested colonies that were unrelated to the project were collected from adult bees using CO_2_ as per Dietemann et al. [22]. To expose bees to *Varroa*, ten newly emerged bees from each colony were placed in a wooden three-hole cage (75 × 25 × 16 mm), and to ensure that individual bees were challenged with *Varroa* parasitism, one mite was placed on each bee using a fine paintbrush. There were five cages per treatment with four treatments: LVG bees without *Varroa* (LVG-C), LVG bees with *Varroa* (LVG-V), HVG bees without *Varroa* (HVG-C), and HVG bees with *Varroa* (HVG-V). To allow *Varroa* to sufficiently parasitize the bees, the cages were incubated for 8 days at 32–35 °C and 60% RH with queen candy (sugar syrup mixed with icing sugar) and water provided. Dead bees were removed daily. Live bees were collected and stored at −80 °C.

### 2.2. RNA Extraction, Sequencing, and Library Preparation

To extract RNA from entire bees, 15 frozen bees per sample were macerated with 5 mL of One Step RNA Reagent (BioBasic, Markham, ON, Canada) as per Morfin et al. [20] with modifications as per De la Mora et al. [13], following the manufacturer’s instructions. Fifteen bees per biological replicate were used as this was found to be the fewest bees to provide low variability in gene expression analysis among worker bees from the same hive, which are half-sisters with potentially different drones as fathers [23]. The macerate was transferred, incubated at 20–22 °C for 5 min, and 300 μL of chloroform (MilliporeSigma, Burlington, MA, USA) added. After vortexing (Fisher Scientific, Waltham, MA, USA) for 15 s at 7000 rpm, the sample was incubated for 2–3 min at 20–22 °C, and then centrifuged (Symphony 417R, VWR, Mississauga, ON, Canada) at 12,000× *g* for 15 min at 4 °C. The aqueous phase was transferred, and then 500 μL 99% isopropanol was added followed by incubation for 10 min at 20–22 °C. After centrifugation at 4 °C for 10 min at 12,000× *g*, the RNA pellet was washed three times with 70% ethanol and dried for 10 min at 20–22 °C. The pellet was dissolved in 30 μL of Invitrogen UltraPure H_2_O (Fisher Scientific, Waltham, MA, USA) and sent to Génome Québec Innovation Centre, Montreal, QC, Canada, for high-throughput sequencing using an Illumina NovaSeq (25 M reads per lane) (Illumina, San Diego, CA, USA) with 100 bp paired-end reads. The library type was polyA-enriched RNA.

### 2.3. Bioinformatic Analyses

Bioinformatic analyses were performed at the Bioinformatics Core Facility, Montreal Clinical Research Institute, Montreal, QC, Canada. The quality of the raw reads was assessed with FASTQC v0.11.8 [24], and the results summarized using MultiQC, v1.14 [24]. Raw counts were calculated with FeatureCounts v1.6.0, based on the honey bee reference genome (Amel_HAv3.1) [25]. Then, trimming was performed with TRIMMOMATIC v0.36 [26]. The reads were aligned to the *Apis mellifera* reference genome (https://www.ncbi.nlm.nih.gov/datasets/genome/GCF_003254395.2/; accessed on 23 March 2024) with STAR v2.7.6a [27] using default parameters, which allows up to 30% mismatches and tolerates gaps in read sequences. Aligned RNA-seq normalized fragment counts were assembled into transcripts, and their abundance in fragments per kilobase of exon per million fragments mapped (FPKM) was determined through Cufflinks [28]. Reads per kilobase of transcript per million mapped reads (RPKM) were also calculated with the percent relative error (PRE) of the RPKM values determined for each sample. Differential Expression Analysis (DEA) was performed to determine differentially expressed genes (DEGs) with the DESeq2 R package [29] and edge R Bioconductor package v3.19 [30] based on the raw read counts. DEG heatmaps were drawn based on the z-score of the normalized count. Functional enrichment analysis of DEGs based on gene ontology (GO) and the Kyoto Encyclopedia of Genes and Genomes (KEGG; https://www.genome.jp/kegg/, accessed on 24 May 2024) pathway enrichment were performed with the gprofiler2 R package v0.2.3 (accessed on 23 March 2024) [31].

Venn diagrams were created for the DEGs using the Bioinformatics and Evolutionary Genomics website (http://bioinformatics.psb.ugent.be/webtools/Venn/, accessed on 24 May 2024) [32]. GO and KEGG pathway enrichment analyses were performed by using the g:profiler search for biological process terms, considering a depth of two hierarchical levels [31]. ShinyGO v0.741 search (https://bioinformatics.sdstate.edu/go74/; accessed on 24 May 2024) [33] was used for GO and KEGG analysis. GO analysis results (biological process, cellular component, and molecular function) were related to physiological function using the Mouse Genome Database (MGD) (https://www.informatics.jax.org/vocab/gene_ontology; accessed on 24 May 2024) [34].

### 2.4. Statistical Analyses

Transcript expression levels and tests for significant differences (*p* < 0.05) were calculated with Cuffdiff [28]. The log2 fold change (log2FC) estimated the fold change between the treatments, based on the distribution of the reads. Lists of up- and down-regulated DEGs were determined using thresholds for the adjusted *p*-value (padj) and log2FC. For graphical representations, the first threshold yielding less than 1000 DEGs in each comparison was used to generate both heatmaps and volcano plots. Statistical analyses were performed with the R 4.1.1. software (R Development Core Team, Auckland, The Netherlands) [35]. Principal component analysis (PCA) was generated with FactorMineR and was based on the regularized log-transformed normalized counts. The Shapiro–Wilk test was used to analyze normality for the number of reads, number of aligned reads, percentage of aligned reads, and percentage of GC content. The Student *t*-test was used for pairwise comparisons between genotypes and between treatments within genotypes. The chi-square test was used to make pairwise comparisons of DEGs between treatments within genotypes (HVG-V versus HVG-C and LVG-V versus LVG-C) and between genotypes regardless of treatment (LVG versus HVG).

## 3. Results

### 3.1. Treatments and Reads

**Figure 1:**
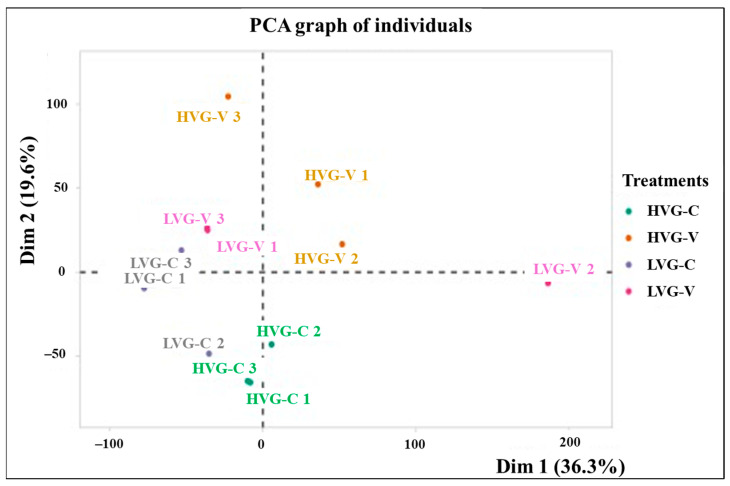
Principal component analysis (PCA) of the transcriptome of colonies 1, 2 and 3 for each treatment (LVG-C, HVG-C, LVG-V, and HVG-V). The proportion of variance is represented with the numbers in parentheses. Dim 1 and Dim 2 represent the top two dimensions of the genes among treatments (36.3% and 19.6%, respectively).

A total of 939,413,112 reads were obtained with an average of 78.3 million reads per sample (Table S1). The average percent alignment to the reference genome of *A. mellifera* was 59%, with 78.1% (±5.50) for HVG-C and HVG-V bees, which was significantly higher than that of LVG-C and LVG-V bees at 39.2% (±2.70) (t = 6.344, *p* < 0.05, df = 11). The average GC contents of the reads were 42.5% (±0.62) for HVG-C and HVG-V, and 42.3% (±0.54) for LVG-C and LVG-V bees, which were not significantly different from each other (t = −0.102, *p* > 0.05). After mapping the reads to the *A. mellifera* reference genome (Amel_HAv3.1), the gene counts between samples clustered into four groups based on principal component analysis (PCA), corresponding to the genotype and treatment, except for LVG-V replicate 2, which differed from all other samples (Figure 1).

### 3.2. DEGs from Treatment Comparisons

There was a total of 1332 DEGs based on a significant change of log2 fold or more among the treatment/genotype comparisons (Table S2). There were significantly more up-regulated DEGs per sample for the HVG than the LVG genotype (χ^2^ = 4.758, *p* < 0.05) and significantly more down-regulated DEGs per sample for the HVG than the LVG genotype (χ^2^ = 1.571, *p* < 0.05), indicating that the total effect on the transcriptome due to *Varroa* parasitism was greater with the HVG than the LVG genotype. Volcano plots showed an approximately even distribution of the fold changes for up- and down-regulated DEGs in each comparison (Figure S1).

**Figure 2:**
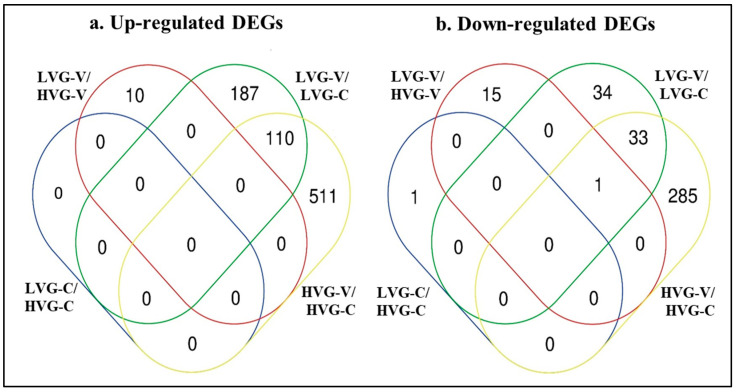
Venn diagrams showing number of DEGs only found or shared with treatment comparisons of LVG-C versus HVG-C, LVG-V versus HVG-V, LVG-V versus LVG-C, and HVG-V versus HVG-C, based on Differential Expression Analysis (DEA). (**a**) Up-regulated DEGs. (**b**) Down-regulated DEGs. The ovals marked with blue, red, green and yellow lines contain the number of DEGs in the LVG-C versus HVG-C, LVG-V versus HVG-V, LVG-V versus LVG-C, and HVG-V versus HVG-C comparisons, respectively.

The fewest total DEGs were found for the comparison between the non-parasitized bees of the two genotypes with no up-regulated genes and one down-regulated gene for LVG-C versus HVG-C (Table S2; Figure 2). That DEG was not shared with any other comparison. The next lowest was found for the comparison between the parasitized bees of the two genotypes with 10 and 16 up-regulated and down-regulated DEGs, respectively, for LVG-V versus HVG-V. Those up-regulated DEGs were unique to that comparison, while 15 of the down-regulated DEGs were unique and 1 was shared with the HVG-V versus HVG-C comparison.

In contrast, the most DEGs were found with the comparison of *Varroa*-parasitized to non-parasitized HVG bees (HVG-V versus HVG-C) at 621 and 319 up-regulated and down-regulated DEGs, respectively (Table S2; Figure 2). Among those up-regulated DEGs, 511 were unique to the comparison, while 110 were shared with the *Varroa*-parasitized to non-parasitized LVG bees (LVG-V versus LVG-C) comparison. For those down-regulated DEGs, 285 were unique to the comparison, while 33 were shared with the LVG-V versus LVG-C comparison, and 1 was shared with the non-parasitized LVG bees and non-parasitized HVG bees (LVG-C versus HVG-C) comparison. The number of DEGs comparing *Varroa*-parasitized to non-parasitized LVG bees (LVG-V versus LVG-C) was less with 297 and 68 up-regulated and down-regulated DEGs, respectively. Among those up-regulated DEGs, 187 were unique to the comparison, while 110 were shared with the *Varroa*-parasitized to non-parasitized HVG bees (HVG-V versus HVG-C) comparison. For those down-regulated DEGs, 34 were unique to the comparison, while 33 were shared with the *Varroa*-parasitized to non-parasitized HVG bees (HVG-V versus HVG-C) comparison and 1 shared with the non-parasitized LVG bees and non-parasitized HVG bees (LVG-C versus HVG-C) comparison. The differences between those comparisons were all significant (χ^2^ = 43.71, *p* < 0.05). Thus, the effect on the number of DEGs was more due to *Varroa* parasitism than genotype, and although some of the effects on gene expression due to *Varroa* parasitism were shared between genotypes, a large amount was unique to each genotype.

### 3.3. Up-Regulated DEGs

**Figure 3:**
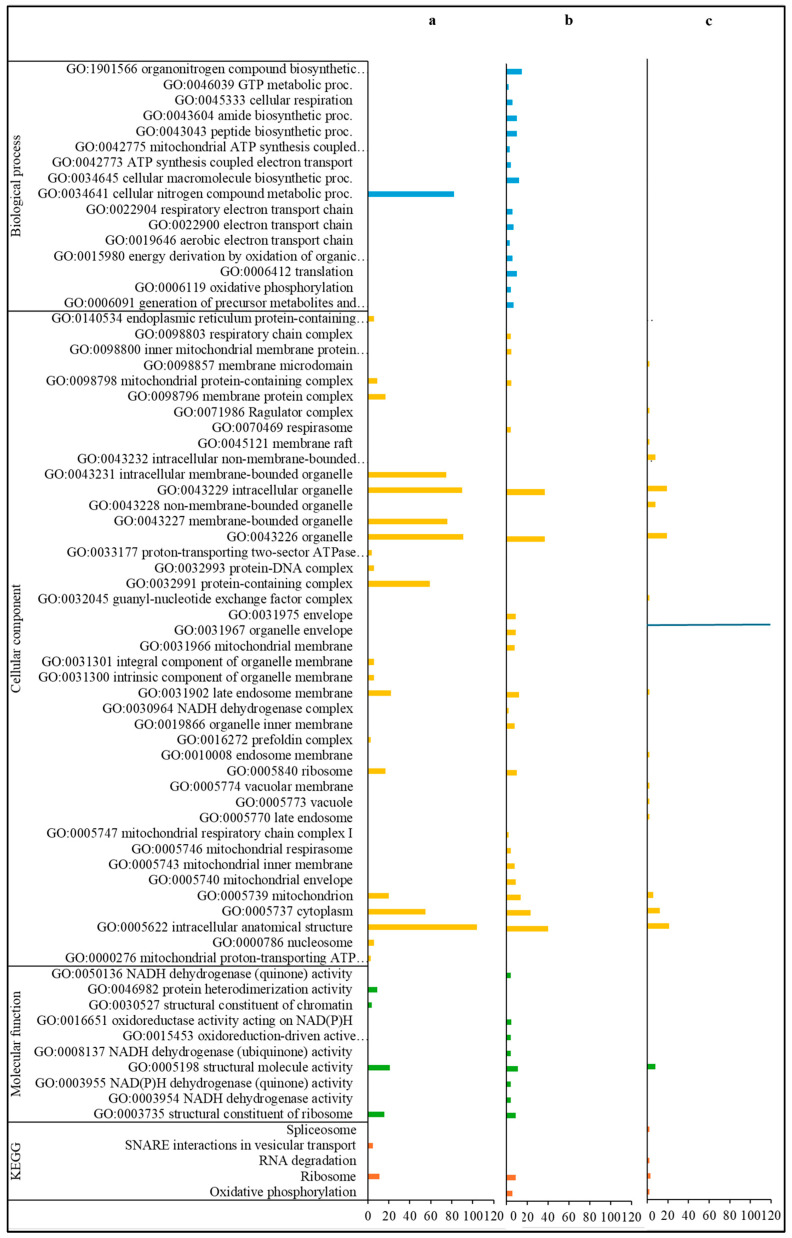
Gene ontology classification and KEGG pathways of up-regulated DEGs. DEGs only detected in the HVG-V versus HVG-C comparison (*p* < 0.05) (**a**), DEGs only found in the LVG-V versus LVG-C comparison (*p* < 0.05) (**b**), and DEGs shared between LVG-V versus LVG-C and HVG-V versus HVG-C comparisons (*p* < 0.05) (**c**). The y-axis indicates the GO (biological process, cellular component, and molecular function) and KEGG classification. The x-axis indicates the number of DEGs matching the classification. Bars in blue, yellow, green and orange colors indicate the number of DEGs for GO biological processes, GO cellular components, GO molecular functions, and KEGG pathways, respectively.

Among the annotations of the 511 up-regulated DEGs only observed in the comparison of *Varroa*-treated versus control bees of the HVG genotype (HVG-V versus HVG-C), the most common coded for ribosomal proteins (16 DEGs), ATP synthase (7 DEGs), histone (7 DEGs), and vesicle transport (4 DEGs) (Table S3). For those DEGs with GO biological processes, all were related to nitrogen metabolism, and the GO cellular components were mostly related to intracellular organelles, organelles, and membranes (Figure 3a). The GO molecular functions were mostly related to structural activity and the ribosome, and the KEGG pathways were all related to the ribosome and vesicle transport.

For the annotations of the 187 up-regulated DEGs only found in the comparison of parasitized versus non-parasitized LVG bees (LVG-V versus LVG-C), the most common were ribosomal proteins (15 DEGs), NADH dehydrogenase (7 DEGs), mitochondrion-related (7 DEGs), cytochrome (5 DEGs), and spliceosomal RNA (5 DEGs) (Table S4). For GO biological processes, most were related to the generation of energy and metabolites, biosynthesis of macromolecules and organonitrogen compounds, and nitrogen compound metabolism (Figure 3b). The GO cellular components were mostly related to intracellular structures and organelles, particularly the mitochondrion, and the GO molecular functions were mostly related to oxidoreductase activity. The KEGG pathways were mostly related to intracellular structures and the generation of energy and metabolites.

Among the 110 up-regulated DEGs shared between comparisons of the two genotypes for *Varroa* versus control bees (LVG-V versus LVG-C shared with HVG-V versus HVG-C), the most common annotations were 4 DEGs for ribosomal proteins, 4 for mitochondrion related, 3 for histone, and 3 for snRNA (Table S5). None of the DEGs were characterized by a GO biological process, but for those linked to a GO cellular component, most were related to organelles and intracellular structures (Figure 3c). The most common GO molecular function was related to the activity of structural molecules. The KEGG pathways were widely distributed without any one being more dominant.

Among the 10 up-regulated DEGs only detected in the comparison of *Varroa*-treated bees between the two genotypes (LVG-V versus HVG-V), two had closely related annotations, both of which were odorant binding proteins (Table S6). Based on the GO biological process, three DEGs were related to the regulation of molecular function, organic substance metabolism, localization, and response to other organisms (Figure S2). The GO cellular component of one DEG was related to the extracellular region. No DEGs were associated with a GO molecular function. The KEGG pathway of one DEG was related to organic substance metabolism and intracellular structure.

### 3.4. Down-Regulated DEGs

**Figure 4:**
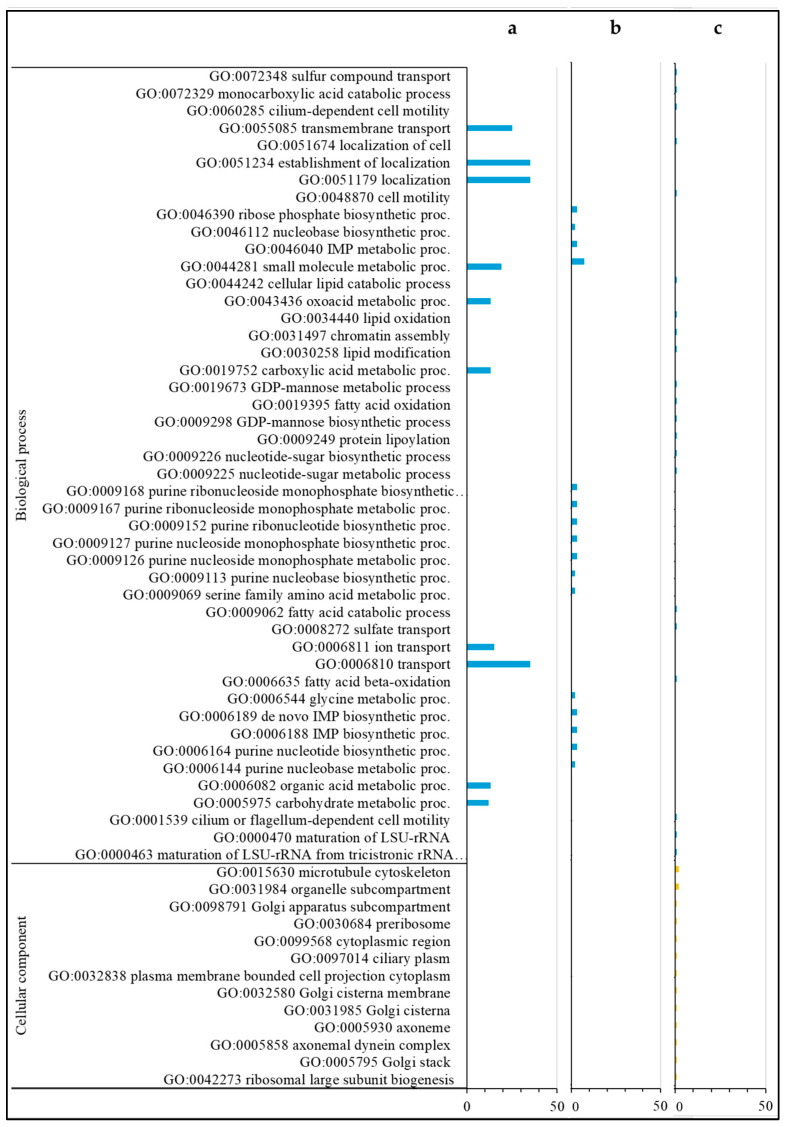
Gene ontology (biological processes and cellular components) classification of down-regulated DEGs. DEGs only found in the HVG-V versus HVG-C comparison (*p* < 0.05) (**a**), DEGs only found in the LVG-V versus LVG-C comparison (*p* < 0.05) (**b**), and DEGs shared between LVG-V versus LVG-C and HVG-V versus HVG-C comparisons (*p* < 0.05) (**c**). The y-axis indicates the GO (biological process, cellular component, and molecular function) and KEGG results. The x-axis indicates the number of genes matching the classification. Bars in blue, yellow, green and orange colors indicate the number of DEGs for GO biological processes, GO cellular components, GO molecular functions, and KEGG pathways, respectively.

**Figure 5:**
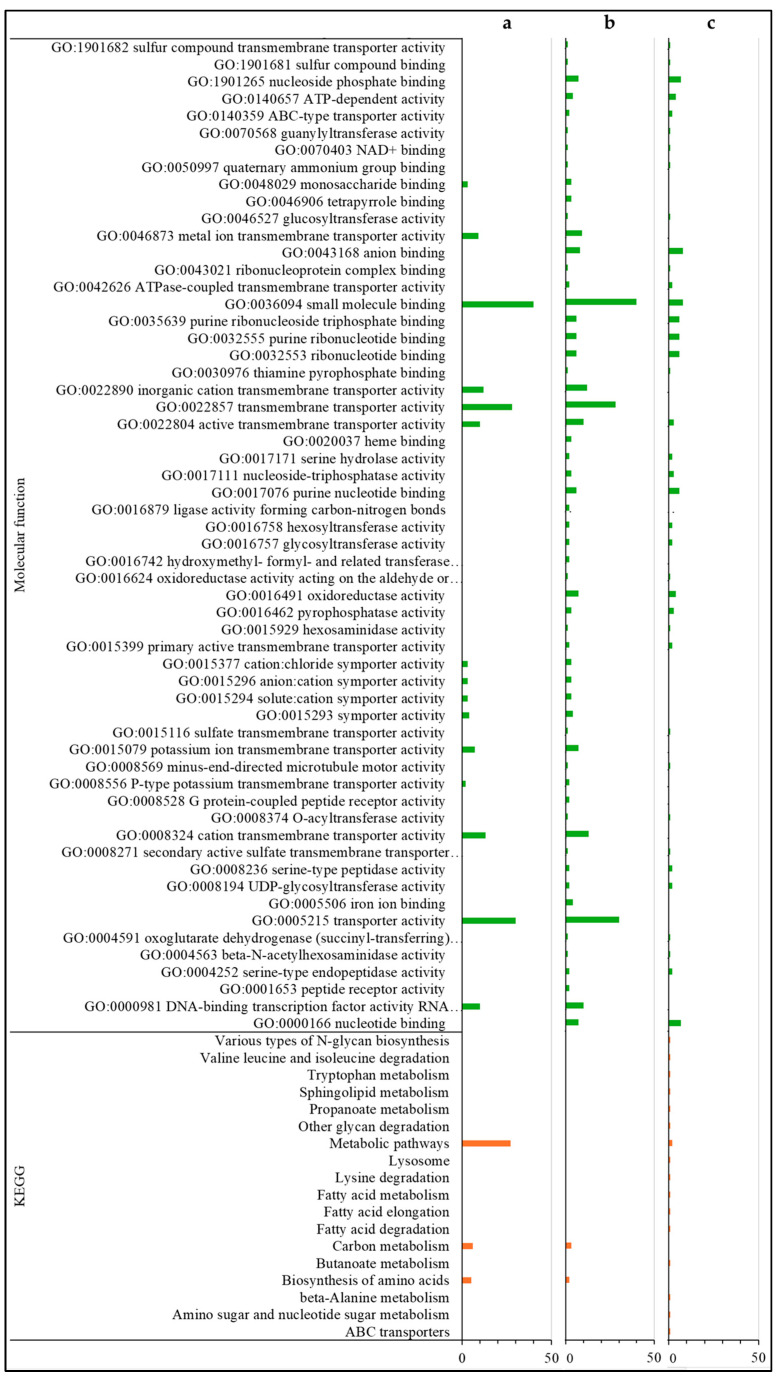
Gene ontology (molecular function) classification and KEGG pathways of down-regulated DEGs. DEGs only found in the HVG-V versus HVG-C comparison (*p* < 0.05) (**a**), DEGs only found in the LVG-V versus LVG-C comparison (*p* < 0.05) (**b**), and DEGs shared between LVG-V versus LVG-C and HVG-V versus HVG-C comparisons (*p* < 0.05) (**c**). The y-axis indicates the GO (biological process, cellular component, and molecular function) and KEGG results. The x-axis indicates the number of genes matching the classification. Bars in blue, yellow, green and orange colors indicate the number of DEGs for GO biological processes, GO cellular components, GO molecular functions, and KEGG pathways, respectively.

For the annotations of the 285 down-regulated DEGs only found in the comparison of *Varroa* to control in HVG bees (HVG-V versus HVG-C), the most common were related to serine/threonine kinase (7 DEGs), homeobox protein (6 DEGs), odorant binding and receptor (5 DEGs), acetyl-coenzyme A (4 DEGs), facilitating trehalose transporter (4 DEGs), and zinc finger protein and transporter (4 DEGs) (Table S7). For DEGs with biological GO processes, all were related to localization, organic acid, carbohydrate and small molecule metabolism, transmembrane transport, and oxidoreductase activity (Figure 4a and Figure 5a). There were no DEGs with a GO cellular component, but the DEGs with a GO molecular function were all related to transporter/transmembrane transporter activity, and small molecule, DNA, and carbohydrate binding (Figure 5a). The DEGs with KEGG pathways were all related to precursor and carbon metabolism, energy, and amino acid synthesis (Figure 5a).

The most common annotations of the 34 down-regulated DEGs only detected in the comparison of *Varroa* versus control in LVG bees (LVG-V versus LVG-C) were related to the ATP binding cassette (2 DEGs) (Table S8). For the DEGs with a GO biological process, most were related to the metabolism of carbohydrates, cellular lipid, protein, lipid/fatty acid metabolism, and cellular mobility (Figure 4b). The GO cellular components were mostly related to the Golgi apparatus and membrane organelles (Figure 4b), and the GO molecular functions were mostly related to nucleotide binding, ion binding, hydrolase activity, ATP-dependent activity, and transferase activity (Figure 5b). KEGG pathway classifications were mostly related to amino acid, fatty acid, and lipid metabolism (Figure 5b).

For the annotations of the 33 down-regulated DEGs shared between the comparison of *Varroa* versus control of both LVG and HVG bees (LVG-V versus LVG-C shared with HVG-V versus HVG-C), the most common were major royal jelly protein (4 DEGs) and receptor related (3 DEGs) (Table S9). For GO biological processes, almost all were related to nucleobase compound metabolism, oxidoreductase activity, and small molecule metabolism for energy, protein metabolism, and oxidoreductase activity (Figure 4c). The DEGs with GO cellular components were mostly related to the Golgi and cytoskeleton (Figure 4c), and GO molecular functions related to oxidoreductase activity, ion and cyclic organic compound binding, and peptide receptors (Figure 5c). KEGG pathway classifications were mostly for amino acid biosynthesis and carbon metabolism (Figure 5c).

There were no annotations of the 15 down-regulated DEGs only found in the comparison of *Varroa* treated bees between genotypes (LVG-V versus HVG-V) (Table S10). Only one DEG could be characterized by GO and KEGG with the extracellular region as a GO cellular component, and carbohydrate derivative binding as a GO molecular function. The RNA catabolic process was the KEGG pathway.

There were also no annotations for the one down-regulated DEG only detected in the *Varroa* non-treated bees between genotypes (LVG-V versus HVG-V) (Table S11). It could not be characterized by GO or KEGG analysis.

The annotation for the one down-regulated DEG shared between the three-way comparison of *Varroa* between genotypes, *Varroa* versus control of both LVG and HVG bees (LVG-V versus HVG-V shared with LVG-V versus LVG-C and shared with HVG-V versus HVG-C) denoted major royal jelly protein (Table S12). The GO molecular function was related to DNA binding, and the GO cellular component was extracellular. The KEGG pathway was RNA binding.

## 4. Discussion

The percent alignment to the reference genome of *A. mellifera* was much less for LVG than HVG bees, indicating that selection for LVG bees resulted in more sequence variants from the reference genome than HVG bees. Such differences also implied that gene expression would differ between LVG and HVG bees. The largest number of DEGs was found between non-parasitized and parasitized bees in each genotype, suggesting that changes in gene expression are mainly triggered by *V. destructor*. Shared DEGs between the *Varroa*-triggered DEGs in LVG and HVG bees indicate that there were some gene expression changes during parasitism that were not greatly affected by selection. However, more DEGs triggered by *Varroa* were unique to each genotype, indicating that not only the number of DEGs but their functions were affected by selection.

There were approximately 2.5-times more DEGs due to *Varroa* parasitism in HVG than LVG bees, even though the samples had the same number of bees and similar biomasses. This indicates that *Varroa* has a broader impact on the susceptible genotype. This may be due to greater damage and stress in the susceptible than the resistant genotype or perhaps the induction of more, but possibly less effective, resistance mechanisms in the susceptible genotype. However, another possibility is that there were fewer DEGs with the resistant genotype because selection for LVG may have reduced gene expression noise (random variation in gene expression among cells, even in the same environment) [36]. Increased expression noise can reduce the fitness of a cell by at least 25%, and this reduction cannot be substantially attenuated by gene overexpression. Also, expression noise may result in organisms being both less adapted and less adaptable [37]. Thus, in addition for selecting for *Varroa* resistance, selection of LVG bees may have resulted in less expression noise and therefore better adaptability to *Varroa* parasitism.

Up-regulated DEGs in *Varroa*-parasitized LVG bees in comparison to *Varroa*-parasitized HVG bees were those genes that may be related to resistance as they were significantly more expressed in the parasitized resistant genotype than the susceptible genotype (up-regulated DEGs only observed in the LVG-V to HVG-V comparison; Table S6). They included two odorant binding protein genes that may be related to the greater ability of LVG bees to detect odors. Volatile odors from *Varroa*-infested pupae can be learned and discriminated from non-infested pupae [38], and higher expression of odorant binding proteins was associated with bees triggering more rapid self-grooming instances [20]. Thus, higher expression of odorant binding proteins in LVG-V than HVG-V bees could indicate enhanced hygienic and grooming behaviors, as reported in bees with enhanced hygienic behavior [38]. KEGG analysis of the DEGs indicated some were related to lysozyme and glycosaminoglycan degradation. Greater lysozyme could increase *Varroa* resistance as insect lysozymes have antibacterial and antifungal activity [39]. Higher expression of the lysozyme genes was observed in *Varroa*-parasitized worker bees, which could have occurred as part of the response of the innate immune system [40]. Greater glycosaminoglycan degradation in LVG bees could increase resistance as glycosaminoglycans can promote pathogenesis by facilitating pathogen attachment, invasion, or evasion of host defense mechanisms [41]. As *Varroa* can suppress bee immunity [1], more glycosaminoglycan degradation in the LVG genotype could restrain opportunistic pathogens, such as DWV, which increases in *Varroa*-parasitized bees [6,7]; this may help explain why DWV infection levels were reduced in parasitized LVG bees compared to parasitized HVG bees [13,21].

Among the up-regulated DEGs due to the effects of *Varroa* in comparison to the control bees that were only found with LVG bees, some could be related to greater *Varroa* resistance mechanisms (up-regulated DEGs only observed in the LVG-V to LVG-C comparison; Table S4). The relatively large number of ribosomal genes among these DEGs indicates greater protein production; this is consistent with up-regulated DEGs for the spliceosome, which removes introns from a transcribed pre-mRNA [42]. More protein production is also consistent with GO analysis showing biosynthesis of macromolecules and organonitrogen compounds, and nitrogen compound metabolism. There was also up-regulation of DEGs for mitochondria indicating greater energy production, including NADH dehydrogenase, the first enzyme in the respiratory chain. This is consistent with GO and KEGG analyses showing up-regulated DEGs related to oxidoreductase activity and energy generation. Perhaps more protein- and energy-requiring processes in LVG bees during *Varroa* parasitism enable the bees to compensate for *Varroa* damage. NADH levels decrease in *Varroa*-parasitized bees due to lower energy production as the bees become weaker [43]. More protein- and energy-requiring processes may also provide energy and compounds needed for resistance mechanisms. Another more up-regulated DEG unique to parasitized LVG bees was related to cytochrome-related function, indicating that LVG bees may perform more detoxification than HVG bees during parasitism.

The majority of up-regulated DEGs were related to the effects of *Varroa* in comparison to control bees that were only found with HVG bees (up-regulated DEGs only observed in the HVG-V to HVG-C comparison; Table S3). Many were possibly related to greater stress responses or less successful resistance in the susceptible genotype. Like the LVG genotype, there were many DEGs for protein production, such as ribosomal proteins, which is consistent with GO analysis detecting DEGs related to nitrogen metabolism. Also, similar to parasitized LVG bees, there were up-regulated DEGs in HVG bees for energy, such as ATP synthase. In addition to possibly less successful resistance and greater stress, perhaps these changes are part of the compensation for greater nutrient loss due to *Varroa* feeding of the susceptible genotype. Up-regulation of DEGs for SNARE interactions could be related to the susceptibility of HVG bees as higher expression of SNARE genes is associated with a potentially higher risk of pathogen exposure, such as bacterial infection of *Drosophila melanogaster* [44].

Up-regulated DEGs triggered by *Varroa* in both LVG and HVG bees are likely due to the effects of *Varroa* that were not significantly altered from the parental material during the three rounds of selection (up-regulated DEGs shared between LVG-V to LVG-C comparison and HVG-V to HVG-C comparison; Table S5). Like the up-regulated DEGs due to *Varroa* observed only in each genotype, these DEGs included those for protein production such as ribosomal proteins, spliceosomal RNA, and energy generation, such as mitochondrion-related genes. This indicates that some increased protein and energy production occurs with parasitism similarly in both genotypes. However, unlike the DEGs triggered by *Varroa* in only the LVG or HVG bees, many of the GO terms for these DEGs shared between the genotypes were related to membranes, indicating that membrane damage may be occurring during *Varroa* feeding, regardless of resistance. One reason for bee membrane damage could be the activity of proteins in *Varroa* saliva that can lyse hemocytes, presumably due to membrane disruption [5], as well as lysis of fat body cells during their digestion by *Varroa* [3]. One up-regulated DEG in both genotypes that may have a direct relationship with susceptibility to *Varroa* is for guanyl-nucleotide exchange factor, which could result in increased exchange of GDP to GTP and activate signaling GTPases. Dysregulation of Rho GTPase function corresponds to certain pathologies, such as cancer progression and mental disabilities in humans [45]. Also, Rab GTPases are key regulators of vesicular transport that can be altered by infectious diseases as part of host defenses or as a way for pathogens to avoid host defenses [46]. Thus, changes in GTPase in both genotypes during parasitism could contribute to reduced functioning of key elements of bee metabolism and allowing *Varroa* to avoid certain defense responses [46].

Down-regulated DEGs in *Varroa*-parasitized LVG in comparison to *Varroa*-parasitized HVG bees were those genes that may be less suppressed by *Varroa* in the parasitized resistant genotype than the parasitized susceptible genotype (down-regulated DEGs only observed in the LVG-V to HVG-V comparison; Table S10). They had diverse functions with many annotated as uncharacterized protein genes, indicating that there may be resistance mechanisms not yet discovered. Only extracellular chitin binding and cell wall integrity were identified by GO and KEGG analyses. *Varroa* punctures the exoskeleton of bees, but insect cuticles have regeneration and repair mechanisms [47]. Chitin binding proteins are involved in cuticle formation [48] and are highly expressed in the integument (single layer of epidermal cells) of honey bees during their development [49]. Therefore, one might expect the up-regulation of such genes to increase damage repair during parasitism. Also, invertebrate chitin-binding proteins can have antimicrobial activity [50] and, thus, might also be expected to be increased with resistance. As those genes were suppressed instead, it is possible that the response of the more resistant bees to *Varroa* parasitism requires less repair to the cuticle or less antimicrobial activity.

Among the down-regulated DEGs due to the effects of *Varroa* in comparison to the control bees that were found only with LVG bees, some could be related to the lower impact of *Varroa* parasitism in the resistant genotype (down-regulated DEGs only observed in the LVG-V to LVG-C comparison; Table S8). GO and KEGG analyses showed that many were related to the metabolism of carbohydrates, proteins, lipids and fatty acids, such as hydrolase, and ATP-dependent and transferase activities. This indicates a suppression of general metabolism by *Varroa* in the resistant genotype, perhaps as there is a shift to resistance mechanisms. One down-regulated DEG coded for chitooligosaccharidolytic β-N-acetylglucosamindase involved in chitin degradation [51]. While it is important in molting, less activity may allow for greater retention of the cuticle when damaged by *Varroa*. Down-regulation of a corazonin receptor DEG could result in less detection of the neuropeptide hormone corazonin in the hemolymph, which is involved in insect development [52]. The corazonin receptor is expressed in salivary glands and adipocytes of the liver-like fat body, and reduced expression of its receptor increases fruit fly resistance to starvation, desiccation, and oxidative stress and affects gene expression in the fat body [53]. In honey bees, corazonin gene expression is increased with dietary stress [54]. Considering that *Varroa* removes nutrients and water from the bee when feeding, lower expression of the corazonin receptor gene in parasitized LVG bees could be an indicator that they are experiencing less nutrient stress than in parasitized HVG bees.

The majority of down-regulated DEGs were related to the effects of *Varroa* in comparison to controls that were only observed with HVG bees, and many were possibly related to higher stress impact by *Varroa* in the susceptible genotype (down-regulated DEGs only observed in the HVG-V to HVG-C comparison; Table S7). Many had GO and KEGG classifications related to carbon metabolism, indicating reduced production of various carbohydrates and intermediates in carbon metabolism, as well as amino acids. This may also be linked to down-regulated DEGs for various transporter and membrane proteins. Also, these DEGs included annotations with many regulatory elements, such as the transcription factor dorsal [55], transcription initiation factor IIB that helps to recruit RNA polymerase II into the initiation complex [56], upstream activation factor subunit spp27 [57], and poly(U)-specific endoribonuclease, which is a promoter of translation of mRNA [58]. Down-regulation of such regulatory genes could result in broad scale changes in HVG bees during parasitism, perhaps reflecting a strong suppression of many honey bee cell functions during *Varroa* parasitism of the susceptible genotype.

Down-regulated DEGs triggered by *Varroa* in both the LVG and HVG genotypes reflect similar down-regulated functions due to parasitism that were not differentially selected during selection (down-regulated DEGs shared between LVG-V to HVG-V comparison, LVG-V to LVG-C comparison, and HVG-V to HVG-C comparison; Table S9). Among these, there were DEGs for four types of major royal jelly protein. Major royal jelly protein is the main component of brood food for developing larvae [59], and its functions include neural activity related to honey bee development and division of labor [60], as well as affecting antioxidant regulation in the bodies of worker bees [61]. Thus, lower expression of these genes would result in a variety of negative impacts, such as poorly developed larvae, reduced brain function, and higher oxidative stress, which may have occurred both in parasitized LVG and HVG bees. There were also many down-regulated DEGs related to energy, protein metabolism, and nucleobase metabolism, indicating reduced production of key metabolites in parasitized bees of both genotypes.

This study has revealed that changes in gene expression occur differently following parasitism of LVG and HVG bees. Thus, selection has clearly altered the response of the bees. Many of the DEGs identified in this study may contribute directly to resistance to *Varroa* or indirectly to resistance to the stresses created during parasitism. Other genes may contribute to the susceptibility of bees to the mite. However, this does not establish a functional role for these genes in the response of either LVG or HVG bees to *Varroa*. Future research can examine the function of these genes in the response of LVG and HVG bees to *Varroa*, such as by disrupting their function using CRISPR/Cas9 mediated knockout [62].

## 5. Conclusions

Overall, this study demonstrated that a wide range of changes occurred in gene expression with *Varroa* parasitism of both resistant and susceptible bees, but based on the number of DEGs, the impacts were less broad in LVG than in HVG bees. Perhaps, one element of *Varroa* resistance is the ability of LVG bees to better limit the stresses caused by parasitism, viral replication, and immunosuppression. There are many DEGs triggered by *Varroa* in both genotypes, likely due to stresses caused by the parasite regardless of resistance. However, there were more DEGs up- or down-regulated only with either the LVG or HVG genotype, showing that selection affected bee gene expression. Some of these DEGs, such as those with higher expression in LVG for odorant binding protein, were also observed in the brains of non-parasitized LVG bees undergoing grooming behavior [20], possibly contributing to behavioral resistance to *Varroa*. Others DEGs like those for glucuronosyltransferase activity and cytochrome P450 that were up-regulated in LVG bees were also reported by Le Conte et al. [18] and may possibly contribute to humoral resistance, detoxification, and stress resistance to *Varroa.* The transcriptome of LVG bees provides new insights into the diversity of mechanisms by which these bees respond differently than HVG bees to *V. destructor*. These transcriptomic insights could be used as markers in future selective breeding strategies aimed at enhancing honey bee resistance to *Varroa.*

## Data Availability

The data from this study will be provided by the corresponding author upon reasonable request. The data are not publicly available due to future publications and collaborations related to the breeding lines.

## Supplementary Materials

The following supporting information can be downloaded at https://www.mdpi.com/article/10.3390/pathogens14111077/s1, Figure S1: Volcano plots of DEGs of pairwise comparisons. (a) LVG-C versus HVG-C. (b) LVG-V versus HVG-V. (c) LVG-V versus LVG-C. (d) HVG-V versus HVG-C. The horizontal line indicates the log2FC, and vertical line indicates the -log10 adjusted *p*-value. The green dots indicate DEGs with log2FC (*p* > 0.05), and the red dots indicate DEGs with log2FC (*p* < 0.05); Figure S2: Gene ontology classification and KEGG pathways of up-regulated DEGs unique to the LVG-V versus HVG-V comparison (*p* < 0.05). The y-axis indicates the GO (biological process, cellular component, and molecular function) and KEGG classification. The x-axis indicates the number of DEGs matching the classification; Table S1: Number of reads, aligned reads to *Apis mellifera* reference genome (Amel_HAv3.1), and GC content of the reads. Treatments were HVG control bees without *Varroa* (HVG-C), LVG control bees without *Varroa* (LVG-C), HVG bees with *Varroa* (HVG-V), and LVG bees with *Varroa* (LVG-V). Each treatment had three replicates; Table S2: Pairwise comparisons of DEGs between treatments based on log2 fold-change (*p* < 0.05); Table S3: Characteristics of up-regulated DEGs unique to HVG-V versus HVG-C comparison (*p* < 0.05, log2FC); Table S4: Characteristics of up-regulated DEGs unique to the LVG-V versus LVG-C comparison (*p* < 0.05, log2FC; Table S5: Characteristics of up-regulated DEGs shared between LVG-V versus LVG-C and HVG-V versus HVG-C comparisons (*p* < 0.05, log2FC); Table S6: Characteristics of up-regulated DEGs unique to the LVG-V versus HVG-V comparison (*p* < 0.05, log2FC); Table S7: Characteristics of down-regulated DEGs unique from HVG-V versus HVG-C comparison (*p* < 0.05, log2FC); Table S8: Characteristics of down-regulated unique DEGs from LVG-V versus LVG-C comparison (*p* < 0.05, log2FC); Table S9; Characteristics of down-regulated DEGs shared between LVG-V versus LVG-C and HVG-V versus HVG-C comparisons (*p* < 0.05, log2FC); Table S10: Characteristics of down-regulated DEGs unique to the LVG-V versus HVG-V comparison (*p* < 0.05, log2FC); Table S11: Characteristics of the down-regulated DEGs unique to the LVG-C versus LVG-C comparison (*p* < 0.05, log2FC); Table S12: Characteristics of down-regulated DEGs shared between LVG-V versus HVG-V, LVG-V versus LVG-C, and HVG-V versus HVG-C comparisons (*p* < 0.05, log2FC).

## Abbreviations

The following abbreviations are used in this manuscript:

ATP	Adenosine triphosphate
bp	Base pairs
CA	California
CO_2_	Carbon dioxide
DEA	Differential expression analysis
DEGs	Differentially expressed genes
Dim	Dimension
DNA	Deoxyribonucleic acid
DWV	Deformed wing virus
FPKM	Kilobase of exon per million fragments mapped
GC	Guanine and cytosine content
GO	Gene ontology
GTP	Guanosine triphosphate
GTPase	Guanosine triphosphatases
H_2_O	Water
HVG	Higher rates of *Varroa* population growth
HVG-C	HVG bees without *Varroa*
HVG-V	HVG bees with *Varroa*
KEGG	Kyoto Encyclopedia of Genes and Genomes
LVG	Lower rates of *Varroa* population growth
LVG-C	LVG bees without *Varroa*
LVG-V	LVG bees with *Varroa*
Log	Logarithm
log2FC	Logarithm 2 fold change
logFC	Logarithm fold change
μL	Microliter
M	Millions
MA	Massachusetts
Min	Minutes
mm	Millimeter
NADH	Nicotinamide adenine dinucleotide (NAD) + hydrogen (H)
ON	Ontario
p	*p*-value
padj	Adjusted *p*-value
PCA	Principal component analysis
PRE	Percent relative error
QC	Québec
RPKM	Kilobase of transcript per million mapped reads
RH	Relative humidity
RNA	Ribonucleic acid
RNA-seq	RNA sequencing
rpm	Revolutions per minute
s	Seconds
srnRNA	Small nuclear RNA
USA	United States of America
*V. destructor*	*Varroa destructor*
VSH	*Varroa* sensitive hygiene
×*g*	G-force
χ^2^	Chi-square test
