# Supplementary material for: Effect of Selection for Low and High Varroa destructor Population Growth Rates on the Honey Bee Transcriptome
Source: Pathogens. 2025 Oct 22;14(11):1077. doi: 10.3390/pathogens14111077 (PMC12655029; doi:10.3390/pathogens14111077)
Supplement: Supplementary file 1 [file pathogens-14-01077-s001.zip › pathogens-3907500-supplementary.pdf]

Supplemental materials

Supplemental Figures

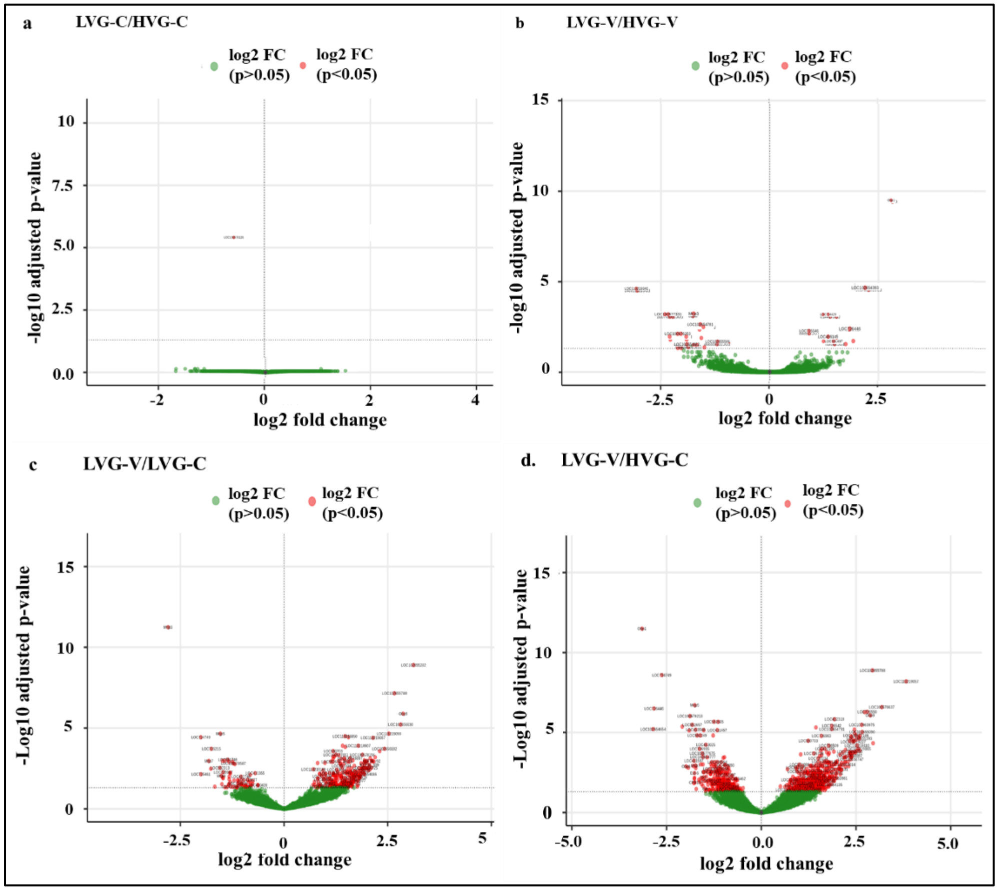

Figure S1. Volcano plots of DEGs of pairwise comparisons. (a) LVG-C versus HVG-C. (b) LVG-V versus HVG-V. (c) LVG-V versus LVG-C. (d) HVG-V versus HVG-C. The horizontal line indicates the log2FC, and vertical line indicates the -log10 adjusted p-value. The green dots indicate DEGs with log2FC ($p>0.05$), and the red dots indicate DEGs with log2FC ($p<0.05$).

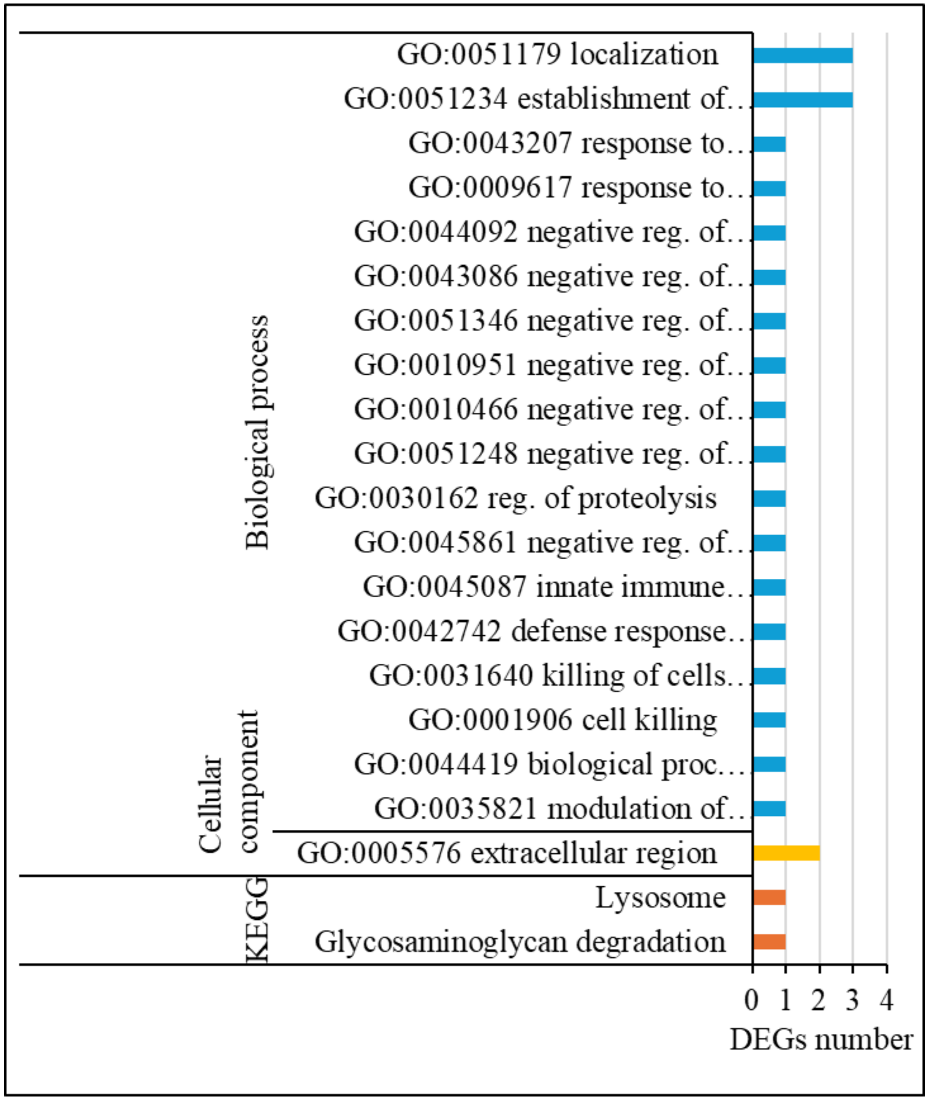

Figure S2. Gene ontology classification and KEGG pathways of up-regulated DEGs unique to the LVG-V versus HVG-V comparison ($p<0.05$). The y-axis indicates the GO (biological process, cellular component, and molecular function) and KEGG classification. The x-axis indicates the number of DEGs matching the classification.

Supplemental Tables

Table S1. Number of reads, aligned reads to *Apis mellifera* reference genome (Amel_HAv3.1), and GC content of the reads. Treatments were HVG control bees without *Varroa* (HVG-C), LVG control bees without *Varroa* (LVG-C), HVG bees with *Varroa* (HVG-V), and LVG bees with *Varroa* (LVG-V). Each treatment had three replicates.

Treatment	Reads (million)	Aligned reads (million)	Aligned reads (%)	GC (%)
HVG-C-1	89.0	46.8	77	43
HVG-C-2	110.1	57.5	80	44
HVG-C-3	102.4	53.9	77	40
LVG-C-1	54.8	29.3	42	44
LVG-C-2	46.4	24.1	31	41
LVG-C-3	36.0	19.0	33	43
HVG-V-1	119.6	63.0	90	44
HVG-V-2	142.2	74.2	91	42
HVG-V-3	33.3	17.5	54	42
LVG-V-1	69.9	36.8	46	42
LVG-V-2	63.5	33.0	38	41
LVG-V-3	72.2	38.0	46	44
Total	939.4	493.1		
Mean	78.3	41.1	58.7	42.3
SEM	10.0	5.2	6.5	0.4
Median	71.1	37.4	50.0	42.3

Table S2. Pairwise comparisons of DEGs between treatments based on Log2 fold-change ($p<0.05$).

Pairwise comparison	Up-regulated DEGs	Down-regulated DEGs	Total DEGs
LVG-C/HVG-C	0	1	1
LVG-V/HVG-V	10	16	26
LVG-V/LVG-C	297	68	365
HVG-V/HVG-C	621	319	940
Total	928	404	1332

Table S3. Characteristics of up-regulated DEGs unique to HVG-V versus HVG-C comparison (p<0.05, log2FC).

Gene symbol[a]	Gene ID[a]	Gene annotation[b]	Genomic accession[a]	Chromosome[a]	Start[a]	End[a]	log2FC[c]	p-value[c]
LOC113219271	113219271	uncharacterized	NC_037638.1	LG1	6867345	6869871	2.3	3.3E-05
LOC100576637	100576637	trypsin 5G1	NC_037643.1	LG6	3156146	3159732	3.2	2.6E-07
LOC113218901	113218901	uncharacterized	NC_037644.1	LG7	7333530	7334715	3.0	4.7E-05
LOC726550	726550	CCAAT/enhancer-binding protein gamma	NC_037641.1	LG4	12159786	12161247	2.8	5.1E-07
LOC102655090	102655090	uncharacterized	NC_037642.1	LG5	13480243	13480911	2.7	9.3E-06
LOC102655028	102655028	uncharacterized	NC_037652.1	LG15	3555362	3559179	2.6	1.4E-04
LOC102653737	102653737	uncharacterized	NC_037643.1	LG6	11213778	11215597	2.5	1.4E-04
LOC102653649	102653649	uncharacterized	NC_037640.1	LG3	11733764	11735202	2.5	2.9E-05
LOC100577146	100577146	uncharacterized	NC_037650.1	LG13	3020578	3028480	2.5	1.5E-05
LOC102655938	102655938	uncharacterized	NC_037653.1	LG16	3525661	3530519	2.5	1.9E-04
LOC100576592	100576592	uncharacterized	NC_037652.1	LG15	4335790	4338152	2.5	6.5E-06
LOC411187	411187	L-lactate dehydrogenase	NC_037644.1	LG7	13960959	13963652	2.4	3.5E-05
LOC107964909	107964909	uncharacterized	NC_037645.1	LG8	7180380	7186236	2.4	2.9E-05
LOC102656111	102656111	uncharacterized	NC_037650.1	LG13	2950100	2953503	2.4	1.9E-04
LOC113218951	113218951	uncharacterized	NC_037645.1	LG8	7117922	7120078	2.4	1.7E-04
LOC113219107	113219107	uncharacterized	NC_037648.1	LG11	10095581	10106715	2.4	3.0E-04
LOC113219341	113219341	small subunit ribosomal RNA	NW_020555813.1	LG9	2142	4064	2.4	3.6E-04
LOC724756	724756	uncharacterized	NC_037648.1	LG11	14814382	14815565	2.4	1.9E-05
LOC102656311	102656311	dynactin subunit 3, transcript variant X1	NC_037646.1	LG9	7020070	7022080	2.4	1.7E-05
LOC102656087	102656087	uncharacterized	NC_037650.1	LG13	2960416	2966777	2.3	2.8E-04
LOC102655426	102655426	uncharacterized	NC_037645.1	LG8	3749749	3752773	2.3	6.7E-04
LOC100577351	100577351	uncharacterized	NC_037645.1	LG8	8574980	8576406	2.2	1.2E-04
LOC102656730	102656730	uncharacterized	NC_037643.1	LG6	11977616	11979884	2.2	7.1E-04
LOC107965353	107965353	uncharacterized	NC_037649.1	LG12	11343594	11348563	2.2	1.4E-03
LOC102655071	102655071	uncharacterized	NC_037647.1	LG10	10952672	10954991	2.2	3.7E-04
LOC411552	411552	ceramide glucosyltransferase	NC_037648.1	LG11	14499154	14502255	2.2	1.7E-04
LOC102653614	102653614	probable RNA methyltransferase CG11342, transcript variant X1	NC_037638.1	LG1	24976696	25011066	2.1	1.0E-03
LOC107964072	107964072	uncharacterized	NC_037639.1	LG2	10854838	10861515	2.1	4.0E-03

LOC113219405	113219405	uncharacterized	NC_037638.1	LG1	1779414	1780586	2.1	1.2E-03
LOC100577819	100577819	uncharacterized	NC_037643.1	LG6	5585899	5588396	2.1	2.1E-03
LOC102654968	102654968	uncharacterized	NC_037649.1	LG12	8061130	8070256	2.1	1.9E-03
LOC107965336	107965336	uncharacterized	NC_037649.1	LG12	6818605	6820124	2.1	1.3E-03
LOC727131	727131	spidroin-2-like	NC_037641.1	LG4	4466926	4468518	2.0	1.9E-03
LOC102654069	102654069	ninjurin-1, transcript variant X1	NC_037647.1	LG10	8165329	8166295	2.0	7.9E-04
Mir3781	100629069	microRNA 3781	NC_037649.1	LG12	11370778	11370887	2.0	3.4E-03
LOC113219308	113219308	uncharacterized	NC_037638.1	LG1	13714224	13726556	2.0	6.8E-03
LOC100576820	100576820	myb-like protein A	NC_037638.1	LG1	1664088	1667022	2.0	7.0E-04
LOC113218934	113218934	uncharacterized	NC_037645.1	LG8	4056914	4058666	2.0	3.8E-03
LOC102655526	102655526	uncharacterized	NC_037647.1	LG10	10983809	10985302	2.0	2.1E-03
LOC100577882	100577882	uncharacterized	NC_037638.1	LG1	27083464	27107604	1.9	9.2E-04
LOC107963981	107963981	uncharacterized	NC_037638.1	LG1	1629816	1631885	1.9	7.1E-03
LOC102653672	102653672	uncharacterized	NC_037646.1	LG9	7431677	7446973	1.9	5.4E-03
LOC102653764	102653764	waprin-Thr1	NC_037642.1	LG5	12616987	12618367	1.9	9.8E-04
LOC412318	412318	AN1-type zinc finger protein 2A, transcript variant X2	NC_037646.1	LG9	298284	300499	1.9	1.5E-06
LOC102655070	102655070	uncharacterized	NC_037643.1	LG6	8777697	8780169	1.9	1.1E-02
LOC102656479	102656479	uncharacterized	NC_037640.1	LG3	226490	227438	1.9	2.1E-03
LOC113219365	113219365	uncharacterized	NW_020555864.1	uncharacterized	66887	69588	1.9	1.0E-02
LOC102654936	102654936	uncharacterized	NC_037649.1	LG12	8076362	8079675	1.9	1.3E-03
LOC107965107	107965107	uncharacterized	NC_037647.1	LG10	4950790	5005145	1.9	5.2E-03
LOC102655465	102655465	uncharacterized	NC_037647.1	LG10	10972250	10975754	1.9	5.8E-03
LOC102654781	102654781	protein G12	NC_037638.1	LG1	13302385	13304070	1.9	6.1E-06
LOC726542	726542	histone H3	NC_037638.1	LG1	15195630	15196174	1.9	3.8E-06
LOC107965072	107965072	uncharacterized	NC_037647.1	LG10	2855929	2893497	1.9	1.7E-02
cact1	725124	NF-kappa-B inhibitor cactus 1	NC_037641.1	LG4	11569669	11576607	1.8	1.3E-03
LOC100577450	100577450	uncharacterized	NC_037638.1	LG1	24176064	24177614	1.8	8.0E-04
LOC102656315	102656315	uncharacterized	NC_037647.1	LG10	10645137	10648146	1.8	6.6E-03
LOC726443	726443	PQ-loop repeat-containing protein 3, transcript variant X2	NC_037648.1	LG11	4271806	4274296	1.8	2.6E-03
LOC113219061	113219061	uncharacterized	NC_037647.1	LG10	6553303	6565044	1.8	6.5E-03

LOC102654135	102654135	uncharacterized	NC_037648.1	LG11	2779039	2780899	1.8	1.8E-02
LOC552403	552403	mediator of RNA polymerase II transcription subunit 10	NC_037646.1	LG9	7545543	7546451	1.8	5.6E-03
LOC725234	725234	yrdC domain-containing protein, mitochondrial	NC_037645.1	LG8	1741846	1742562	1.8	7.9E-04
LOC552047	552047	biogenesis of lysosome-related organelles complex 1 subunit 4, transcript variant X1	NC_037643.1	LG6	15074661	15075805	1.8	1.3E-03
LOC107964436	107964436	RNA-binding motif protein, X-linked 2-like	NC_037642.1	LG5	10942490	10943327	1.8	6.9E-03
LOC102655073	102655073	histone H4	NC_037638.1	LG1	15195053	15195486	1.8	1.9E-04
LOC726449	726449	homologous-pairing protein 2 homolog	NC_037638.1	LG1	5312656	5313472	1.8	5.7E-03
LOC113218732	113218732	uncharacterized	NC_037641.1	LG4	12524101	12526370	1.8	2.3E-02
LOC100578473	100578473	protein FAM177A1	NC_037649.1	LG12	7353461	7354243	1.8	1.7E-03
LOC107966061	107966061	uncharacterized	NC_037638.1	LG1	21474407	21481907	1.8	9.7E-03
LOC726509	726509	protein FRA10AC1	NC_037652.1	LG15	3708301	3710103	1.8	6.6E-05
LOC725175	725175	uncharacterized	NC_037644.1	LG7	13788555	13789619	1.8	7.1E-04
LOC552372	552372	ER membrane protein complex subunit 8/9 homolog	NC_037638.1	LG1	25066388	25067744	1.8	2.8E-03
LOC411058	411058	uncharacterized	NC_037642.1	LG5	1759897	1838178	1.8	3.4E-03
LOC102656154	102656154	uncharacterized	NC_037645.1	LG8	2673333	2685105	1.8	2.4E-04
LOC100578397	100578397	uncharacterized	NC_037639.1	LG2	4685764	4688394	1.8	9.9E-03
LOC102655921	102655921	uncharacterized	NC_037651.1	LG14	8917008	8918706	1.8	1.7E-02
LOC102654367	102654367	uncharacterized	NC_037638.1	LG1	13679443	13680861	1.8	2.4E-02
LOC726427	726427	metallo-beta-lactamase domain-containing protein 1	NC_037647.1	LG10	4692645	4693574	1.8	7.9E-04
LOC413319	413319	dynein regulatory complex subunit 7, transcript variant X2	NC_037642.1	LG5	9583677	9661451	1.7	7.9E-04
LOC550665	550665	transcription initiation factor IIA subunit 2	NC_037639.1	LG2	8134184	8135771	1.7	1.7E-03
LOC552093	552093	methyltransferase-like protein 2-A, transcript variant X1	NC_037640.1	LG3	8206060	8207826	1.7	3.1E-03
LOC102653673	102653673	putative uncharacterized protein DDB_G0271606	NC_037646.1	LG9	8536421	8541685	1.7	6.2E-04
LOC107964040	107964040	uncharacterized	NC_037639.1	LG2	4177532	4179238	1.7	9.3E-03
LOC552555	552555	synaptobrevin homolog YKT6, transcript variant X2	NC_037645.1	LG8	2606518	2607630	1.7	1.9E-03

LOC102653850	102653850	uncharacterized	NC_037653.1	LG16	4497849	4512620	1.7	2.5E-02
LOC100578391	100578391	uncharacterized	NC_037643.1	LG6	17522701	17525441	1.7	6.6E-03
LOC113219046	113219046	uncharacterized	NC_037647.1	LG10	4347142	4353750	1.7	3.2E-02
LOC408348	408348	uncharacterized	NC_037648.1	LG11	10072588	10137469	1.7	1.1E-02
LOC102656577	102656577	CAP-Gly domain-containing linker protein 1, transcript variant X2	NC_037646.1	LG9	916838	921873	1.7	2.9E-03
LOC411757	411757	probable glucosamine 6-phosphate N-acetyltransferase	NC_037642.1	LG5	9547441	9548872	1.7	5.7E-03
LOC102656013	102656013	uncharacterized	NC_037644.1	LG7	2430058	2434456	1.7	4.0E-02
LOC100577809	100577809	uncharacterized	NC_037648.1	LG11	1261168	1263441	1.7	5.4E-03
LOC725835	725835	DNA polymerase epsilon subunit 4, transcript variant X2	NC_037642.1	LG5	12092778	12094191	1.7	1.3E-02
LOC725212	725212	lysM and putative peptidoglycan-binding domain-containing protein 3	NC_037642.1	LG5	5471895	5473163	1.7	8.0E-04
LOC100578864	100578864	uncharacterized	NC_037642.1	LG5	7419179	7420743	1.7	4.8E-03
LOC100576140	100576140	uncharacterized	NC_037647.1	LG10	7066530	7067927	1.7	6.6E-04
LOC724769	724769	protein C10	NC_037648.1	LG11	2480257	2481055	1.7	1.6E-03
LOC100578593	100578593	uncharacterized	NC_037643.1	LG6	5273672	5276186	1.7	5.2E-03
LOC100576506	100576506	uncharacterized	NC_037640.1	LG3	608111	609308	1.7	3.2E-03
LOC107964483	107964483	uncharacterized	NC_037642.1	LG5	2898988	2906595	1.7	2.8E-02
LOC411568	411568	ribosomal oxygenase 1, transcript variant X1	NC_037650.1	LG13	2191879	2233868	1.6	6.1E-03
LOC113218782	113218782	uncharacterized	NC_037642.1	LG5	5386852	5387151	1.6	2.0E-02
LOC724164	724164	39S ribosomal protein L30, mitochondrial	NC_037640.1	LG3	1869787	1870761	1.6	5.4E-03
LOC726222	726222	ATP synthase subunit g, mitochondrial	NC_037643.1	LG6	16449332	16450110	1.6	1.1E-02
LOC102655361	102655361	uncharacterized	NC_037650.1	LG13	4933706	4937034	1.6	1.5E-02
LOC100578455	100578455	uncharacterized	NC_037647.1	LG10	6026432	6057053	1.6	1.6E-02
LOC100576192	100576192	uncharacterized	NC_037646.1	LG9	10090060	10093987	1.6	1.2E-02
LOC102656657	102656657	nucleolar protein of 40 kDa	NC_037642.1	LG5	2994629	2995726	1.6	1.6E-02
LOC551313	551313	calcineurin subunit B type 2	NC_037648.1	LG11	14880279	14881661	1.6	6.5E-03
LOC727431	727431	gustatory receptor for sugar taste 64f, transcript variant X3	NC_037642.1	LG5	10798264	10804745	1.6	2.2E-02

LOC725283	725283	uncharacterized	NC_037638.1	LG1	23248289	23250912	1.6	3.1E-02
LOC113218589	113218589	uncharacterized	NC_037639.1	LG2	11244432	11266012	1.6	2.0E-02
LOC551311	551311	ribosome production factor 2 homolog, transcript variant X1	NC_037642.1	LG5	31398	33049	1.6	1.2E-03
LOC100576106	100576106	uncharacterized	NC_037638.1	LG1	12061960	12066973	1.6	1.3E-03
LOC113219427	113219427	uncharacterized	NC_037639.1	LG2	10847517	10862552	1.6	3.9E-02
LOC100577920	100577920	uncharacterized	NC_037638.1	LG1	27014602	27032962	1.6	1.6E-02
Tert	692346	telomerase reverse transcriptase	NC_037639.1	LG2	2033858	2037192	1.6	1.4E-02
LOC552823	552823	malonyl-CoA decarboxylase, mitochondrial, transcript variant X1	NC_037648.1	LG11	6615757	6619205	1.6	1.0E-04
LOC725584	725584	TBC1 domain family member 7	NC_037652.1	LG15	2854332	2857986	1.6	8.0E-03
LOC102655924	102655924	uncharacterized	NC_037647.1	LG10	10585370	10587103	1.6	9.7E-03
LOC100578067	100578067	enhancer of split mgamma protein, transcript variant X5	NC_037651.1	LG14	2782831	2811422	1.6	7.9E-03
LOC725512	725512	RISC-loading complex subunit TARBP2	NC_037643.1	LG6	3794132	3795996	1.6	2.6E-04
LOC413165	413165	protein phosphatase 1 regulatory subunit 7	NC_037643.1	LG6	16582904	16584792	1.6	3.0E-03
LOC726983	726983	geranylgeranyl transferase type-2 subunit alpha, transcript variant X2	NC_037647.1	LG10	10242097	10246635	1.6	1.6E-05
LOC107965950	107965950	uncharacterized	NC_037650.1	LG13	2380358	2384479	1.6	1.1E-02
LOC411231	411231	general transcription factor IIH subunit 3	NC_037644.1	LG7	2346358	2347968	1.6	3.0E-03
LOC100576967	100576967	histone deacetylase complex subunit SAP18	NW_020555859.1	uncharacterized	300604	301615	1.6	1.4E-04
LOC102656359	102656359	uncharacterized	NC_037638.1	LG1	12521034	12522578	1.6	3.3E-03
LOC107964096	107964096	uncharacterized	NC_037639.1	LG2	13319279	13320118	1.6	6.8E-03
LOC107965062	107965062	uncharacterized	NC_037647.1	LG10	6192450	6265385	1.6	3.8E-03
LOC102654012	102654012	transcription elongation factor 1 homolog	NC_037649.1	LG12	10079564	10081249	1.6	7.9E-03
LOC113219158	113219158	uncharacterized	NC_037649.1	LG12	5239981	5242557	1.6	1.8E-02
LOC102655111	102655111	transmembrane protein 60	NC_037643.1	LG6	7063926	7065695	1.6	2.6E-02
LOC113219359	113219359	autophagy protein 12-like, transcript variant X4	NW_020555859.1	uncharacterized	418980	420752	1.6	3.8E-03
LOC102653619	102653619	uncharacterized	NC_037638.1	LG1	1606222	1614722	1.6	1.2E-02

LOC107964329	107964329	uncharacterized	NC_037641.1	LG4	11559236	11559851	1.6	2.5E-02
LOC725107	725107	mitochondrial genome maintenance exonuclease 1	NC_037647.1	LG10	3558617	3560120	1.5	5.9E-03
LOC113218829	113218829	probable trafficking protein particle complex subunit 2	NC_037643.1	LG6	12149472	12150216	1.5	8.9E-03
LOC113219369	113219369	small subunit ribosomal RNA	NW_020555880.1	uncharacterized	23745	25667	1.5	3.7E-02
LOC725898	725898	HSPB1-associated protein 1	NC_037642.1	LG5	7485562	7487213	1.5	2.6E-03
LOC727515	727515	mitochondrial import inner membrane translocase subunit Tim29	NC_037639.1	LG2	1629539	1631402	1.5	1.3E-02
LOC552227	552227	translocon-associated protein subunit delta	NC_037642.1	LG5	167833	168921	1.5	6.1E-03
LOC102654144	102654144	uncharacterized	NC_037643.1	LG6	16180575	16184980	1.5	4.4E-02
LOC107964874	107964874	uncharacterized	NC_037645.1	LG8	10499279	10505640	1.5	3.7E-02
LOC724846	724846	uncharacterized	NC_037651.1	LG14	6832350	6834110	1.5	1.6E-02
LOC726498	726498	mitochondrial import inner membrane translocase subunit Tim8	NC_037643.1	LG6	2384224	2384945	1.5	3.0E-04
LOC551671	551671	probable oligoribonuclease	NC_037638.1	LG1	15198586	15199661	1.5	3.6E-02
LOC413714	413714	ATP synthase mitochondrial F1 complex assembly factor 2	NC_037649.1	LG12	8639249	8640540	1.5	1.1E-02
LOC410520	410520	uncharacterized	NC_037651.1	LG14	8307144	8309949	1.5	2.6E-02
LOC725049	725049	nuclear nucleic acid-binding protein C1D	NC_037647.1	LG10	11850045	11851069	1.5	6.2E-03
LOC100577967	100577967	uncharacterized	NC_037642.1	LG5	7481970	7483931	1.5	1.7E-02
LOC107964629	107964629	uncharacterized	NC_037643.1	LG6	16753166	16754421	1.5	3.0E-02
LOC102656395	102656395	heat shock transcription factor, Y-linked-like	NC_037638.1	LG1	4746833	4749201	1.5	1.9E-02
LOC102655330	102655330	uncharacterized	NC_037639.1	LG2	11358576	11361627	1.5	3.4E-03
LOC726289	726289	transcription factor AP-1	NC_037646.1	LG9	11100448	11105614	1.5	9.7E-03
LOC552039	552039	uncharacterized	NC_037638.1	LG1	11160361	11161958	1.5	1.5E-02
LOC727156	727156	vesicle transport protein USE1, transcript variant X2	NC_037642.1	LG5	2264789	2266329	1.5	1.2E-03
LOC102654085	102654085	uncharacterized	NC_037639.1	LG2	7525973	7532551	1.5	2.2E-02
LOC107965288	107965288	probable protein BRICK1-B	NC_037648.1	LG11	6397598	6402098	1.5	6.0E-03
LOC552161	552161	mediator of RNA polymerase II transcription subunit 9	NC_037642.1	LG5	155998	157349	1.5	2.4E-03
LOC726584	726584	syntaxin-1A	NC_037640.1	LG3	6099531	6106329	1.5	3.6E-03

LOC413612	413612	uncharacterized	NC_037645.1	LG8	6971030	6972077	1.5	9.6E-03
LOC102656071	102656071	uncharacterized	NC_037648.1	LG11	1312919	1316116	1.5	1.8E-02
LOC724221	724221	uncharacterized	NC_037643.1	LG6	14523108	14524378	1.5	7.0E-03
LOC107965631	107965631	uncharacterized	NC_037652.1	LG15	5331893	5336052	1.5	2.6E-02
LOC100578943	100578943	transmembrane protein 258	NC_037648.1	LG11	4407292	4408500	1.5	1.9E-02
LOC727300	727300	low molecular weight phosphotyrosine protein phosphatase, transcript variant X2	NC_037639.1	LG2	8162978	8163904	1.5	3.6E-03
LOC411387	411387	O-acyltransferase like protein, transcript variant X1	NC_037643.1	LG6	13121140	13139358	1.5	1.7E-03
LOC727234	727234	cytidine deaminase, transcript variant X1	NC_037648.1	LG11	14860822	14862036	1.5	4.2E-04
CUTA	552415	cutA divalent cation tolerance homolog	NC_037651.1	LG14	5069700	5071257	1.5	3.5E-03
LOC107964352	107964352	uncharacterized	NC_037641.1	LG4	12445108	12446730	1.5	4.4E-02
LOC551963	551963	protein spindle-F, transcript variant X2	NC_037649.1	LG12	2818711	2820951	1.5	6.6E-03
LOC725053	725053	protein odd-skipped	NC_037650.1	LG13	10057492	10060996	1.5	1.2E-02
LOC107964726	107964726	uncharacterized	NC_037644.1	LG7	10396715	10484817	1.5	2.0E-02
LOC726279	726279	M-phase phosphoprotein 6	NC_037653.1	LG16	5271408	5272372	1.5	2.3E-02
LOC102656373	102656373	uncharacterized	NC_037645.1	LG8	6717446	6718605	1.5	3.7E-02
LOC406132	406132	histone H4	NC_037638.1	LG1	15189245	15189759	1.5	1.1E-02
LOC102656336	102656336	uncharacterized	NC_037648.1	LG11	10073094	10111397	1.5	4.9E-02
LOC113218542	113218542	uncharacterized	NC_037639.1	LG2	15431056	15435042	1.5	6.3E-03
LOC727631	727631	uncharacterized	NC_037650.1	LG13	2129040	2130332	1.5	1.2E-02
LOC100577822	100577822	uncharacterized	NC_037638.1	LG1	4952466	4964093	1.5	1.6E-02
LOC107964776	107964776	uncharacterized	NC_037644.1	LG7	12413615	12416320	1.5	4.7E-02
LOC552807	552807	prefoldin subunit 6, transcript variant X2	NC_037652.1	LG15	7421168	7422328	1.5	1.8E-03
LOC410676	410676	adenylate kinase isoenzyme 6	NC_037638.1	LG1	21488281	21489212	1.5	1.3E-02
LOC100577899	100577899	DNA replication complex GINS protein SLD5	NC_037652.1	LG15	7413080	7413785	1.5	1.7E-02
LOC725253	725253	MICOS complex subunit MIC13	NC_037641.1	LG4	7858840	7859814	1.5	1.4E-02
LOC725867	725867	translin-associated protein X	NC_037642.1	LG5	10864691	10866133	1.5	1.9E-02
LOC102656896	102656896	uncharacterized	NC_037643.1	LG6	16655279	16658008	1.5	2.8E-02

LOC113218983	113218983	60S ribosome subunit biogenesis protein NIP7 homolog	NC_037646.1	LG9	1143873	1146838	1.5	9.3E-03
LOC552827	552827	probable small nuclear ribonucleoprotein G	NC_037652.1	LG15	7247225	7247957	1.5	1.1E-02
LOC551996	551996	vesicle transport through interaction with t-SNAREs homolog 1B	NC_037646.1	LG9	6600318	6602315	1.5	2.1E-02
LOC113218525	113218525	uncharacterized	NC_037650.1	LG13	2999152	3006457	1.5	1.5E-02
LOC113219015	113219015	uncharacterized	NC_037646.1	LG9	8629514	8630296	1.5	3.3E-02
LOC113219027	113219027	uncharacterized	NC_037647.1	LG10	1907019	1962651	1.5	4.4E-02
LOC411408	411408	CDP-diacylglycerol--inositol 3-phosphatidyltransferase, transcript variant X1	NC_037638.1	LG1	5647180	5649218	1.5	1.4E-02
LOC113218653	113218653	origin recognition complex subunit 5	NC_037640.1	LG3	611272	613015	1.5	4.7E-02
LOC725135	725135	phosphatidylinositol-glycan biosynthesis class X protein	NC_037645.1	LG8	12602540	12604446	1.5	9.1E-03
LOC113219117	113219117	uncharacterized	NC_037648.1	LG11	13808554	13814108	1.5	4.9E-02
LOC727074	727074	signal peptidase complex subunit 3	NC_037638.1	LG1	6582305	6583974	1.5	1.5E-02
LOC100576898	100576898	esterase CG5412	NC_037641.1	LG4	2834350	2835455	1.5	2.2E-02
LOC102655676	102655676	uncharacterized	NC_037641.1	LG4	11131275	11145491	1.5	8.2E-03
LOC100577923	100577923	protein LLP homolog	NC_037643.1	LG6	3698509	3699590	1.5	9.7E-03
LOC724780	724780	G-protein coupled receptor Mth2	NC_037640.1	LG3	12650746	12655857	1.4	1.1E-03
LOC411085	411085	ADP-ribosylation factor-like protein 2	NC_037642.1	LG5	7487253	7488426	1.4	1.6E-02
LOC412160	412160	programmed cell death protein 10	NC_037640.1	LG3	4461188	4462563	1.4	2.6E-03
LOC102654007	102654007	uncharacterized	NC_037653.1	LG16	5207231	5207707	1.4	2.5E-02
LOC102654029	102654029	uncharacterized	NC_037647.1	LG10	10317615	10321527	1.4	7.3E-03
LOC551392	551392	U11/U12 small nuclear ribonucleoprotein 48 kDa protein	NC_037650.1	LG13	2541510	2543323	1.4	2.4E-02
LOC100578025	100578025	probable serine/threonine-protein kinase samkC	NC_037645.1	LG8	9112870	9114134	1.4	5.0E-02
LOC551358	551358	dehydrodolichyl diphosphate synthase complex subunit DHDDS, transcript variant X3	NC_037638.1	LG1	21028799	21030301	1.4	5.4E-03

LOC726020	726020	protein OSCP1	NC_037651.1	LG14	5980413	5981958	1.4	3.3E-03
LOC100577430	100577430	uncharacterized	NC_037651.1	LG14	3035200	3038713	1.4	5.0E-03
LOC724631	724631	60S ribosomal protein L29	NC_037644.1	LG7	3804858	3806666	1.4	1.8E-02
LOC102654917	102654917	uncharacterized	NC_037638.1	LG1	6300717	6303236	1.4	3.3E-02
LOC100576457	100576457	probable ribosome biogenesis protein RLP24	NC_037653.1	LG16	1496832	1497717	1.4	1.2E-02
LOC107963968	107963968	inositol polyphosphate 1-phosphatase	NC_037648.1	LG11	8294927	8297991	1.4	6.8E-03
LOC113219226	113219226	protein AF-9-like	NC_037651.1	LG14	2313548	2316311	1.4	2.6E-02
LOC552652	552652	origin recognition complex subunit 4, transcript variant X1	NC_037651.1	LG14	8269157	8271385	1.4	3.8E-02
LOC102654882	102654882	uncharacterized	NC_037640.1	LG3	12339560	12340755	1.4	3.7E-02
LOC102654763	102654763	uncharacterized	NC_037641.1	LG4	12058127	12059558	1.4	4.2E-02
Y-e3	413894	yellow-e3	NC_037648.1	LG11	2259179	2265574	1.4	3.1E-02
LOC552583	552583	guanosine-3',5'-bis(diphosphate) 3'-pyrophosphohydrolase MESH1	NC_037641.1	LG4	145320	146338	1.4	1.1E-02
LOC552645	552645	mesencephalic astrocyte-derived neurotrophic factor homolog	NC_037643.1	LG6	6640139	6642057	1.4	1.3E-03
LOC552359	552359	active regulator of SIRT1	NC_037646.1	LG9	11437172	11438036	1.4	3.6E-03
LOC411862	411862	28S ribosomal protein S2, mitochondrial	NC_037648.1	LG11	14981223	14982597	1.4	3.3E-03
LOC102656247	102656247	dynein-1-beta heavy chain, flagellar inner arm I1 complex	NC_037639.1	LG2	8155907	8159516	1.4	2.7E-02
Mir3727	100629084	microRNA 3727	NC_037641.1	LG4	12894313	12894378	1.4	6.2E-03
Mir283	100315665	microRNA 283	NC_037639.1	LG2	4842179	4842278	1.4	4.7E-02
LOC727568	727568	dolichyl-diphosphooligosaccharide--protein glycosyltransferase subunit DAD1	NC_037646.1	LG9	889368	890637	1.4	2.5E-02
LOC726085	726085	probable prefoldin subunit 2	NC_037639.1	LG2	6933649	6934760	1.4	3.9E-03
LOC100577454	100577454	tuftelin	NC_037638.1	LG1	4569289	4571668	1.4	2.5E-02
LOC102655862	102655862	uncharacterized	NC_037640.1	LG3	5400593	5407875	1.4	3.4E-03
LOC551541	551541	uncharacterized	NC_037641.1	LG4	13154850	13155889	1.4	2.3E-02

LOC412589	412589	BRISC and BRCA1-A complex member 2	NC_037641.1	LG4	5497587	5499371	1.4	2.2E-02
LOC552846	552846	dnaJ homolog subfamily C member 17	NC_037642.1	LG5	13823766	13825772	1.4	2.8E-02
LOC100577428	100577428	uncharacterized	NC_037650.1	LG13	3595213	3703506	1.4	3.2E-02
LOC725764	725764	peroxisomal membrane protein PEX16	NC_037645.1	LG8	1988410	1989830	1.4	2.5E-03
LOC410632	410632	G protein-coupled receptor kinase 2, transcript variant X1	NC_037652.1	LG15	3540252	3561158	1.4	3.0E-02
LOC102653993	102653993	transcription factor Sox-21-B-like	NC_037648.1	LG11	11382918	11387422	1.4	4.9E-02
LOC552780	552780	ribonuclease P/MRP protein subunit POP5	NC_037642.1	LG5	9838677	9839814	1.4	2.5E-02
LOC107965509	107965509	uncharacterized	NC_037651.1	LG14	4594920	4596302	1.4	4.7E-02
LOC725708	725708	uncharacterized	NC_037644.1	LG7	2336058	2343384	1.4	2.0E-04
LOC413128	413128	putative ferric-chelate reductase 1 homolog, transcript variant X1	NC_037642.1	LG5	4934716	4951322	1.4	9.3E-03
LOC102656685	102656685	39S ribosomal protein L42, mitochondrial, transcript variant X1	NC_037646.1	LG9	1599501	1600533	1.4	2.3E-02
LOC724923	724923	SAC3 domain-containing protein 1	NC_037641.1	LG4	12767975	12770351	1.4	1.5E-02
LOC414008	414008	ribonucleoside-diphosphate reductase subunit M2, transcript variant X3	NC_037652.1	LG15	2549398	2552687	1.4	2.9E-02
LOC102656636	102656636	uncharacterized	NC_037638.1	LG1	6962041	6964883	1.4	1.7E-02
LOC410890	410890	protein seele	NC_037639.1	LG2	6608378	6609369	1.4	2.8E-02
LOC100577648	100577648	uncharacterized	NC_037638.1	LG1	22609147	22610443	1.4	1.5E-02
LOC726711	726711	ribonuclease H2 subunit C	NC_037639.1	LG2	10203023	10203797	1.4	3.2E-02
LOC102653751	102653751	uncharacterized	NC_037640.1	LG3	12647762	12649629	1.4	1.7E-03
LOC727031	727031	vacuolar protein sorting-associated protein 45	NC_037652.1	LG15	9040376	9046026	1.4	2.8E-02
LOC724372	724372	nucleolar protein 12, transcript variant X1	NC_037638.1	LG1	1816644	1818580	1.4	3.1E-02
LOC107965746	107965746	uncharacterized	NC_037650.1	LG13	2098862	2101663	1.4	3.3E-03
LOC113219060	113219060	uncharacterized	NC_037647.1	LG10	6529703	6734837	1.4	3.1E-02
LOC100576878	100576878	uncharacterized	NC_037645.1	LG8	4058816	4063083	1.4	1.1E-02
LOC113219136	113219136	transmembrane protein 177, transcript variant X2	NC_037648.1	LG11	11058444	11060919	1.4	1.7E-02

LOC100578483	100578483	probable DNA primase large subunit	NC_037644.1	LG7	12059888	12061517	1.4	2.0E-02
LOC107966054	107966054	uncharacterized	NC_037638.1	LG1	20608879	20609699	1.4	4.6E-02
LOC107964964	107964964	uncharacterized	NC_037646.1	LG9	5857786	5859693	1.4	3.7E-03
LOC411607	411607	pterin-4-alpha-carbinolamine dehydratase	NC_037653.1	LG16	7166728	7168206	1.4	2.9E-02
LOC100578731	100578731	uncharacterized	NC_037647.1	LG10	10054932	10056782	1.4	4.7E-03
LOC551413	551413	U3 small nucleolar ribonucleoprotein protein IMP3	NC_037652.1	LG15	2746709	2747683	1.3	2.9E-02
LOC102653797	102653797	uncharacterized	NC_037640.1	LG3	9983658	9988495	1.3	1.6E-02
LOC413928	413928	39S ribosomal protein L13, mitochondrial	NC_037639.1	LG2	6330986	6332319	1.3	9.0E-03
LOC412913	412913	Golgi SNAP receptor complex member 2	NC_037647.1	LG10	8402961	8404264	1.3	3.6E-02
LOC100576960	100576960	DNA polymerase subunit gamma-2, mitochondrial	NC_037644.1	LG7	8712969	8714124	1.3	1.5E-02
LOC725572	725572	ribonuclease kappa-B	NC_037638.1	LG1	20639154	20640123	1.3	8.3E-03
LOC102656089	102656089	uncharacterized	NC_037641.1	LG4	7946444	7952984	1.3	3.8E-02
LOC724708	724708	39S ribosomal protein L23, mitochondrial	NC_037645.1	LG8	6253772	6254681	1.3	4.2E-02
LOC725208	725208	E3 ubiquitin-protein ligase RAD18	NC_037641.1	LG4	7855740	7858573	1.3	5.8E-03
LOC724203	724203	ribosome biogenesis protein BRX1 homolog	NC_037649.1	LG12	7690123	7691754	1.3	1.7E-02
LOC552514	552514	HD domain-containing protein 2, transcript variant X2	NC_037646.1	LG9	11373156	11374689	1.3	1.2E-02
LOC726196	726196	ubiquitin-fold modifier 1, transcript variant X2	NC_037644.1	LG7	1364924	1366908	1.3	2.6E-03
LOC107964843	107964843	uncharacterized	NC_037645.1	LG8	9366920	9378748	1.3	4.7E-02
LOC100576088	100576088	28S ribosomal protein S23, mitochondrial	NC_037638.1	LG1	1682530	1683635	1.3	2.5E-02
LOC100578119	100578119	protein MIS12 homolog, transcript variant X1	NC_037639.1	LG2	9532068	9533725	1.3	6.1E-03
LOC551354	551354	exocyst complex component 4	NC_037638.1	LG1	11485404	11486356	1.3	4.5E-02
LOC413735	413735	glutamate--cysteine ligase regulatory subunit, transcript variant X1	NC_037638.1	LG1	5227396	5232081	1.3	1.9E-02
LOC552840	552840	deoxynucleoside kinase	NC_037642.1	LG5	13676271	13677738	1.3	2.6E-02

LOC100577216	100577216	uncharacterized	NC_037640.1	LG3	2104	38014	1.3	4.5E-02
LOC726480	726480	39S ribosomal protein L54, mitochondrial	NC_037642.1	LG5	6550661	6551456	1.3	3.4E-02
LOC107964891	107964891	THO complex subunit 7 homolog, transcript variant X2	NC_037645.1	LG8	1440022	1441045	1.3	1.1E-02
LOC551511	551511	elongin-B	NC_037641.1	LG4	2836827	2839141	1.3	4.7E-03
LOC551557	551557	uncharacterized	NC_037638.1	LG1	10716502	10718000	1.3	2.3E-02
LOC100576289	100576289	uncharacterized	NC_037648.1	LG11	1510608	1511532	1.3	4.8E-02
LOC726385	726385	GTPase Era, mitochondrial	NC_037640.1	LG3	8195900	8197168	1.3	1.9E-02
LOC726962	726962	trafficking protein particle complex subunit 1	NC_037652.1	LG15	8841099	8842169	1.3	7.4E-03
LOC551158	551158	28S ribosomal protein S17, mitochondrial	NC_037638.1	LG1	21014254	21015626	1.3	2.3E-02
LOC100577128	100577128	uncharacterized	NC_037642.1	LG5	12083816	12085215	1.3	7.9E-03
LOC726417	726417	uncharacterized	NC_037652.1	LG15	5001602	5004008	1.3	1.2E-03
LOC724628	724628	protein mago nashi homolog	NC_037640.1	LG3	4591687	4592531	1.3	3.3E-03
LOC100577623	100577623	putative uncharacterized protein DDB_G0282133	NC_037648.1	LG11	15817648	15819096	1.3	1.3E-03
LOC100576568	100576568	SWR1-complex protein 3, transcript variant X2	NC_037643.1	LG6	13225696	13233194	1.3	3.7E-02
LOC726220	726220	pyroglutamyl-peptidase 1	NC_037638.1	LG1	14358547	14359333	1.3	4.6E-02
LOC113218642	113218642	uncharacterized	NC_037640.1	LG3	10015106	10016264	1.3	4.8E-02
LOC552152	552152	28S ribosomal protein S24, mitochondria	NC_037644.1	LG7	5286337	5287361	1.3	1.6E-02
LOC107965817	107965817	DNA replication complex GINS protein PSF3	NC_037646.1	LG9	514173	521904	1.3	3.8E-02
LOC725880	725880	small nuclear ribonucleoprotein F, transcript variant X1	NC_037647.1	LG10	6315325	6317612	1.3	3.6E-02
LOC724647	724647	uncharacterized	NC_037652.1	LG15	2598549	2603420	1.3	4.6E-02
LOC551695	551695	gamma-secretase subunit pen-2	NC_037649.1	LG12	7799357	7800551	1.3	5.4E-03
LOC107966102	107966102	uncharacterized	NC_037639.1	LG2	9967997	9971106	1.3	2.0E-02
LOC102653949	102653949	uncharacterized	NC_037650.1	LG13	6829788	6830570	1.3	1.9E-02
LOC726760	726760	inhibitor of nuclear factor kappa-B kinase subunit beta	NC_037639.1	LG2	2549021	2552947	1.3	2.5E-02
LOC412363	412363	cilia- and flagella-associated protein 20	NC_037647.1	LG10	8051348	8052981	1.3	3.0E-02

LOC409451	409451	thioredoxin-2	NC_037643.1	LG6	6611748	6613808	1.3	1.2E-03
LOC724619	724619	cyclin-dependent kinase 2	NC_037650.1	LG13	1492946	1531475	1.3	2.9E-02
LOC410597	410597	SET and MYND domain-containing protein 5	NC_037652.1	LG15	7441996	7443724	1.3	3.0E-02
LOC100576552	100576552	uncharacterized	NC_037648.1	LG11	396422	402823	1.3	2.3E-02
LOC724633	724633	histone H2B	NC_037638.1	LG1	11690472	11690998	1.3	1.3E-02
LOC726132	726132	WASH complex subunit 3, transcript variant X1	NC_037641.1	LG4	11116267	11118026	1.3	3.8E-02
LOC725383	725383	uncharacterized	NC_037638.1	LG1	9068210	9069068	1.3	2.1E-02
LOC726696	726696	uncharacterized	NC_037650.1	LG13	2840749	2842950	1.3	2.5E-03
LOC107964502	107964502	uncharacterized	NC_037638.1	LG1	11124497	11125070	1.3	4.4E-02
LOC107965117	107965117	uncharacterized	NC_037647.1	LG10	6513742	6517461	1.3	4.4E-02
LOC410591	410591	splicing regulator RBM11	NC_037652.1	LG15	7561282	7562315	1.3	1.7E-02
LOC100577575	100577575	uncharacterized	NC_037645.1	LG8	9834089	9835871	1.3	2.9E-02
LOC726840	726840	BET1 homolog	NC_037639.1	LG2	10378119	10379484	1.3	2.3E-02
LOC100579054	100579054	uncharacterized	NC_037638.1	LG1	24238474	24241525	1.3	4.5E-02
LOC100578975	100578975	dynein regulatory complex subunit 3	NC_037644.1	LG7	12309211	12312696	1.3	4.5E-02
LOC412262	412262	protein Hook homolog 3	NC_037638.1	LG1	21025148	21027811	1.3	4.5E-02
LOC724728	724728	NF-kappa-B essential modulator	NC_037650.1	LG13	9602590	9605068	1.3	4.4E-02
LOC725947	725947	U6 snRNA-associated Sm-like protein LSm6	NC_037638.1	LG1	22593637	22594369	1.3	4.4E-02
LOC413194	413194	myeloid differentiation primary response protein MyD88-A, transcript variant X2	NC_037640.1	LG3	4964887	4969488	1.2	8.6E-03
LOC726380	726380	protein-S-isoprenylcysteine O-methyltransferase	NC_037645.1	LG8	7002617	7004038	1.2	2.8E-02
LOC552791	552791	MIP18 family protein galla-2	NC_037648.1	LG11	11912857	11913779	1.2	4.2E-03
LOC552038	552038	synaptosomal-associated protein 29	NC_037638.1	LG1	6019689	6021000	1.2	2.5E-02
LOC552686	552686	breast cancer metastasis-suppressor 1-like protein, transcript variant X1	NC_037650.1	LG13	2414635	2416093	1.2	2.9E-02
LOC552509	552509	MKI67 FHA domain-interacting nucleolar phosphoprotein-like	NC_037644.1	LG7	8662237	8663478	1.2	8.3E-03
Ho	494506	heme oxygenase	NC_037644.1	LG7	3795733	3797323	1.2	8.7E-03

LOC100577466	100577466	uncharacterized	NC_037647.1	LG10	6310678	6312061	1.2	1.1E-02
LOC724363	724363	activator of basal transcription 1	NC_037640.1	LG3	252964	254289	1.2	3.2E-03
LOC412067	412067	cysteine and histidine-rich domain-containing protein	NC_037641.1	LG4	2831417	2833436	1.2	2.8E-02
LOC100578277	100578277	U11/U12 small nuclear ribonucleoprotein 25 kDa protein	NC_037653.1	LG16	6629077	6630089	1.2	9.2E-03
LOC724842	724842	elongator complex protein 5, transcript variant X1	NC_037648.1	LG11	14831018	14832401	1.2	1.8E-02
LOC725904	725904	uncharacterized	NC_037641.1	LG4	5702860	5704644	1.2	3.2E-03
LOC113219426	113219426	uncharacterized	NC_037639.1	LG2	15245501	15249459	1.2	3.3E-02
LOC100576559	100576559	uncharacterized	NC_037642.1	LG5	12097635	12099114	1.2	4.7E-02
LOC724659	724659	ribonuclease P protein subunit rpr2	NC_037644.1	LG7	8138252	8139460	1.2	4.8E-02
LOC410253	410253	ataxin-2 homolog, transcript variant X1	NC_037648.1	LG11	13686610	13708311	1.2	4.5E-02
LOC552596	552596	peroxisome assembly protein 12, transcript variant X1	NC_037645.1	LG8	2246610	2249072	1.2	4.8E-02
LOC107964193	107964193	uncharacterized	NC_037640.1	LG3	9559693	9576036	1.2	4.7E-02
LOC724943	724943	phosphatidylinositol glycan anchor biosynthesis class U protein	NC_037649.1	LG12	3960209	3962005	1.2	1.9E-02
LOC724329	724329	dnaJ homolog subfamily C member 24	NC_037638.1	LG1	10776397	10779161	1.2	3.6E-02
LOC100577451	100577451	glycolipid transfer protein	NC_037650.1	LG13	2494080	2495764	1.2	4.1E-02
LOC107965557	107965557	uncharacterized	NC_037652.1	LG15	4331676	4333709	1.2	1.7E-02
LOC726298	726298	putative transcription factor SOX-15, transcript variant X1	NC_037638.1	LG1	5148614	5200368	1.2	4.6E-02
LOC100578559	100578559	zinc finger protein 724-like	NC_037645.1	LG8	3862027	3865099	1.2	4.6E-02
LOC551260	551260	uncharacterized	NC_037644.1	LG7	1481264	1482615	1.2	1.9E-03
LOC724144	724144	enhancer of rudimentary homolog	NC_037638.1	LG1	6622483	6623792	1.2	3.6E-02
LOC102655139	102655139	uncharacterized	NC_037642.1	LG5	6867462	6869337	1.2	2.8E-02
LOC725918	725918	39S ribosomal protein L53, mitochondrial	NC_037650.1	LG13	2163239	2165825	1.2	2.6E-02
LOC727490	727490	E3 ubiquitin-protein ligase FANCL	NC_037649.1	LG12	7839684	7841698	1.2	9.5E-03
LOC100579024	100579024	D-aminoacyl-tRNA deacylase 1	NC_037639.1	LG2	4566396	4567602	1.2	4.0E-02

LOC100576286	100576286	26S proteasome non-ATPase regulatory subunit 6-like	NC_037648.1	LG11	14515703	14518025	1.2	3.8E-02
LOC725807	725807	AP-2 complex subunit sigma, transcript variant X2	NC_037646.1	LG9	1626451	1627349	1.2	4.4E-02
LOC107965926	107965926	uncharacterized	NC_037643.1	LG6	15582717	15583789	1.2	2.8E-02
LOC727520	727520	uncharacterized	NC_037644.1	LG7	10650400	10655262	1.2	3.2E-02
LOC551416	551416	uncharacterized	NC_037638.1	LG1	7054573	7057362	1.2	2.6E-02
LOC727354	727354	vacuolar protein sorting-associated protein VTA1 homolog, transcript variant X1	NC_037640.1	LG3	326214	332656	1.2	4.8E-02
LOC726120	726120	ATP synthase-coupling factor 6, mitochondrial	NC_037647.1	LG10	8325656	8326502	1.2	4.6E-02
LOC725951	725951	crossover junction endonuclease MUS81	NC_037648.1	LG11	11113080	11115912	1.2	4.8E-02
LOC550926	550926	phosphomannomutase, transcript variant X2	NC_037648.1	LG11	6749205	6751514	1.2	3.8E-02
LOC411870	411870	microtubule-associated proteins 1A/1B light chain 3A, transcript variant X1	NC_037639.1	LG2	9503534	9505194	1.2	3.5E-02
LOC726265	726265	lysM and putative peptidoglycan-binding domain-containing protein 2, transcript variant X1	NC_037647.1	LG10	6406568	6408357	1.2	3.4E-02
LOC100578428	100578428	lipid storage droplets surface-binding protein 2, transcript variant X1	NC_037646.1	LG9	2559002	2560715	1.2	3.1E-02
LOC725409	725409	7-methylguanosine phosphate-specific 5'-nucleotidase	NC_037642.1	LG5	370466	373890	1.2	2.6E-02
LOC726061	726061	mitochondrial import inner membrane translocase subunit Tim10, transcript variant X3	NC_037638.1	LG1	20142640	20143585	1.2	4.6E-02
LOC725291	725291	probable 28S ribosomal protein S16, mitochondrial	NC_037651.1	LG14	5221393	5222719	1.2	4.9E-02
LOC107964581	107964581	uncharacterized	NC_037643.1	LG6	7783967	7785653	1.2	2.2E-02
LOC552102	552102	proteasome assembly chaperone 1	NC_037642.1	LG5	5394339	5395460	1.2	3.6E-02
LOC727612	727612	PITH domain-containing protein GA19395, transcript variant X2	NC_037649.1	LG12	8656051	8657620	1.2	4.1E-02
LOC100577823	100577823	uncharacterized	NC_037653.1	LG16	7144321	7146299	1.2	3.1E-02

LOC102653641	102653641	glomulin	NC_037644.1	LG7	13758539	13760800	1.2	4.3E-02
LOC410087	410087	uncharacterized	NC_037639.1	LG2	4820592	4826090	1.2	2.6E-03
LOC724638	724638	H/ACA ribonucleoprotein complex subunit 3	NC_037638.1	LG1	21054667	21055385	1.1	3.0E-02
LOC726303	726303	N-acetyltransferase family 8 member 3	NC_037639.1	LG2	5965027	5966261	1.1	4.7E-02
LOC552268	552268	vesicle transport protein SFT2B, transcript variant X1	NC_037649.1	LG12	5217708	5219014	1.1	3.6E-02
LOC552659	552659	optic atrophy 3 protein homolog	NC_037642.1	LG5	9554708	9556273	1.1	3.5E-02
LOC107965322	107965322	gamma-interferon-inducible lysosomal thiol reductase	NC_037649.1	LG12	8583116	8584860	1.1	3.5E-03
LOC551651	551651	ferritin subunit	NC_037651.1	LG14	5961153	5965014	1.1	2.9E-02
LOC107966069	107966069	uncharacterized	NC_037640.1	LG3	1982740	1984798	1.1	3.7E-02
LOC413869	413869	retinol dehydrogenase 11	NC_037639.1	LG2	4514526	4516478	1.1	1.1E-02
LOC726689	726689	prefoldin subunit 1	NC_037645.1	LG8	7742770	7743663	1.1	8.7E-03
LOC552097	552097	39S ribosomal protein L14, mitochondrial	NC_037647.1	LG10	10214141	10215402	1.1	3.6E-02
LOC725700	725700	vacuolar protein sorting-associated protein 29	NC_037640.1	LG3	13291851	13293194	1.1	2.5E-02
LOC725468	725468	AN1-type zinc finger protein 1, transcript variant X1	NC_037652.1	LG15	5773415	5774845	1.1	4.5E-02
LOC552422	552422	trafficking protein particle complex subunit 13	NC_037639.1	LG2	6285523	6288358	1.1	3.7E-02
LOC102656645	102656645	uncharacterized	NC_037645.1	LG8	5271657	5273082	1.1	3.7E-02
LOC100576744	100576744	uncharacterized	NC_037648.1	LG11	15339974	15342409	1.1	4.7E-02
LOC727466	727466	XK-related protein 6	NC_037641.1	LG4	5492763	5494420	1.1	2.9E-02
LOC410557	410557	ATP synthase subunit d, mitochondrial	NC_037651.1	LG14	9962901	9964072	1.1	2.3E-02
LOC726363	726363	2-(3-amino-3-carboxypropyl)histidine synthase subunit 2	NC_037644.1	LG7	12091299	12093546	1.1	3.2E-03
LOC412277	412277	mRNA cap guanine-N7 methyltransferase, transcript variant X1	NC_037648.1	LG11	12191134	12194076	1.1	3.2E-02
LOC726309	726309	protein artichoke	NC_037642.1	LG5	8275530	8283257	1.1	4.4E-02
LOC113219283	113219283	ATP synthase mitochondrial F1 complex assembly factor 1	NC_037652.1	LG15	7383549	7384714	1.1	4.8E-02
LOC726900	726900	proteasome assembly chaperone 2, transcript variant X2	NC_037642.1	LG5	168905	170274	1.1	6.1E-03

LOC411546	411546	FGFR1 oncogene partner 2 homolog	NC_037638.1	LG1	25271142	25273036	1.1	4.7E-02
LOC100576348	100576348	uncharacterized	NC_037646.1	LG9	1642696	1644343	1.1	2.3E-02
LOC551692	551692	protein FAM50 homolog	NC_037642.1	LG5	8531552	8532956	1.1	4.6E-02
LOC725138	725138	copper transport protein ATOX1	NC_037639.1	LG2	15245160	15245838	1.1	4.1E-02
LOC409595	409595	uncharacterized	NC_037648.1	LG11	14840591	14842246	1.1	3.2E-02
LOC725866	725866	39S ribosomal protein L9, mitochondrial	NC_037642.1	LG5	7484352	7485385	1.1	1.2E-02
LOC107965063	107965063	uncharacterized	NC_037638.1	LG1	21737885	21742543	1.1	8.0E-03
LOC100578727	100578727	uncharacterized	NC_037639.1	LG2	5502188	5525609	1.1	4.2E-02
LOC551149	551149	myosin-2 essential light chain, transcript variant X3	NC_037650.1	LG13	2426959	2428617	1.1	1.5E-02
LOC725370	725370	uncharacterized	NC_037645.1	LG8	8220609	8222321	1.1	1.6E-02
LOC552042	552042	rab-like protein 3, transcript variant X1	NC_037647.1	LG10	9413604	9415159	1.1	3.7E-02
LOC552803	552803	histidine triad nucleotide-binding protein 3	NC_037646.1	LG9	10892299	10893388	1.1	1.8E-02
LOC726731	726731	probable 28S ribosomal protein S26, mitochondrial	NC_037652.1	LG15	6551904	6552904	1.1	4.7E-02
LOC726708	726708	F-box only protein 28	NC_037638.1	LG1	20578937	20580861	1.1	4.7E-02
LOC100576266	100576266	uncharacterized	NC_037649.1	LG12	7671030	7675691	1.1	4.8E-02
LOC724167	724167	putative uncharacterized protein DDB_G0289263	NC_037640.1	LG3	13533975	13536871	1.1	3.8E-02
LOC724451	724451	FAD-linked sulfhydryl oxidase ALR, transcript variant X2	NC_037646.1	LG9	7464498	7466365	1.1	3.2E-02
LOC100577293	100577293	uncharacterized	NC_037640.1	LG3	9846619	9848006	1.1	1.1E-02
LOC724275	724275	uncharacterized	NC_037639.1	LG2	3887868	3909867	1.1	1.1E-02
LOC102654610	102654610	putative hydroxypyruvate isomerase	NC_037653.1	LG16	4890774	4892064	1.1	8.9E-03
LOC100577209	100577209	SRR1-like protein	NC_037652.1	LG15	7873561	7875837	1.1	4.4E-02
LOC725086	725086	phosphatidylinositol N-acetylglucosaminyltransferase subunit P, transcript variant X1	NC_037649.1	LG12	10078430	10079493	1.1	3.2E-02
Imd	100302584	immune deficiency	NC_037642.1	LG5	13679797	13682785	1.1	2.5E-02
LOC552410	552410	V-type proton ATPase subunit e 2	NC_037652.1	LG15	7760756	7761556	1.1	4.7E-02
LOC107964262	107964262	uncharacterized	NC_037641.1	LG4	12712850	12715785	1.1	3.7E-02

LOC726872	726872	LDLR chaperone boca	NC_037645.1	LG8	1633684	1634873	1.0	3.8E-03
LOC551125	551125	40S ribosomal protein S15Aa	NC_037640.1	LG3	10171859	10173007	1.0	9.7E-03
LOC551769	551769	grpE protein homolog, mitochondrial	NC_037652.1	LG15	5224824	5226496	1.0	4.5E-02
LOC725308	725308	histone H2A	NC_037638.1	LG1	15879321	15879808	1.0	4.5E-02
LOC413736	413736	serine/threonine-protein kinase SIK2, transcript variant X1	NW_020555815.1	LG10	15727	21538	1.0	2.1E-02
LOC551685	551685	uncharacterized	NC_037653.1	LG16	6609665	6625883	1.0	4.9E-02
LOC100576401	100576401	uncharacterized	NC_037645.1	LG8	8656590	8657361	1.0	9.2E-03
LOC551489	551489	phospholipid phosphatase 5, transcript variant X2	NC_037652.1	LG15	5813088	5814741	1.0	4.6E-02
LOC552745	552745	O-phosphoseryl-tRNA(Sec) selenium transferase, transcript variant X3	NC_037652.1	LG15	3727533	3731000	1.0	2.3E-02
LOC102654261	102654261	uncharacterized	NC_037652.1	LG15	5097034	5098330	1.0	2.7E-02
LOC726757	726757	replication protein A 14 kDa subunit, transcript variant X2	NC_037648.1	LG11	15500131	15501158	1.0	4.1E-02
LOC100576293	100576293	zinc finger protein 182	NC_037644.1	LG7	10976128	10981728	1.0	4.6E-02
LOC411306	411306	inositol hexakisphosphate kinase 2, transcript variant X4	NC_037646.1	LG9	10944049	10960983	1.0	4.9E-02
LOC725688	725688	lipopolysaccharide-induced tumor necrosis factor-alpha factor homolog, transcript variant X1	NC_037648.1	LG11	2830176	2833279	1.0	4.5E-02
LOC113219102	113219102	sex determination protein fruitless-like	NC_037648.1	LG11	4405160	4406812	1.0	4.4E-02
LOC551088	551088	asparagine--tRNA ligase, cytoplasmic	NC_037643.1	LG6	15418838	15421860	1.0	1.0E-03
LOC725733	725733	uncharacterized	NC_037642.1	LG5	9386013	9387328	1.0	4.1E-02
LOC413915	413915	Golgi SNAP receptor complex member 1	NC_037645.1	LG8	1799744	1801638	1.0	3.6E-02
LOC412954	412954	protein KTI12 homolog	NC_037651.1	LG14	8218547	8219831	1.0	4.3E-02
LOC552730	552730	ras-related protein Rab-24	NC_037652.1	LG15	1847455	1849068	1.0	9.6E-03
LOC100578341	100578341	uncharacterized	NC_037647.1	LG10	6453164	6456503	1.0	4.1E-02
LOC102655272	102655272	NHP2-like protein 1	NC_037648.1	LG11	7116133	7117143	1.0	1.5E-02
LOC102655506	102655506	uncharacterized	NC_037643.1	LG6	6860124	6865514	1.0	1.7E-02
LOC727294	727294	protein chibby homolog 1	NC_037651.1	LG14	10431627	10432451	1.0	3.4E-03
LOC409726	409726	adenosine monophosphate-	NC_037642.1	LG5	13757504	13760286	1.0	4.5E-02

		protein transferase Fic						
LOC102654939	102654939	uncharacterized	NC_037639.1	LG2	14725722	14730196	1.0	2.9E-02
LOC725247	725247	probable inactive tRNA-specific adenosine deaminase-like protein 3	NC_037650.1	LG13	3736834	3738075	1.0	4.4E-02
LOC725143	725143	probable U3 small nucleolar RNA-associated protein 11, transcript variant X1	NC_037639.1	LG2	11773003	11775453	1.0	3.9E-02
LOC552138	552138	Mig-2-like GTPase Mtl	NC_037641.1	LG4	5917130	5919072	1.0	3.4E-02
LOC551376	551376	activating signal cointegrator 1 complex subunit 1	NC_037645.1	LG8	11781933	11783978	1.0	2.1E-02
LOC725789	725789	ragulator complex protein LAMTOR1	NC_037639.1	LG2	6758064	6759794	1.0	1.4E-03
LOC726151	726151	probable 39S ribosomal protein L49, mitochondrial	NC_037648.1	LG11	15164643	15166563	1.0	1.9E-02
LOC100576770	100576770	serine hydrolase-like protein 2, transcript variant X2	NC_037638.1	LG1	7968300	7970147	1.0	1.6E-02
LOC102656305	102656305	uncharacterized	NC_037648.1	LG11	1021248	1024890	1.0	4.4E-02
LOC552774	552774	60S ribosomal protein L28, transcript variant X1	NC_037648.1	LG11	4260226	4262644	1.0	4.7E-02
LOC552524	552524	nicotinamidase	NC_037646.1	LG9	11369175	11372013	0.9	2.8E-03
LOC408711	408711	zinc finger matrin-type protein 5, transcript variant X1	NC_037639.1	LG2	5659573	5660873	0.9	1.1E-02
LOC100578035	100578035	sphingolipid delta(4)-desaturase DES1, transcript variant X2	NC_037653.1	LG16	4900449	4902899	0.9	1.9E-03
LOC410612	410612	solute carrier family 25 member 44, transcript variant X1	NC_037652.1	LG15	6403873	6405869	0.9	2.9E-02
LOC726172	726172	death-associated inhibitor of apoptosis 2, transcript variant X2	NC_037638.1	LG1	14808709	14814045	0.9	9.1E-03
LOC551255	551255	mitochondrial fission 1 protein	NC_037639.1	LG2	3021268	3024058	0.9	4.2E-02
LOC551029	551029	inhibitor of growth protein 5	NC_037645.1	LG8	2608026	2609358	0.9	1.5E-03
LOC408573	408573	eukaryotic translation initiation factor 6	NC_037638.1	LG1	20268001	20269587	0.9	4.9E-02
LOC724930	724930	caspase-8	NC_037643.1	LG6	16361827	16364777	0.9	3.6E-02
LOC725811	725811	E3 ubiquitin-protein ligase RNF181, transcript variant X2	NC_037652.1	LG15	1901126	1902717	0.9	2.4E-02

LOC408733	408733	protein pinocchio, transcript variant X1	NC_037640.1	LG3	11707475	11769791	0.9	2.5E-02
LOC724486	724486	uncharacterized	NC_037647.1	LG10	5690526	5692098	0.9	4.6E-02
LOC411077	411077	NEDD8-conjugating enzyme UBE2F	NC_037642.1	LG5	5893824	5895537	0.9	4.7E-02
LOC408672	408672	programmed cell death protein 6, transcript variant X3	NC_037639.1	LG2	14432742	14434626	0.9	4.4E-02
LOC724794	724794	multifunctional methyltransferase subunit TRM112-like protein	NC_037638.1	LG1	25154976	25156342	0.9	7.7E-03
LOC550992	550992	thioredoxin reductase-like selenoprotein T homolog CG3887	NC_037638.1	LG1	25087375	25088764	0.9	4.7E-02
LOC727309	727309	glutaredoxin-C4, transcript variant X2	NC_037651.1	LG14	10573041	10574239	0.9	8.0E-03
LOC726341	726341	ubiquinol-cytochrome-c reductase complex assembly factor 1	NC_037645.1	LG8	11936687	11938408	0.9	1.7E-02
LOC412349	412349	ER membrane protein	NC_037653.1	LG16	5266021	5267111	0.9	3.8E-02
LOC726492	726492	transcription factor MafG	NC_037651.1	LG14	7723471	7724748	0.9	1.6E-02
LOC408713	408713	UPF0235 protein C15orf40 homolog	NC_037639.1	LG2	5556309	5557601	0.9	4.6E-02
LOC410343	410343	nuclear pore complex protein Nup93	NC_037648.1	LG11	13215400	13218668	0.9	3.8E-02
LOC724681	724681	bis(5'-nucleosyl)-tetraphosphatase	NC_037638.1	LG1	14370342	14370991	0.9	3.1E-02
LOC413711	413711	mitochondrial uncoupling protein Bmcp	NC_037642.1	LG5	11423904	11426955	0.9	4.9E-02
LOC102654708	102654708	transcription initiation protein SPT3 homolog, transcript variant X1	NC_037652.1	LG15	1751161	1752770	0.9	4.0E-02
LOC409959	409959	cyclin-dependent kinase 2	NC_037649.1	LG12	1386200	1389107	0.9	4.8E-02
LOC551818	551818	uncharacterized	NC_037638.1	LG1	10786310	10791968	0.9	1.3E-02
LOC726423	726423	transient receptor potential cation channel protein painless, transcript variant X2	NC_037643.1	LG6	13287832	13292359	0.9	3.7E-02
LOC413553	413553	zinc transporter ZIP1-like, transcript variant X1	NC_037649.1	LG12	3102442	3116747	0.8	3.7E-02
LOC408853	408853	X-box-binding protein 1, transcript variant X1	NC_037642.1	LG5	11251428	11253335	0.8	3.4E-02
LOC100577661	100577661	uncharacterized	NC_037639.1	LG2	14643434	14644408	0.8	3.7E-02

LOC409100	409100	ubiquitin-conjugating enzyme E2 W	NC_037638.1	LG1	11835478	11837641	0.8	4.6E-02
LOC726447	726447	histone H2B	NC_037638.1	LG1	15185754	15186376	0.8	2.9E-02
LOC551363	551363	vesicle transport through interaction with t-SNAREs homolog 1A, transcript variant X2	NC_037650.1	LG13	10324191	10326978	0.8	3.6E-02
LOC551133	551133	methyltransferase-like protein 6, transcript variant X2	NC_037646.1	LG9	10066545	10068358	0.8	2.9E-02
LOC727025	727025	DNA-directed RNA polymerases I, II, and III subunit RPABC4	NC_037650.1	LG13	1761527	1763439	0.8	4.7E-02
LOC413261	413261	serine/threonine-protein kinase polo, transcript variant X2	NC_037648.1	LG11	15061994	15066692	0.8	3.9E-02
LOC725363	725363	isopentenyl-diphosphate Delta-isomerase 1, transcript variant X2	NC_037642.1	LG5	10720587	10722797	0.8	4.6E-02
LOC102653879	102653879	homocysteine-responsive endoplasmic reticulum-resident ubiquitin-like domain member 2 protein	NC_037651.1	LG14	6858013	6860073	0.8	1.5E-02
LOC725717	725717	polycomb group RING finger protein 3	NC_037652.1	LG15	1974971	1977654	0.8	4.2E-02
LOC410225	410225	probable E3 ubiquitin-protein ligase RNF144A, transcript variant X1	NC_037647.1	LG10	6385866	6388080	0.8	1.0E-02
LOC726667	726667	armadillo repeat-containing protein 8, transcript variant X1	NC_037638.1	LG1	11738498	11742740	0.8	9.0E-03
LOC725720	725720	muscle, skeletal receptor tyrosine protein kinase, transcript variant X2	NC_037638.1	LG1	4733384	4745332	0.8	2.5E-02
LOC408601	408601	aprataxin	NC_037638.1	LG1	8533202	8534672	0.8	9.3E-03
LOC725740	725740	uncharacterized	NC_037640.1	LG3	1949135	1951308	0.8	5.0E-02
LOC724207	724207	phospholipid scramblase 2, transcript variant X7	NC_037652.1	LG15	2528691	2537737	0.8	4.5E-02
LOC724353	724353	prion-like-(Q/N-rich) domain-bearing protein 25	NC_037644.1	LG7	3250519	3256028	0.7	1.1E-02
LOC551546	551546	BAG family molecular chaperone regulator 2	NC_037638.1	LG1	9069756	9071792	0.7	4.4E-02
Trxr-1	410032	thioredoxin reductase 1	NC_037638.1	LG1	5454493	5460515	0.7	2.2E-02
LOC113218840	113218840	succinate dehydrogenase	NC_037643.1	LG6	2976340	2977497	0.7	3.4E-02

		assembly factor 2, mitochondrial-like						
MsrA	409097	uncharacterized	NC_037638.1	LG1	24242210	24245935	0.7	2.0E-03
LOC102656249	102656249	protein HEXIM1	NC_037642.1	LG5	9504339	9505852	0.7	4.7E-02
Tspan6	406108	tetraspanin 6	NC_037649.1	LG12	7357756	7366851	0.7	4.6E-02
LOC408416	408416	suppressor protein SRP40	NC_037650.1	LG13	4550397	4553118	0.7	1.9E-02
LOC413825	413825	probable ribonuclease ZC3H12C, transcript variant X1	NC_037642.1	LG5	11965679	11975487	0.6	2.5E-02

[a]Gene symbol, gene ID, genomic accession, chromosome, start, and end site per chromosome was based on the Honey bee genome (Amel_HAv3.1; ftp://ftp.ncbi.nlm.nih.gov/genomes/Apis_mellifera: National Center for Biotechnology Information (Bethesda (MD) [24]: National Library of Medicine (US), National Center for Biotechnology Information; [1988] - [cited 2024 Feb 15]). Available from https://www.ncbi.nlm.nih.gov/

[b]Gene annotation based on g:profiler search for biological process terms, considering a depth of two hierarchical levels [30]. ShinyGO v.077 shearch (https://biosnips.org/category/next-generation-sequencing-analysis/ [32] - [cited 2024 Feb 15]).

[c]Log2 fold change was calculated using the DESeq2 R package (Anders & Huber, 2010) and edge R Bioconductor package (Robinson et al., 2010) based on the raw read counts.

Table S4. Characteristics of up-regulated DEGs unique to the LVG-V versus LVG-C comparison (p<0.05, log2FC).

Gene symbol[a]	Gene ID[a]	Gene annotation[b]	Genomic accession[a]	Chromosome[a]	Start[a]	End[a]	log2FC[c]	p-value[c]
KEF36_p10	uncharacterized	uncharacterized	uncharacterized	uncharacterized	uncharacterized	uncharacterized	2.3	0.0003
KEF36_p02	uncharacterized	uncharacterized	uncharacterized	uncharacterized	uncharacterized	uncharacterized	2.2	0.0006
KEF36_p04	uncharacterized	uncharacterized	uncharacterized	uncharacterized	uncharacterized	uncharacterized	2.2	0.0012
LOC113218735	113218735	uncharacterized	NC_037641.1	LG4	1197740	1198580	2.1	0.0011
LOC102656901	102656901	uncharacterized	NC_037642.1	LG5	2972845	2973897	2.1	0.0011
KEF36_r02	uncharacterized	uncharacterized	uncharacterized	uncharacterized	uncharacterized	uncharacterized	2.1	0.0011
LOC113218833	113218833	transmembrane protein 234 homolog	NC_037643.1	LG6	91907	92685	2.1	0.0028
LOC102654920	102654920	COMM domain-containing protein 7	NC_037650.1	LG13	5533935	5534578	2.1	0.0007
LOC102655660	102655660	uncharacterized	NC_037648.1	LG11	13494829	13495849	2.1	0.0036
KEF36_p09	uncharacterized	uncharacterized	uncharacterized	uncharacterized	uncharacterized	uncharacterized	2.1	0.0019
KEF36_p03	uncharacterized	uncharacterized	uncharacterized	uncharacterized	uncharacterized	uncharacterized	2.1	0.0025
LOC113218740	113218740	uncharacterized	NC_037641.1	LG4	4700908	4707745	2.0	0.0042
KEF36_p11	uncharacterized	uncharacterized	uncharacterized	uncharacterized	uncharacterized	uncharacterized	2.0	0.0030
LOC102655126	102655126	uncharacterized	NC_037652.1	LG15	9319034	9323987	2.0	0.0031
LOC102656725	102656725	uncharacterized	NC_037648.1	LG11	13628269	13628864	2.0	0.0029
LOC102654852	102654852	uncharacterized	NC_037653.1	LG16	2237585	2238757	2.0	0.0012
LOC113218630	113218630	up-regulated during skeletal muscle growth protein 5-like	NC_037640.1	LG3	2196974	2197612	1.9	0.0010
LOC102654806	102654806	uncharacterized	NC_037649.1	LG12	5268085	5273816	1.9	0.0064
LOC727186	727186	dolichol-phosphate mannosyltransferase subunit 3, transcript variant X1	NC_037638.1	LG1	9114120	9116094	1.9	0.0048
LOC102654159	102654159	dnaJ homolog subfamily C member 18-like	NC_037638.1	LG1	4486178	4486913	1.9	0.0015
LOC100577644	100577644	uncharacterized	NC_037648.1	LG11	12184320	12185329	1.9	0.0027
LOC726329	726329	venom peptide isomerase heavy chain	NC_037650.1	LG13	5992173	5993569	1.9	0.0031
LOC113219256	113219256	replication protein A 32 kDa subunit-like	NC_037638.1	LG1	4752334	4753718	1.9	0.0033
LOC727142	727142	uncharacterized	NW_020555859.1	uncharacterized	296852	297665	1.9	0.0016

LOC725620	725620	28S ribosomal protein S33, mitochondrial	NC_037651.1	LG14	2452821	2453707	1.8	0.0044
LOC100576918	100576918	uncharacterized	NC_037638.1	LG1	25067896	25068896	1.8	0.0100
LOC102655765	102655765	dnaJ homolog subfamily C member 4	NC_037647.1	LG10	8724475	8725573	1.8	0.0098
Mir1175	100629076	microRNA 1175	NW_020555859.1	uncharacterized	228877	229013	1.8	0.0123
LOC107965704	107965704	uncharacterized	NC_037653.1	LG16	7201213	7202267	1.8	0.0093
LOC113218798	113218798	uncharacterized	NC_037638.1	LG1	14363551	14364107	1.8	0.0113
LOC725675	725675	tetratricopeptide repeat protein 19 homolog, mitochondrial-like	NC_037647.1	LG10	6445359	6446814	1.8	0.0076
LOC113219331	113219331	U1 spliceosomal RNA	NC_037653.1	LG16	6236540	6236701	1.8	0.0187
LOC113218969	113218969	U2 spliceosomal RNA	NC_037645.1	LG8	7179430	7179605	1.8	0.0339
LOC113218755	113218755	small nucleolar RNA U3	NC_037641.1	LG4	5940309	5940523	1.8	0.0170
LOC102656199	102656199	uncharacterized	NC_037642.1	LG5	5897053	5898357	1.8	0.0421
LOC113219265	113219265	trypsin-1-like	NC_037652.1	LG15	7484131	7486821	1.7	0.0072
LOC726750	726750	fibroin heavy chain, transcript variant X5	NC_037646.1	LG9	8492334	8499522	1.7	0.0211
RpLP1	406120	ribosomal protein LP1	NC_037647.1	LG10	10324175	10325596	1.7	0.0049
LOC725147	725147	40S ribosomal protein S29, transcript variant X2	NC_037642.1	LG5	7443754	7444533	1.7	0.0050
LOC725883	725883	guanine nucleotide-binding protein subunit gamma-1	NC_037653.1	LG16	4886828	4887148	1.7	0.0022
LOC725240	725240	mitochondrial import inner membrane translocase subunit Tim9, transcript variant X2	NC_037647.1	LG10	5880997	5881813	1.7	0.0086
LOC113219088	113219088	uncharacterized	NC_037638.1	LG1	8611069	8621754	1.7	0.0162
LOC113219425	113219425	uncharacterized	NC_037639.1	LG2	14197485	14202427	1.7	0.0219
LOC552232	552232	ADP-ribosylation factor-like protein 2-binding protein-like	NC_037648.1	LG11	13214120	13215469	1.7	0.0219
LOC102653960	102653960	magnesium-dependent phosphatase 1	NC_037644.1	LG7	12061518	12062356	1.7	0.0140
LOC102654108	102654108	uncharacterized	NC_037645.1	LG8	3506528	3507575	1.7	0.0085
LOC102656683	102656683	uncharacterized	NC_037648.1	LG11	14208347	14211195	1.7	0.0197
LOC100576662	100576662	uncharacterized	NC_037638.1	LG1	23243910	23246167	1.7	0.0139

LOC113219418	113219418	uncharacterized	NC_037638.1	LG1	10719813	10720840	1.6	0.0223
LOC102655979	102655979	pupal cuticle protein PCP52	NC_037651.1	LG14	9998107	9999544	1.6	0.0345
KEF36_p08	uncharacterized	uncharacterized	uncharacterized	uncharacterized	uncharacterized	uncharacterized	1.6	0.0262
LOC102656758	102656758	uncharacterized	NC_037640.1	LG3	4592682	4593605	1.6	0.0435
LOC107965838	107965838	poly(U)-specific endoribonuclease homolog	NC_037643.1	LG6	1816333	1817470	1.6	0.0376
LOC100576930	100576930	uncharacterized	NC_037640.1	LG3	658765	660058	1.6	0.0059
LOC724654	724654	cytochrome b5	NC_037642.1	LG5	12759076	12760360	1.6	0.0254
LOC725705	725705	mitochondrial import inner membrane translocase subunit Tim13	NC_037642.1	LG5	13106688	13107992	1.6	0.0068
LOC107964792	107964792	coiled-coil-helix-coiled-coil-helix domain-containing protein 7	NC_037645.1	LG8	2294276	2295187	1.6	0.0222
TpnCIIIb	408380	troponin C type IIIb	NC_037649.1	LG12	6991356	6993012	1.6	0.0031
LOC410235	410235	toll-like receptor Tollo	NC_037647.1	LG10	6223652	6228440	1.6	0.0311
KEF36_p13	uncharacterized	uncharacterized	uncharacterized	uncharacterized	uncharacterized	uncharacterized	1.6	0.0277
LOC107965350	107965350	uncharacterized	NC_037649.1	LG12	2330852	2332242	1.6	0.0286
LOC725202	725202	chymotrypsin inhibitor	NC_037652.1	LG15	5673199	5673954	1.6	0.0100
LOC725884	725884	60S acidic ribosomal protein P2	NC_037638.1	LG1	2227209	2228672	1.6	0.0054
LOC725364	725364	flightin	NC_037642.1	LG5	11940771	11942558	1.6	0.0049
LOC726617	726617	39S ribosomal protein L48, mitochondrial, transcript variant X2	NC_037645.1	LG8	3410314	3411983	1.6	0.0297
LOC724507	724507	RIB43A-like with coiled-coils protein 1	NC_037639.1	LG2	11231597	11234197	1.6	0.0345
LOC102655363	102655363	uncharacterized	NC_037653.1	LG16	5265066	5265817	1.6	0.0297
LOC102654158	102654158	keratin, type I cytoskeletal 9-like	NC_037646.1	LG9	10121458	10123011	1.6	0.0078
LOC102655452	102655452	protein bcn92	NC_037641.1	LG4	11964260	11965059	1.6	0.0372
LOC102654398	102654398	uncharacterized	NC_037638.1	LG1	4535965	4537097	1.6	0.0383
CPF2	726691	cuticular protein CPF2	NC_037652.1	LG15	7840678	7842089	1.6	0.0277
LOC413060	413060	DNA-binding protein D-ETS-4, transcript variant X1	NC_037639.1	LG2	6513195	6575110	1.6	0.0123

LOC102654445	102654445	uncharacterized	NC_037639.1	LG2	14835996	14837392	1.6	0.0324
LOC413892	413892	ankyrin repeat domain-containing protein 65, transcript variant X3	NC_037653.1	LG16	797483	814172	1.6	0.0250
LOC100577670	100577670	uncharacterized	NC_037639.1	LG2	10862818	10889458	1.6	0.0260
Mir14	100315661	microRNA 14	NC_037648.1	LG11	2869218	2869317	1.6	0.0421
LOC113219216	113219216	U2 spliceosomal RNA	NC_037650.1	LG13	10290433	10290627	1.6	0.0435
LOC726611	726611	uncharacterized	NC_037647.1	LG10	10971213	10972748	1.5	0.0421
LOC551381	551381	uncharacterized	NC_037638.1	LG1	25138000	25138911	1.5	0.0032
LOC409826	409826	proteoglycan 4, transcript variant X3	NC_037650.1	LG13	4396000	4412453	1.5	0.0497
LOC410627	410627	putative aminopeptidase W07G4.4, transcript variant X2	NC_037652.1	LG15	4673343	4691678	1.5	0.0371
KEF36_p01	uncharacterized	uncharacterized	uncharacterized	uncharacterized	uncharacterized	uncharacterized	1.5	0.0345
Rpl39	724966	ribosomal protein L39	NC_037653.1	LG16	4644650	4645332	1.5	0.0012
LOC100578248	100578248	uncharacterized	NC_037641.1	LG4	8730889	8777332	1.5	0.0183
LOC724489	724489	ubiquitin-like protein 5, transcript variant X1	NC_037640.1	LG3	3235887	3236911	1.5	0.0072
LOC107964283	107964283	uncharacterized	NC_037638.1	LG1	15102852	15103445	1.5	0.0049
LOC726253	726253	UDP-N-acetylglucosamine transferase subunit ALG13 homolog	NC_037641.1	LG4	8316	9144	1.5	0.0280
LOC410322	410322	ral guanine nucleotide dissociation stimulator, transcript variant X3	NC_037648.1	LG11	12052101	12089361	1.5	0.0223
LOC725606	725606	serine protease gd, transcript variant X3	NC_037638.1	LG1	5976954	5981354	1.5	0.0372
LOC102655203	102655203	EKC/KEOPS complex subunit TPRKB	NC_037648.1	LG11	6464449	6465207	1.5	0.0254
Rpl41	100191002	ribosomal protein L41	NC_037648.1	LG11	12183567	12184247	1.5	0.0061
LOC410684	410684	orthodenticle 1, transcript variant X1	NC_037638.1	LG1	20054743	20091403	1.5	0.0371
LOC100576563	100576563	growth factor receptor-bound protein 14, transcript variant X5	NC_037653.1	LG16	996532	1051698	1.5	0.0306
LOC724378	724378	BMP and activin membrane-bound inhibitor homolog	NC_037650.1	LG13	4528727	4550333	1.5	0.0151

LOC113218762	113218762	uncharacterized	NC_037642.1	LG5	13646342	13646928	1.5	0.0340
LOC100578218	100578218	NADH dehydrogenase	NC_037652.1	LG15	7452271	7453184	1.5	0.0001
LOC408308	408308	coiled-coil-helix-coiled-coil-helix domain-containing protein 10, mitochondrial	NC_037648.1	LG11	13528083	13529173	1.5	0.0292
LOC113218850	113218850	alpha-endosulfine, transcript variant X2	NC_037638.1	LG1	22607349	22609163	1.5	0.0000
LOC552387	552387	UDP-galactose transporter senju	NC_037643.1	LG6	13042025	13044786	1.5	0.0049
LOC408443	408443	uncharacterized	NC_037651.1	LG14	4398112	4448290	1.5	0.0220
LOC100578560	100578560	28S ribosomal protein S18c, mitochondrial	NC_037652.1	LG15	2812701	2813409	1.5	0.0121
LOC726297	726297	cytochrome c oxidase subunit 7C, mitochondrial	NC_037645.1	LG8	2288090	2288940	1.5	0.0009
LOC725725	725725	peritrophin-1	NC_037638.1	LG1	26176929	26178009	1.5	0.0451
LOC102656039	102656039	uncharacterized	NC_037652.1	LG15	1624801	1628650	1.5	0.0274
LOC113218928	113218928	uncharacterized	NC_037645.1	LG8	7644263	7645581	1.4	0.0484
LOC100578890	100578890	uncharacterized	NC_037647.1	LG10	7719953	7723191	1.4	0.0274
LOC100576983	100576983	uncharacterized	NC_037651.1	LG14	6830470	6831866	1.4	0.0312
LOC100578051	100578051	uncharacterized	NC_037645.1	LG8	3270026	3312864	1.4	0.0443
LOC725352	725352	U6 snRNA-associated Sm-like protein LSm7, transcript variant X2	NC_037648.1	LG11	15935398	15936256	1.4	0.0254
LOC102656183	102656183	histidine triad nucleotide-binding protein 1	NC_037650.1	LG13	3795324	3796441	1.4	0.0222
LOC113219175	113219175	U6 spliceosomal RNA	NC_037649.1	LG12	3959066	3959172	1.4	0.0467
LOC551680	551680	histamine-gated chloride channel 2, transcript variant X2	NC_037638.1	LG1	19137308	19190701	1.4	0.0416
LOC409861	409861	nucleoside diphosphate kinase	NC_037648.1	LG11	2833958	2835333	1.4	0.0249
LOC100576985	100576985	uncharacterized	NC_037648.1	LG11	15343862	15348119	1.4	0.0345
LOC100577997	100577997	CAPA peptides, transcript variant X1	NC_037644.1	LG7	2216026	2224274	1.4	0.0432
LOC408669	408669	uncharacterized	NC_037639.1	LG2	13880761	13888198	1.4	0.0435
LOC726599	726599	angiotensin-converting enzyme	NC_037639.1	LG2	13880761	13888198	1.4	0.0125
LOC100578713	100578713	uncharacterized	NC_037646.1	LG9	5814471	5815148	1.4	0.0203

LOC410486	410486	40S ribosomal protein SA	NC_037651.1	LG14	5111502	5113767	1.4	0.0493
LOC113218578	113218578	uncharacterized	NC_037638.1	LG1	4730496	4732211	1.4	0.0100
LOC107963993	107963993	palmitoleoyl-protein carboxylesterase NOTUM	NC_037638.1	LG1	23774646	23798445	1.4	0.0257
LOC100576195	100576195	protein ccsmst1, transcript variant X5	NC_037651.1	LG14	8117950	8118839	1.4	0.0109
LOC551304	551304	selenoprotein K-like	NC_037644.1	LG7	4856099	4856902	1.4	0.0022
LOC411832	411832	translationally-controlled tumor protein homolog	NC_037646.1	LG9	7398474	7400714	1.4	0.0131
LOC552564	552564	40S ribosomal protein S7	NC_037653.1	LG16	5208227	5209861	1.4	0.0093
LOC408782	408782	tubulin beta-1	NC_037641.1	LG4	12053300	12058225	1.4	0.0432
Cox6c	100359410	cytochrome c oxidase subunit VIc	NC_037643.1	LG6	1851373	1852552	1.4	0.0015
LOC113218594	113218594	uncharacterized	NC_037639.1	LG2	15090980	15093593	1.4	0.0435
LOC100578782	100578782	cytochrome b-c1 complex subunit 9, transcript variant X1	NC_037638.1	LG1	14347351	14348563	1.3	0.0405
LOC102653609	102653609	paramyosin, short form-like	NC_037643.1	LG6	4492820	4496800	1.3	0.0389
LOC107965891	107965891	electron transfer flavoprotein regulatory factor 1	NC_037638.1	LG1	5034680	5035325	1.3	0.0362
LOC411515	411515	60S ribosomal protein L44	NC_037641.1	LG4	11115161	11116216	1.3	0.0011
LOC100578113	100578113	protein CUSTOS	NC_037645.1	LG8	514607	515546	1.3	0.0361
LOC102655440	102655440	uncharacterized	NC_037648.1	LG11	6405210	6408724	1.3	0.0092
LOC102655756	102655756	PDZ and LIM domain protein 7-like	NC_037647.1	LG10	10557516	10559091	1.3	0.0468
LOC726236	726236	neither inactivation nor afterpotential protein G-like	NC_037643.1	LG6	1977177	1982816	1.3	0.0366
LOC113218756	113218756	U11 spliceosomal RNA	NC_037641.1	LG4	11518215	11518352	1.3	0.0073
Ant	406075	ADP/ATP translocase	NC_037644.1	LG7	10937513	10940303	1.3	0.0438
LOC725482	725482	L-lactate dehydrogenase A-like 6A, transcript variant X3	NC_037644.1	LG7	13821623	13941601	1.3	0.0448
LOC413014	413014	NADH dehydrogenase	NC_037642.1	LG5	13725195	13726100	1.3	0.0038
LOC107966045	107966045	uncharacterized	NC_037638.1	LG1	23251314	23256231	1.3	0.0131
LOC107965237	107965237	flocculation protein FLO11	NC_037648.1	LG11	1498019	1502769	1.3	0.0424

LOC102656529	102656529	uncharacterized	NC_037645.1	LG8	1817662	1820523	1.3	0.0269
LOC100578606	100578606	uncharacterized	NC_037638.1	LG1	25828074	25833879	1.2	0.0256
LOC726247	726247	protein vestigial, transcript variant X1	NC_037649.1	LG12	11405644	11466368	1.2	0.0087
LOC107964197	107964197	uncharacterized	NC_037640.1	LG3	4609536	4611283	1.2	0.0297
LOC725266	725266	oligosaccharyltransferase complex subunit ostc-A	NC_037641.1	LG4	5864597	5866495	1.2	0.0117
LOC724570	724570	mpv17-like protein 2, transcript variant X2	NC_037645.1	LG8	2617571	2618970	1.2	0.0448
Ndufs5	727061	NADH dehydrogenase (ubiquinone) Fe-S protein 5, 15kDa (NADH-coenzyme Q reductase)	NC_037642.1	LG5	13858451	13859322	1.2	0.0181
LOC727280	727280	uncharacterized	NC_037651.1	LG14	10469677	10474098	1.2	0.0062
LOC408909	408909	NADH-quinone oxidoreductase subunit B 2, transcript variant X2	NC_037644.1	LG7	13626835	13628255	1.2	0.0495
LOC409586	409586	NADH dehydrogenase	NC_037646.1	LG9	1291780	1292971	1.2	0.0006
LOC724233	724233	40S ribosomal protein S17	NC_037638.1	LG1	4375803	4376815	1.2	0.0340
LOC100578734	100578734	uncharacterized	NC_037640.1	LG3	1957912	1959339	1.2	0.0008
LOC413186	413186	mitochondrial pyruvate carrier 3	NC_037639.1	LG2	10522063	10524943	1.2	0.0083
LOC727599	727599	NADH dehydrogenase	NC_037646.1	LG9	1647665	1648662	1.2	0.0012
Apamin	406135	apamin protein	NC_037649.1	LG12	6649292	6649822	1.2	0.0341
LOC724639	724639	COP9 signalosome complex subunit 9	NC_037638.1	LG1	24153019	24153583	1.1	0.0376
LOC552639	552639	acylphosphatase-1	NC_037647.1	LG10	8132565	8133613	1.1	0.0012
LOC726786	726786	proton-coupled amino acid transporter 1, transcript variant X1	NC_037644.1	LG7	4881425	4932463	1.1	0.0463
LOC724904	724904	acylphosphatase-2, transcript variant X1	NC_037638.1	LG1	18424790	18429288	1.1	0.0131
LOC107966055	107966055	PRKR-interacting protein 1 homolog	NC_037638.1	LG1	4561509	4562876	1.1	0.0351
LOC113219277	113219277	uncharacterized	NC_037652.1	LG15	2826690	2829006	1.1	0.0211
LOC113218601	113218601	uncharacterized	NC_037639.1	LG2	9954462	9962804	1.1	0.0421
LOC552494	552494	60S ribosomal protein L37	NC_037645.1	LG8	2616124	2617451	1.1	0.0340
LOC552261	552261	EF-hand domain-containing protein D2 homolog	NC_037651.1	LG14	5450495	5465164	1.1	0.0131

LOC551106	551106	ubiquitin-fold modifier-conjugating enzyme 1	NC_037653.1	LG16	4911364	4912344	1.1	0.0138
LOC727026	727026	cytochrome c oxidase copper chaperone	NC_037651.1	LG14	9431901	9432765	1.1	0.0222
LOC551232	551232	probable nuclear hormone receptor HR38	NC_037650.1	LG13	5711443	5719422	1.0	0.0492
LOC724315	724315	forkhead box protein D3	NC_037646.1	LG9	6043644	6046657	1.0	0.0074
LOC552237	552237	transmembrane protein 242	NC_037652.1	LG15	9358141	9359190	1.0	0.0015
LOC100578551	100578551	39S ribosomal protein L34, mitochondrial	NC_037638.1	LG1	22776969	22777477	1.0	0.0140
LOC409088	409088	uncharacterized	NC_037639.1	LG2	15258712	15260435	1.0	0.0159
LOC724827	724827	NADH dehydrogenase	NC_037653.1	LG16	2558984	2560114	0.9	0.0500
LOC410043	410043	ornithine decarboxylase, transcript variant X1	NC_037638.1	LG1	5231520	5238005	0.9	0.0043
LOC412309	412309	ras-related C3 botulinum toxin substrate 1	NC_037649.1	LG12	7827912	7829831	0.9	0.0098
LOC724264	724264	NADH dehydrogenase	NC_037645.1	LG8	528143	529121	0.9	0.0435
LOC726546	726546	gem-associated protein 7, transcript variant X1	NC_037647.1	LG10	10944540	10953014	0.9	0.0371
LOC724802	724802	protein Asterix	NC_037649.1	LG12	9968092	9968842	0.9	0.0319
LOC408589	408589	macro domain-containing protein CT2219, transcript variant X1	NC_037638.1	LG1	14427151	14428774	0.8	0.0421
LOC725401	725401	U6 snRNA-associated Sm-like protein LSm3	NC_037640.1	LG3	11988212	11989241	0.8	0.0220
LOC552741	552741	26S proteasome complex subunit SEM1	NC_037642.1	LG5	13161242	13162239	0.8	0.0131
LOC409396	409396	eukaryotic translation initiation factor 4E-binding protein Mextli, transcript variant X3	NC_037648.1	LG11	12204018	12210058	0.8	0.0015
LOC726369	726369	uncharacterized	NC_037638.1	LG1	15173749	15174773	0.8	0.0470
LOC552511	552511	GTP:AMP phosphotransferase AK3, mitochondrial	NC_037648.1	LG11	14053071	14055264	0.7	0.0324
LOC551582	551582	maternal protein exuperantia, transcript variant X6	NC_037651.1	LG14	3453993	3463078	0.7	0.0211
LOC409130	409130	iron-sulfur cluster assembly enzyme ISCU, mitochondrial	NC_037640.1	LG3	4386135	4392013	0.7	0.0328

[a]Gene symbol, gene ID, genomic accession, chromosome, start, and end site per chromosome was based on the Honey bee genome (Amel_HAv3.1; ftp://ftp.ncbi.nlm.nih.gov/genomes/Apis_mellifera: National Center for Biotechnology Information (Bethesda (MD) [24]: National Library of Medicine (US), National Center for Biotechnology Information; [1988] - [cited 2024 Feb 15]). Available from https://www.ncbi.nlm.nih.gov/

[b]Gene annotation based on g:profiler search for biological process terms, considering a depth of two hierarchical levels [30]. ShinyGO v.077 shearch (https://biosnips.org/category/next-generation-sequencing-analysis/ [32] - [cited 2024 Feb 15]).

[c]Log2 fold change was calculated using the DESeq2 R package (Anders & Huber, 2010) and edge R Bioconductor package (Robinson et al., 2010) based on the raw read counts.

Table S5. Characteristics of up-regulated DEGs shared between LVG-V versus LVG-C and HVG-V versus HVG-C comparisons ($p<0.05$, log2FC).

Gene symbol[a]	Gene ID[a]	Gene annotation[b]	Genomic accession[a]	Chromosome[a]	Start[a]	End[a]	log2FC[c]	p-value[c]
LOC113219057	113219057	uncharacterized	NC_037647.1	LG10	2893565	2898392	3.8	6.32E-09
LOC102655788	102655788	uncharacterized	NC_037642.1	LG5	2358563	2360577	2.9	1.3E-09
Obp9	677675	odorant binding protein 9	NC_037652.1	LG15	6325010	6335391	2.9	8.48E-07
LOC113219089	113219089	uncharacterized	NC_037638.1	LG1	8645147	8650832	2.7	5.37E-07
LOC107963975	107963975	uncharacterized	NC_037638.1	LG1	13283980	13285235	2.7	3.3E-06
Nrx-1	724217	neurexin 1	NC_037642.1	LG5	2349284	2687281	2.6	1.57E-05
LOC102655393	102655393	uncharacterized	NC_037643.1	LG6	9696323	9699248	2.6	2.38E-05
LOC102656472	102656472	uncharacterized	NC_037650.1	LG13	7268441	7269899	2.6	1.95E-05
LOC113219065	113219065	uncharacterized	NC_037648.1	LG11	14244593	14245126	2.6	0.000105
LOC113219110	113219110	uncharacterized	NC_037648.1	LG11	13792112	13795420	2.5	6.57E-05
LOC102654944	102654944	uncharacterized	NC_037645.1	LG8	7132398	7133453	2.5	0.000154
LOC113219212	113219212	uncharacterized	NC_037650.1	LG13	8511075	8514461	2.5	1.11E-05
LOC113219227	113219227	uncharacterized	NC_037651.1	LG14	3442392	3443586	2.4	3.79E-05
LOC102656182	102656182	uncharacterized	NC_037647.1	LG10	954863	960217	2.4	0.000299
LOC102656747	102656747	uncharacterized	NC_037638.1	LG1	7992741	7993882	2.4	0.000471
Mir87-1	100315671	microRNA 87-1	NC_037650.1	LG13	9609758	9609857	2.4	0.000185
LOC102654687	102654687	uncharacterized	NC_037640.1	LG3	2786686	2793178	2.4	0.000192
LOC102655202	102655202	uncharacterized	NC_037638.1	LG1	27110304	27113141	2.3	0.000202
LOC102656630	102656630	uncharacterized	NC_037639.1	LG2	10986767	10989283	2.3	0.000162
LOC100578881	100578881	histone H3.3-like type 1	NC_037638.1	LG1	15189784	15190552	2.3	0.001281
LOC102654086	102654086	uncharacterized	NC_037647.1	LG10	7812313	7813973	2.2	0.000331
Mir87-2	100315688	microRNA 87-2	NC_037650.1	LG13	9610356	9610455	2.2	0.000705
LOC113219104	113219104	uncharacterized	NC_037648.1	LG11	10154611	10157975	2.2	0.000361
LOC107965086	107965086	uncharacterized	NC_037647.1	LG10	3046486	3058669	2.1	0.002084
LOC107963977	107963977	uncharacterized	NC_037643.1	LG6	15703232	15706095	2.1	0.000802
LOC113218591	113218591	uncharacterized	NC_037639.1	LG2	5641383	5643742	2.1	0.002208
LOC113219090	113219090	uncharacterized	NC_037638.1	LG1	8652397	8654843	2.1	0.00024
LOC408690	408690	calmodulin, transcript	NC_037639.1	LG2	11290917	11340288	2.0	0.000621

		variant X2						
LOC100578156	100578156	uncharacterized	NC_037642.1	LG5	9818116	9820404	1.9	0.000155
LOC113218531	113218531	uncharacterized	NC_037652.1	LG15	6335705	6338323	1.9	0.008761
LOC113218888	113218888	uncharacterized	NC_037644.1	LG7	8606924	8612101	1.9	0.002946
LOC107965194	107965194	uncharacterized	NC_037648.1	LG11	13692411	13806653	1.8	0.006543
LOC725233	725233	uncharacterized	NC_037644.1	LG7	10246853	10264697	1.8	0.008508
LOC113219189	113219189	uncharacterized	NC_037638.1	LG1	21549963	21562535	1.7	0.000171
LOC726980	726980	uncharacterized	NC_037641.1	LG4	3482600	3485558	1.7	0.002929
LOC102655790	102655790	uncharacterized	NC_037645.1	LG8	1292983	1296360	1.7	0.011084
LOC411599	411599	uridine phosphorylase 1, transcript variant X2	NC_037644.1	LG7	7967174	7995245	1.7	0.000104
LOC724644	724644	uncharacterized	NC_037650.1	LG13	7348468	7354299	1.6	0.007618
LOC113218766	113218766	uncharacterized	NC_037642.1	LG5	9635788	9659409	1.6	0.027672
LOC100577670	100577670	uncharacterized	NC_037639.1	LG2	10862818	10889458	1.6	0.012789
LOC411290	411290	uncharacterized	NC_037645.1	LG8	12226610	12243273	1.6	0.000841
LOC102655495	102655495	uncharacterized	NC_037642.1	LG5	2118743	2120822	1.6	0.001142
LOC100576697	100576697	uncharacterized	NC_037638.1	LG1	23954915	23963214	1.6	0.02468
LOC100578614	100578614	tctex1 domain-containing protein 1	NC_037639.1	LG2	15275481	15277224	1.5	0.037938
LOC102656212	102656212	uncharacterized	NC_037650.1	LG13	6025185	6029256	1.5	0.023387
LOC100578741	100578741	uncharacterized	NC_037642.1	LG5	5642614	5654048	1.5	0.016868
LOC102654388	102654388	uncharacterized	NC_037647.1	LG10	10732100	10743828	1.5	0.001892
LOC100578697	100578697	THUMP domain-containing protein 1 homolog	NC_037648.1	LG11	2501743	2503162	1.5	0.000705
LOC113218819	113218819	uncharacterized	NC_037638.1	LG1	5025266	5027986	1.5	0.0329
LOC102655332	102655332	protein orai-2	NC_037644.1	LG7	2305115	2307862	1.5	0.034554
LOC552797	552797	ETS-related transcription factor Elf-5, transcript variant X3	NC_037642.1	LG5	13441594	13446794	1.4	0.01781
LOC107965085	107965085	bladder cancer-associated protein	NC_037647.1	LG10	9393908	9395137	1.4	4.79E-06
LOC724988	724988	uncharacterized	NC_037649.1	LG12	3958218	3959211	1.4	0.009699
LOC413596	413596	receptor-type guanylate cyclase gcy-	NC_037650.1	LG13	7252933	7281087	1.4	0.017816

		4, transcript variant X3							
LOC102654832	102654832	uncharacterized	NC_037645.1	LG8	9455760	9458006	1.4	0.028872
LOC100577527	100577527	uncharacterized	NC_037641.1	LG4	7609057	7610025	1.4	0.032245
LOC408734	408734	succinate dehydrogenase	NC_037640.1	LG3	11689358	11698726	1.4	0.018328
LOC100578684	100578684	uncharacterized	NC_037653.1	LG16	754969	755601	1.4	0.003832
LOC409159	409159	G-protein coupled receptor moody, transcript variant X1	NC_037647.1	LG10	5949179	5957683	1.3	0.027741
LOC100579019	100579019	probable salivary secreted peptide	NC_037639.1	LG2	9021248	9021916	1.3	0.045629
LOC100577966	100577966	probable serine/threonine-protein kinase DDB_G0283337	NC_037653.1	LG16	5213939	5215773	1.3	0.008427
LOC102655585	102655585	uncharacterized	NC_037652.1	LG15	7556156	7557042	1.3	0.016868
LOC408844	408844	dual specificity protein phosphatase 10, transcript variant X2	NC_037642.1	LG5	10350056	10438356	1.3	0.029483
LOC550703	550703	dephospho-CoA kinase, transcript variant X2	NC_037643.1	LG6	16341846	16343720	1.2	3.27E-05
LOC113218907	113218907	uncharacterized	NC_037644.1	LG7	13797933	13799988	1.2	0.008908
LOC724169	724169	transcription factor kayak, transcript variant X2	NC_037642.1	LG5	6834234	6865775	1.2	0.041259
LOC107964437	107964437	THAP domain-containing protein 4	NC_037642.1	LG5	10937585	10938522	1.2	0.003044
LOC724869	724869	histone H2B	NC_037644.1	LG7	12507716	12508235	1.2	0.003593
LOC100576975	100576975	zinc finger protein 567	NC_037645.1	LG8	11724680	11726340	1.2	0.031627
LOC113218535	113218535	uncharacterized	NC_037650.1	LG13	3007739	3014728	1.2	0.03699
LOC409862	409862	serine/arginine-rich splicing factor 2, transcript variant X2	NC_037651.1	LG14	8139745	8143947	1.2	0.040382
LOC726160	726160	uncharacterized	NC_037642.1	LG5	9534990	9535651	1.2	0.009716
LOC408406	408406	cyclin-dependent kinase 4, transcript variant X4	NC_037650.1	LG13	3775097	3795441	1.2	0.048264
LOC100579038	100579038	succinate dehydrogenase	NC_037650.1	LG13	4336241	4337034	1.2	0.01781

		assembly factor 3, mitochondrial, transcript variant X2						
LOC113218960	113218960	uncharacterized	NC_037638.1	LG1	23221423	23223657	1.2	0.024228
LOC724954	724954	rhodanese domain-containing protein CG4456, transcript variant X3	NC_037648.1	LG11	9834094	9835033	1.2	0.016158
LOC551479	551479	40S ribosomal protein S21	NC_037640.1	LG3	6088392	6089063	1.1	0.024228
LOC727483	727483	protein stunted, transcript variant X1	NC_037641.1	LG4	5505958	5508205	1.1	0.022233
LOC100576945	100576945	uncharacterized	NC_037647.1	LG10	8701417	8702127	1.1	0.041258
LOC102654682	102654682	transcription factor A, mitochondrial	NC_037640.1	LG3	655918	657361	1.1	0.037738
LOC725580	725580	protein SREK1IP1	NC_037648.1	LG11	1074018	1075027	1.1	0.01159
LOC113218567	113218567	CCR4-NOT transcription complex subunit 6-like, transcript variant X3	NC_037644.1	LG7	7130504	7502881	1.1	0.014468
LOC725142	725142	U6 snRNA-associated Sm-like protein LSm2	NC_037647.1	LG10	7740198	7741015	1.1	0.009769
LOC552616	552616	iron-sulfur cluster assembly 1 homolog, mitochondrial	NC_037652.1	LG15	9294635	9296069	1.1	0.001281
LOC552213	552213	activated RNA polymerase II transcriptional coactivator p15, transcript variant X1	NC_037641.1	LG4	12748854	12750057	1.1	0.011283
LOC102655388	102655388	small VCP/p97-interacting protein	NC_037639.1	LG2	4517169	4519104	1.1	0.032592
LOC100577651	100577651	three prime repair exonuclease 2, transcript variant X3	NC_037648.1	LG11	384874	386256	1.1	0.001933
LOC552260	552260	U6 snRNA-associated Sm-like protein LSm1	NC_037638.1	LG1	25307338	25308652	1.1	0.025846
LOC725854	725854	39S ribosomal protein L37, mitochondrial	NC_037647.1	LG10	10306252	10308867	1.0	0.000233
LOC102654345	102654345	uncharacterized	NC_037641.1	LG4	5911779	5912907	1.0	0.035775
LOC724413	724413	uncharacterized	NC_037638.1	LG1	19352806	19353792	1.0	0.028385
LOC552116	552116	histone H3.3	NC_037651.1	LG14	6525913	6527925	1.0	0.006499

LOC550827	550827	tubulin alpha-1 chain	NC_037643.1	LG6	13020093	13022252	1.0	0.036787
LOC408981	408981	activating transcription factor 3, transcript variant X1	NC_037646.1	LG9	11777130	11799422	1.0	0.021592
LOC413378	413378	NTF2-related export protein, transcript variant X2	NC_037638.1	LG1	24133647	24136437	1.0	0.010691
LOC410017	410017	protein OPI10 homolog	NC_037645.1	LG8	2796401	2798235	1.0	0.047073
LOC724241	724241	N-alpha-acetyltransferase 38-B, NatC auxiliary subunit	NC_037647.1	LG10	7068054	7068998	1.0	0.006805
LOC412266	412266	60S ribosomal protein L27, transcript variant X1	NC_037640.1	LG3	10256265	10257980	1.0	0.043899
LOC724346	724346	protein cornichon homolog 4	NC_037641.1	LG4	11502352	11503912	0.9	0.011935
LOC100576542	100576542	protein POLR1D, transcript variant X1	NC_037651.1	LG14	5217975	5218943	0.9	0.041925
LOC724953	724953	U6 snRNA phosphodiesterase, transcript variant X1	NC_037648.1	LG11	8072259	8073934	0.9	0.025846
LOC726527	726527	protein FAM136A	NC_037651.1	LG14	9073614	9078475	0.9	0.008908
Def2	413397	defensin 2	NC_037653.1	LG16	5139659	5140578	0.9	0.024228
LOC724365	724365	ragulator complex protein LAMTOR4 homolog, transcript variant X1	NC_037651.1	LG14	6753886	6755042	0.9	0.023381
LOC724868	724868	40S ribosomal protein S11	NC_037642.1	LG5	5473930	5475282	0.8	0.005882
LOC726653	726653	mitochondrial import inner membrane translocase subunit TIM14, transcript variant X3	NC_037642.1	LG5	6596910	6598694	0.8	0.043973
Uqcr11	726777	ubiquinol-cytochrome c reductase, complex III subunit XI	NC_037638.1	LG1	20626751	20627520	0.8	0.029358
LOC410949	410949	MOB kinase activator-like 4, transcript variant X2	NC_037640.1	LG3	6075718	6078966	0.7	0.016868
LOC100578725	100578725	uncharacterized	NC_037652.1	LG15	1854621	1855852	0.7	0.038262
TRNAA-GGC	107964693	uncharacterized	NC_037643.1	LG6	13661573	13661644	0.5	0.023347

[a]Gene symbol, gene ID, genomic accession, chromosome, start, and end site per chromosome was based on the Honey bee genome (Amel_HAv3.1; ftp://ftp.ncbi.nlm.nih.gov/genomes/Apis_mellifera: National Center for Biotechnology Information (Bethesda (MD) [24]: National Library of Medicine (US), National Center for Biotechnology Information; [1988] - [cited 2024 Feb 15]). Available from https://www.ncbi.nlm.nih.gov/
[b]Gene annotation based on g:profiler search for biological process terms, considering a depth of two hierarchical levels [30]. ShinyGO v.077 shearch (https://biosnips.org/category/next-generation-sequencing-analysis/ [32] - [cited 2024 Feb 15]).
[c]Log2 fold change was calculated using the DESeq2 R package (Anders & Huber, 2010) and edge R Bioconductor package (Robinson et al., 2010) based on the raw read counts.

Table S6. Characteristics of up-regulated DEGs unique to the LVG-V versus HVG-V comparison ($p<0.05$, log2FC).

Gene symbol[a]	Gene ID[a]	Gene annotation[b]	Genomic accession[a]	Chromosome[a]	Start[a]	End[a]	log2FC[c]	p-value[c]
Obp1	406102	odorant binding protein 1	NC_037639.1	LG2	14654212	14655874	2.8	3.8E-10
LOC102654393	102654393	major royal jelly protein 2-like	NC_037648.1	LG11	2297915	2298564	2.3	2.9E-05
LOC107964896	107964896	uncharacterized	NC_037645.1	LG8	12378161	12378908	1.9	1.9E-02
LOC726446	726446	uncharacterized	NC_037638.1	LG1	367913	369386	1.8	4.5E-03
LOC413471	413471	sodium/potassium-transporting ATPase subunit alpha-B	NC_037638.1	LG1	9268131	9271607	1.7	2.9E-02
Obp2	406103	odorant binding protein 2	NC_037646.1	LG9	11974257	11975390	1.5	8.8E-04
LOC551437	551437	alpha-N-acetylglucosaminidase	NC_037644.1	LG7	2082573	2089958	1.5	2.9E-02
LOC406145	406145	secapin	NC_037642.1	LG5	2354864	2356029	1.4	1.7E-02
LOC724429	724429	synaptic vesicle glycoprotein 2C-like	NC_037641.1	LG4	7961856	7970291	1.4	8.7E-04
LOC725546	725546	DDB1- and CUL4-associated factor 6, transcript variant X2	NC_037641.1	LG4	5232795	5240940	0.9	7.4E-03

[a]Gene symbol, gene ID, genomic accession, chromosome, start, and end site per chromosome was based on the Honey bee genome (Amel_HAv3.1; ftp://ftp.ncbi.nlm.nih.gov/genomes/Apis_mellifera: National Center for Biotechnology Information (Bethesda (MD) [24]: National Library of Medicine (US), National Center for Biotechnology Information; [1988] - [cited 2024 Feb 15]). Available from https://www.ncbi.nlm.nih.gov/

[b]Gene annotation based on g:profiler search for biological process terms, considering a depth of two hierarchical levels [30]. ShinyGO v.077 shearch (https://biosnips.org/category/next-generation-sequencing-analysis/ [32] - [cited 2024 Feb 15]).

[c]Log2 fold change was calculated using the DESeq2 R package (Anders & Huber, 2010) and edge R Bioconductor package (Robinson et al., 2010) based on the raw read counts.

Table S7. Characteristics of down-regulated DEGs unique from HVG-V versus HVG-C comparison (p<0.05, log2FC).

Gene symbol[a]	Gene ID[a]	Gene annotation[b]	Genomic accession[a]	Chromosome[a]	Start[a]	End[a]	log2FC[c]	p-value[c]
LOC550798	550798	protein ERGIC-53	NC_037650.1	LG13	10326999	10330830	-0.5	3.0E-02
LOC409196	409196	alanine aminotransferase 1	NC_037645.1	LG8	2153796	2160013	-0.5	4.9E-02
LOC411965	411965	transcription initiation factor IIB, transcript variant X1	NC_037638.1	LG1	11487447	11489550	-0.5	4.9E-02
LOC408840	408840	5'-3' exoribonuclease 2 homolog, transcript variant X2	NC_037642.1	LG5	9722200	9750791	-0.6	3.1E-02
LOC724126	724126	uncharacterized	NC_037653.1	LG16	1891294	1895276	-0.6	3.4E-02
LOC413488	413488	protein vav, transcript variant X3	NC_037640.1	LG3	1971624	1976565	-0.6	4.2E-02
LOC100578888	100578888	zinc finger protein 808	NC_037640.1	LG3	8635100	8642941	-0.6	4.1E-02
Dl	406086	dorsal	NC_037641.1	LG4	11095375	11108126	-0.6	3.9E-02
LOC100577578	100577578	uncharacterized	NC_037648.1	LG11	6455573	6462167	-0.6	4.3E-02
LOC100577455	100577455	golgin subfamily A member 6-like protein 22	NC_037648.1	LG11	7984119	8042314	-0.6	4.7E-02
LOC410936	410936	cytochrome b5 reductase 4, transcript variant X6	NC_037640.1	LG3	7747688	7767312	-0.6	5.0E-02
LOC409452	409452	maltase A2, transcript variant X1	NC_037643.1	LG6	6595121	6607651	-0.6	2.6E-02
LOC550886	550886	atlastin	NC_037642.1	LG5	1016188	1033088	-0.6	2.0E-02
LOC411163	411163	N-alpha-acetyltransferase 15, NatA auxiliary subunit	NC_037643.1	LG6	9673802	9719257	-0.6	4.4E-02
LOC412742	412742	chromatin-remodeling complex ATPase chain Iswi	NC_037649.1	LG12	8034460	8038308	-0.6	4.2E-02
LOC100578209	100578209	BTB/POZ domain-containing protein 6-B, transcript variant X2	NC_037649.1	LG12	8602428	8607514	-0.6	4.3E-02
LOC551519	551519	uncharacterized	NC_037642.1	LG5	244539	252608	-0.6	4.5E-02
LOC100578909	100578909	procollagen-lysine,2-oxoglutarate 5-dioxygenase 3, transcript variant X2	NC_037645.1	LG8	8610050	8619263	-0.6	4.2E-02
LOC726218	726218	acetyl-CoA acetyltransferase, mitochondrial	NC_037638.1	LG1	14801881	14805100	-0.6	3.3E-02
LOC409695	409695	uncharacterized	NC_037648.1	LG11	8782410	8798001	-0.7	4.7E-02
LOC552734	552734	poly(U)-specific endoribonuclease homolog	NC_037647.1	LG10	8199344	8203674	-0.7	9.3E-03
LOC409513	409513	CD9 antigen, transcript variant X1	NC_037652.1	LG15	2689287	2698457	-0.7	3.1E-02
LOC409764	409764	uncharacterized	NC_037648.1	LG11	15936375	15945623	-0.7	4.8E-02

LOC413462	413462	upstream activation factor subunit spp27	NC_037645.1	LG8	7218938	7224053	-0.7	7.7E-03
LOC409077	409077	uncharacterized	NC_037644.1	LG7	7814601	7825138	-0.7	4.7E-02
LOC408793	408793	histone acetyltransferase KAT7, transcript variant X1	NC_037641.1	LG4	11145328	11168624	-0.7	2.8E-02
LOC100578389	100578389	uncharacterized	NC_037638.1	LG1	13215323	13282394	-0.7	1.2E-02
LOC551113	551113	delta-1-pyrroline-5-carboxylate dehydrogenase, mitochondrial	NC_037648.1	LG11	11040682	11046104	-0.7	3.6E-02
LOC409192	409192	probable phospholipid-transporting ATPase VD, transcript variant X1	NC_037639.1	LG2	15049440	15067954	-0.7	4.7E-02
LOC551576	551576	exocyst complex component 7, transcript variant X2	NC_037646.1	LG9	1576390	1583183	-0.7	8.4E-03
LOC413829	413829	diuretic hormone receptor, transcript variant X3	NC_037640.1	LG3	10053870	10140657	-0.7	2.7E-02
LOC412840	412840	homeobox protein caupolican, transcript variant X1	NC_037648.1	LG11	1708582	1798575	-0.7	3.7E-02
LOC100577879	100577879	carbonic anhydrase-related protein 10, transcript variant X2	NC_037648.1	LG11	10767329	10947681	-0.7	4.5E-02
Syt20	410052	synaptotagmin 20	NC_037653.1	LG16	1767494	1778077	-0.7	2.2E-02
LOC724460	724460	uncharacterized	NC_037648.1	LG11	14521083	14562543	-0.7	4.0E-02
LOC410862	410862	solute carrier family 41 member 3, transcript variant X1	NC_037639.1	LG2	11582377	11590912	-0.7	4.5E-02
LOC409892	409892	dnaJ homolog subfamily C member 9, transcript variant X1	NC_037649.1	LG12	2791512	2817057	-0.7	2.2E-02
LOC409963	409963	6-phosphofructo-2-kinase/fructose-2,6-bisphosphatase, transcript variant X2	NC_037650.1	LG13	4833036	4843322	-0.7	1.6E-02
LOC413411	413411	myotubularin-related protein 14	NC_037644.1	LG7	1567936	1571206	-0.7	4.5E-02
LOC100576569	100576569	uncharacterized	NC_037652.1	LG15	6585505	6595496	-0.7	4.8E-02
LOC409899	409899	putative sodium-dependent multivitamin transporter, transcript variant X4	NC_037643.1	LG6	6522504	6532893	-0.7	1.7E-02
LOC409151	409151	ataxin-2-like protein, transcript variant X2	NC_037639.1	LG2	15190161	15195383	-0.7	3.4E-02
LOC550724	550724	3-hydroxy-3-methylglutaryl-coenzyme A reductase, transcript variant X3	NC_037638.1	LG1	22804630	22816929	-0.7	3.3E-02
LOC411083	411083	sodium/potassium-transporting ATPase subunit alpha, transcript	NC_037642.1	LG5	6883507	7002055	-0.7	3.8E-02

		variant X6						
LOC551904	551904	glycerol-3-phosphate dehydrogenase, mitochondrial, transcript variant X2	NC_037645.1	LG8	7772817	7781131	-0.8	1.8E-02
LOC412612	412612	carbohydrate sulfotransferase 4, transcript variant X1	NC_037645.1	LG8	9450384	9454325	-0.8	8.0E-03
LOC413987	413987	polypeptide N-acetylgalactosaminyltransferase 5, transcript variant X3	NC_037640.1	LG3	3770421	3797949	-0.8	4.4E-02
LOC408820	408820	DDB1- and CUL4-associated factor 8, transcript variant X2	NC_037641.1	LG4	3728137	3732474	-0.8	2.3E-02
LOC410819	410819	proton channel OtopLc, transcript variant X5	NC_037639.1	LG2	14094621	14124159	-0.8	2.0E-02
LOC409180	409180	4-hydroxybutyrate coenzyme A transferase-like, transcript variant X2	NC_037652.1	LG15	3781273	3785782	-0.8	3.1E-02
LOC552074	552074	probable phospholipid-transporting ATPase IA, transcript variant X6	NC_037639.1	LG2	12599450	12610802	-0.8	3.9E-02
LOC726102	726102	transmembrane protein 135	NC_037642.1	LG5	12219572	12223358	-0.8	3.8E-02
LOC726497	726497	autophagy-related protein 2 homolog B, transcript variant X1	NC_037642.1	LG5	9703446	9712115	-0.8	3.3E-02
KAT2A	552646	K(lysine) acetyltransferase 2A	NC_037653.1	LG16	1285857	1289377	-0.8	4.4E-02
LOC551846	551846	membralin, transcript variant X3	NC_037651.1	LG14	5867682	5946552	-0.8	4.9E-03
LOC552157	552157	SET domain-containing protein SmydA-8, transcript variant X3	NC_037644.1	LG7	8729848	8735780	-0.8	4.7E-02
LOC409715	409715	bumetanide-sensitive sodium-(potassium)-chloride cotransporter, transcript variant X2	NC_037650.1	LG13	10785896	10811170	-0.8	3.3E-02
LOC409271	409271	mediator of RNA polymerase II transcription subunit 12, transcript variant X1	NC_037643.1	LG6	16784738	16794537	-0.8	1.7E-02
Camkii	551691	calcium/calmodulin-dependent protein kinase II	NC_037641.1	LG4	5243020	5370926	-0.8	2.2E-02
LOC725192	725192	major facilitator superfamily domain-containing protein 6-A, transcript variant X1	NC_037647.1	LG10	4526954	4549185	-0.8	3.7E-02
LOC413366	413366	homeobox protein SIX1, transcript variant X1	NC_037642.1	LG5	7226738	7263231	-0.8	4.2E-02
LOC408501	408501	serine/threonine-protein kinase/endoribonuclease IRE1, transcript variant X2	NC_037651.1	LG14	10640611	10645204	-0.8	1.9E-02

LOC551881	551881	ATP-dependent helicase brm, transcript variant X2	NC_037650.1	LG13	791288	805196	-0.8	4.1E-02
LOC408520	408520	upstream activation factor subunit spp27	NC_037652.1	LG15	7189452	7193590	-0.8	2.9E-02
LOC724603	724603	receptor-type guanylate cyclase Gyc76C-like	NC_037640.1	LG3	6253964	6319730	-0.8	5.0E-02
LOC413091	413091	serrate RNA effector molecule homolog, transcript variant X1	NC_037648.1	LG11	15953730	15960254	-0.8	2.6E-02
LOC102656448	102656448	glycerol-3-phosphate dehydrogenase	NC_037646.1	LG9	2862124	2874972	-0.8	4.1E-02
LOC100577272	100577272	uncharacterized	NC_037641.1	LG4	13157176	13163053	-0.8	5.8E-03
LOC100577198	100577198	uncharacterized	NC_037647.1	LG10	419083	479550	-0.8	3.9E-02
LOC409970	409970	thrombospondin type-1 domain-containing protein 4, transcript variant X6	NC_037638.1	LG1	16691313	16717838	-0.8	2.0E-02
LOC100576383	100576383	mannosyl-oligosaccharide 1,2-alpha-mannosidase IA, transcript variant X6	NC_037641.1	LG4	5975873	6453452	-0.8	4.7E-02
LOC100578253	100578253	Golgi integral membrane protein 4, transcript variant X6	NC_037645.1	LG8	8975197	8986447	-0.8	2.2E-02
LOC410545	410545	CLIP-associating protein 1-A, transcript variant X11	NC_037651.1	LG14	9304449	9332429	-0.8	4.8E-02
LOC409624	409624	acetyl-coenzyme A synthetase	NC_037644.1	LG7	5009044	5031198	-0.8	1.5E-02
Mblk-1	408521	transcription factor mblk-1-like	NC_037652.1	LG15	6989419	7038341	-0.8	1.6E-02
LOC408915	408915	protein couch potato, transcript variant X6	NC_037644.1	LG7	12538654	12721782	-0.8	4.1E-02
LOC551967	551967	exostosin-1, transcript variant X1	NC_037647.1	LG10	9422791	9427647	-0.8	2.7E-03
LOC551396	551396	integrator complex subunit 4	NC_037640.1	LG3	3305679	3309316	-0.8	3.6E-02
LOC408456	408456	facilitated trehalose transporter Tret1-2 homolog, transcript variant X2	NC_037651.1	LG14	7199600	7207215	-0.8	8.8E-03
LOC409260	409260	short-chain dehydrogenase/reductase family 16C member 6, transcript variant X1	NC_037648.1	LG11	13659681	13671130	-0.8	4.4E-02
LOC411113	411113	solute carrier family 12 member 4, transcript variant X4	NC_037642.1	LG5	10204340	10344216	-0.8	4.5E-02
LOC724235	724235	uncharacterized	NC_037638.1	LG1	12173154	12191984	-0.8	1.1E-02
LOC411849	411849	probable sulfite oxidase, mitochondrial	NC_037652.1	LG15	3698585	3701262	-0.8	2.4E-02

LOC412117	412117	mediator of RNA polymerase II transcription subunit 15, transcript variant X4	NC_037638.1	LG1	15104060	15111874	-0.9	4.6E-02
LOC100576882	100576882	uncharacterized	NC_037647.1	LG10	9894788	9925239	-0.9	2.3E-02
LOC100577517	100577517	uncharacterized	NC_037643.1	LG6	7439413	7632596	-0.9	4.7E-02
LOC725546	725546	DDB1- and CUL4-associated factor 6, transcript variant X2	NC_037641.1	LG4	5232795	5240940	-0.9	1.7E-03
LOC725220	725220	homeobox protein Nkx-6.1	NC_037645.1	LG8	1395517	1431135	-0.9	2.3E-02
LOC726134	726134	carboxylesterase	NC_037644.1	LG7	8750217	8757061	-0.9	3.7E-02
LOC408809	408809	diacylglycerol kinase theta, transcript variant X2	NC_037641.1	LG4	7989171	8016620	-0.9	2.1E-02
LOC551144	551144	uncharacterized	NC_037648.1	LG11	2444116	2477655	-0.9	4.3E-02
LOC726254	726254	uncharacterized	NC_037641.1	LG4	11213998	11227021	-0.9	3.2E-02
LOC409087	409087	D-beta-hydroxybutyrate dehydrogenase, mitochondrial, transcript variant X1	NC_037639.1	LG2	15252365	15255221	-0.9	9.6E-03
LOC413473	413473	GTPase-activating Rap/Ran-GAP domain-like protein 3, transcript variant X7	NC_037643.1	LG6	16657298	16680330	-0.9	9.0E-03
LOC552048	552048	flocculation protein FLO11, transcript variant X2	NC_037644.1	LG7	12742945	12753145	-0.9	3.9E-03
LOC551168	551168	protein sidekick, transcript variant X5	NC_037640.1	LG3	10173536	10219164	-0.9	3.9E-02
LOC410710	410710	zinc transporter ZIP13 homolog, transcript variant X2	NC_037638.1	LG1	15619728	15633525	-0.9	2.1E-03
LOC409893	409893	protein alan shepard, transcript variant X14	NC_037640.1	LG3	8215549	8249707	-0.9	1.5E-02
LOC413858	413858	uncharacterized	NC_037640.1	LG3	4566240	4581521	-0.9	6.8E-03
LOC726358	726358	huntingtin, transcript variant X1	NC_037644.1	LG7	2984163	2994696	-0.9	1.3E-03
LOC409897	409897	probable multidrug resistance-associated protein lethal(2)03659, transcript variant X1	NC_037643.1	LG6	6576315	6594391	-0.9	2.9E-02
LOC113218564	113218564	uncharacterized	NC_037643.1	LG6	4227985	4237141	-0.9	2.9E-02
LOC412876	412876	pyruvate carboxylase, mitochondrial, transcript variant X2	NC_037650.1	LG13	10899999	10928390	-0.9	3.2E-02
LOC410352	410352	chromatin-remodeling ATPase INO80, transcript variant X4	NC_037648.1	LG11	13423197	13439264	-0.9	1.0E-02
LOC412436	412436	protein CASC1, transcript variant	NC_037638.1	LG1	14178115	14216414	-0.9	4.1E-02

		X4						
LOC409111	409111	serine/threonine-protein kinase minibrain	NC_037639.1	LG2	1936550	2000823	-0.9	1.8E-02
LOC100577343	100577343	uncharacterized	NC_037647.1	LG10	8080265	8100141	-0.9	2.5E-02
LOC725782	725782	alpha-actinin, sarcomeric, transcript variant X3	NC_037648.1	LG11	15823367	15835167	-0.9	1.8E-02
Antp	406077	homeotic protein antennapedia	NC_037653.1	LG16	3881429	4037081	-0.9	3.3E-02
LOC410363	410363	uncharacterized	NC_037649.1	LG12	6744576	6769869	-0.9	4.2E-02
LOC552502	552502	protein KIAA0100, transcript variant X2	NC_037638.1	LG1	10856522	10865414	-0.9	3.4E-03
GluCl	408450	glutamate-gated chloride channel	NC_037651.1	LG14	5177026	5214539	-0.9	9.6E-03
LOC412959	412959	uncharacterized	NC_037645.1	LG8	12684341	12702391	-0.9	3.7E-02
LOC552371	552371	ATP-binding cassette sub-family G member 4, transcript variant X5	NC_037638.1	LG1	11592017	11603963	-0.9	2.5E-02
LOC551031	551031	uncharacterized	NC_037638.1	LG1	12312260	12348810	-0.9	3.8E-03
LOC409981	409981	titin	NC_037641.1	LG4	10230114	10239841	-0.9	4.2E-02
LOC725697	725697	protein dead ringer, transcript variant X3	NC_037639.1	LG2	14438609	14527124	-0.9	3.3E-02
LOC724561	724561	E3 ubiquitin-protein ligase DCST1, transcript variant X1	NC_037642.1	LG5	2781433	2787837	-0.9	3.3E-02
LOC552397	552397	Krueppel-like factor 6, transcript variant X2	NC_037649.1	LG12	9699271	9931975	-0.9	4.5E-03
LOC724250	724250	serine protease snake	NC_037652.1	LG15	3812648	3815066	-0.9	4.3E-02
LOC410825	410825	leucine-rich repeat and immunoglobulin-like domain-containing nogo receptor-interacting protein 3	NC_037639.1	LG2	13730577	13735477	-0.9	4.5E-02
LOC726598	726598	pre-mRNA-splicing factor ATP-dependent RNA helicase DHX16	NC_037650.1	LG13	4453533	4457515	-0.9	3.4E-03
LOC410042	410042	glyoxylate reductase/hydroxypyruvate reductase	NC_037649.1	LG12	8500013	8502775	-0.9	4.1E-02
LOC552099	552099	homeotic protein empty spiracles	NC_037652.1	LG15	3181720	3229507	-0.9	4.8E-02
LOC102655100	102655100	protein Cep78 homolog	NC_037639.1	LG2	9940530	9943372	-0.9	2.1E-03
LOC113218632	113218632	uncharacterized	NC_037638.1	LG1	12493604	12502742	-0.9	1.9E-02
LOC552259	552259	staphylococcal nuclease domain-containing protein 1	NC_037644.1	LG7	9554706	9559092	-1.0	4.6E-02
LOC412735	412735	decaprenyl-diphosphate synthase	NC_037649.1	LG12	2823741	2990185	-1.0	3.9E-02

		subunit 1						
LOC410171	410171	filamin-A, transcript variant X1	NC_037647.1	LG10	9958726	9978868	-1.0	1.0E-03
LOC726471	726471	SET domain-containing protein SmydA-8, transcript variant X2	NC_037649.1	LG12	8646651	8649782	-1.0	2.9E-02
LOC409267	409267	glycogen phosphorylase	NC_037646.1	LG9	7741309	7748359	-1.0	8.6E-03
LOC725936	725936	titin homolog	NC_037644.1	LG7	8767723	8847638	-1.0	1.1E-02
LOC551883	551883	voltage-dependent T-type calcium channel subunit alpha-1G	NC_037648.1	LG11	6188331	6386759	-1.0	1.3E-02
LOC724295	724295	protein phosphatase 1 regulatory subunit 3C-B	NC_037652.1	LG15	3816871	3834574	-1.0	4.6E-02
LOC409903	409903	GTP-binding protein RAD, transcript variant X2	NC_037647.1	LG10	5639729	5680006	-1.0	2.8E-02
LOC409650	409650	solute carrier organic anion transporter family member 2A1, transcript variant X1	NC_037647.1	LG10	10889541	10896973	-1.0	1.1E-03
LOC408465	408465	vesicle-associated membrane protein 2, transcript variant X1	NC_037651.1	LG14	8280795	8318641	-1.0	3.5E-04
LOC726736	726736	laminin subunit beta-1, transcript variant X1	NC_037650.1	LG13	2106765	2126275	-1.0	2.2E-02
LOC551448	551448	ribose-phosphate pyrophosphokinase 1, transcript variant X2	NC_037638.1	LG1	29940	39215	-1.0	8.1E-03
LOC413721	413721	uncharacterized	NC_037644.1	LG7	52745	106490	-1.0	2.8E-02
LOC411047	411047	intraflagellar transport protein 88 homolog, transcript variant X7	NC_037641.1	LG4	4788059	4797045	-1.0	3.2E-02
LOC727598	727598	probable cytochrome P450 6a13, transcript variant X2	NC_037650.1	LG13	10738114	10742837	-1.0	6.1E-03
LOC410353	410353	CUGBP Elav-like family member 4, transcript variant X11	NC_037649.1	LG12	46221	576852	-1.0	6.2E-03
LOC552674	552674	shugoshin 2, transcript variant X1	NC_037646.1	LG9	11094002	11098190	-1.0	2.7E-02
LOC102654257	102654257	uncharacterized	NC_037638.1	LG1	27581040	27585099	-1.0	1.1E-02
LOC410059	410059	probable citrate synthase 2, mitochondrial	NC_037649.1	LG12	10064863	10076340	-1.0	2.7E-03
LOC102654831	102654831	uncharacterized	NC_037646.1	LG9	12090806	12249170	-1.0	4.7E-02
LOC551670	551670	argininosuccinate lyase, transcript variant X1	NC_037638.1	LG1	11523225	11526267	-1.0	2.9E-02
LOC552421	552421	glycogenin-1, transcript variant X1	NC_037646.1	LG9	7849136	7863244	-1.0	1.1E-02
LOC725964	725964	uncharacterized	NC_037643.1	LG6	4908510	4973111	-1.0	1.6E-02
LOC10057855	100578557	solute carrier family 46 member	NC_037641.1	LG4	3467018	3480468	-1.0	3.8E-04

7		3, transcript variant X1						
LOC409517	409517	digestive cysteine proteinase 1	NC_037638.1	LG1	5036235	5039807	-1.0	1.3E-02
LOC410642	410642	homeobox protein abdominal-B, transcript variant X3	NC_037653.1	LG16	3302672	3316734	-1.0	1.0E-02
LOC411910	411910	homeobox protein 2-like	NC_037638.1	LG1	14210225	14213478	-1.0	4.1E-02
Mir6058	104794350	microRNA 6058	NC_037644.1	LG7	3758281	3758357	-1.0	3.9E-02
LOC727456	727456	glucose-6-phosphate 1-epimerase	NC_037639.1	LG2	161816	190029	-1.0	3.4E-03
LOC724706	724706	trichohyalin, transcript variant X6	NC_037644.1	LG7	13738786	13750818	-1.0	1.7E-02
LOC724861	724861	transcription factor Maf, transcript variant X1	NC_037643.1	LG6	16410236	16418506	-1.0	9.0E-03
LOC410506	410506	uncharacterized	NC_037651.1	LG14	7610807	7660371	-1.0	2.0E-03
LOC727269	727269	zinc finger protein 271, transcript variant X1	NC_037647.1	LG10	409400	412566	-1.0	5.0E-02
LOC412777	412777	sodium/potassium/calcium exchanger Nckx30C, transcript variant X1	NC_037638.1	LG1	20279300	20333724	-1.0	1.6E-02
LOC107964896	107964896	uncharacterized	NC_037645.1	LG8	12378161	12378908	-1.0	3.2E-02
LOC412273	412273	probable chitinase 10, transcript variant X3	NC_037648.1	LG11	15458480	15471726	-1.0	4.1E-02
LOC409712	409712	short/branched chain specific acyl-CoA dehydrogenase, mitochondrial	NC_037652.1	LG15	7870499	7874116	-1.0	2.4E-02
LOC411983	411983	putative fatty acyl-CoA reductase	NC_037638.1	LG1	14072364	14078430	-1.0	2.8E-02
LOC724172	724172	neurobeachin, transcript variant X8	NC_037643.1	LG6	9764269	10200454	-1.1	8.4E-03
LOC412670	412670	probable phosphoserine aminotransferase	NC_037641.1	LG4	146531	149435	-1.1	7.1E-04
LOC724864	724864	TWiK family of potassium channels protein 18, transcript variant X2	NC_037645.1	LG8	6206046	6222091	-1.1	4.8E-02
Oa1	406068	octopamine receptor	NC_037652.1	LG15	3310369	3326664	-1.1	3.8E-02
LOC724865	724865	ABC transporter G family member 20, transcript variant X1	NC_037652.1	LG15	818668	904370	-1.1	1.6E-02
LOC409643	409643	failed axon connections, transcript variant X2	NC_037640.1	LG3	8053539	8085030	-1.1	2.0E-03
LOC408444	408444	uncharacterized	NC_037651.1	LG14	4526896	4599343	-1.1	3.2E-03
LOC727146	727146	sphingomyelin phosphodiesterase, transcript variant X3	NC_037650.1	LG13	2298085	2340846	-1.1	3.1E-03
LOC551920	551920	mucin-5AC	NC_037642.1	LG5	12058790	12076188	-1.1	1.0E-02

LOC100578445	100578445	uncharacterized	NC_037638.1	LG1	5071914	5077640	-1.1	2.1E-02
LOC409250	409250	beta-ureidopropionase	NC_037645.1	LG8	2224065	2226802	-1.1	2.9E-02
LOC411307	411307	mitochondrial enolase superfamily member 1	NC_037646.1	LG9	10893442	10895515	-1.1	9.3E-03
LOC413782	413782	cartilage oligomeric matrix protein, transcript variant X2	NC_037643.1	LG6	16848239	16857688	-1.1	3.7E-02
LOC102655838	102655838	protein transport protein sec31	NC_037641.1	LG4	5455927	5464954	-1.1	1.7E-02
LOC412788	412788	scavenger receptor class B member 1, transcript variant X4	NC_037649.1	LG12	9277013	9302506	-1.1	5.3E-03
LOC409771	409771	potassium channel subfamily K member 18, transcript variant X2	NC_037645.1	LG8	6225905	6252412	-1.1	1.3E-03
LOC100578121	100578121	uncharacterized	NC_037649.1	LG12	11477045	11484980	-1.1	3.5E-03
LOC409919	409919	excitatory amino acid transporter 1, transcript variant X4	NC_037648.1	LG11	14363099	14374104	-1.1	8.7E-03
LOC726990	726990	uncharacterized	NC_037642.1	LG5	13833556	13839153	-1.1	1.1E-02
LOC551053	551053	facilitated trehalose transporter Tret1, transcript variant X4	NC_037650.1	LG13	2388556	2406317	-1.1	1.3E-02
LOC408293	408293	exostosin-1, transcript variant X1	NC_037647.1	LG10	9285761	9293522	-1.1	3.4E-03
LOC102656439	102656439	adenosine deaminase 2	NC_037650.1	LG13	7304111	7308050	-1.1	4.8E-02
LOC726981	726981	circadian clock-controlled protein	NC_037646.1	LG9	6887843	6891778	-1.1	4.7E-02
LOC102655356	102655356	thyrotropin-releasing hormone receptor	NC_037643.1	LG6	15511589	15522815	-1.1	1.8E-02
LOC107964994	107964994	uncharacterized	NC_037646.1	LG9	9739567	9741117	-1.1	2.2E-03
LOC410380	410380	neprilysin-2, transcript variant X2	NC_037649.1	LG12	11248840	11265289	-1.1	1.5E-02
LOC724831	724831	uncharacterized	NC_037642.1	LG5	267261	320977	-1.1	1.7E-03
LOC107965199	107965199	UDP-glucuronosyltransferase 1-1, transcript variant X4	NC_037648.1	LG11	10467257	10471396	-1.1	1.2E-02
LOC552286	552286	acetyl-CoA carboxylase, transcript variant X1	NC_037649.1	LG12	7849643	7872822	-1.1	1.5E-02
LOC408818	408818	hexokinase-1	NC_037641.1	LG4	4782182	4791706	-1.1	2.1E-02
LOC410947	410947	tyrosine decarboxylase	NC_037640.1	LG3	6062230	6065649	-1.1	8.6E-03
LOC408295	408295	probable serine/threonine-protein kinase DDB_G0282963, transcript variant X3	NC_037647.1	LG10	9252009	9277415	-1.1	3.2E-02
LOC100577113	100577113	uncharacterized	NC_037638.1	LG1	8183447	8185628	-1.1	2.3E-02

LOC102655153	102655153	facilitated trehalose transporter Tret1-like	NC_037641.1	LG4	6267551	6269261	-1.1	1.9E-02
LOC100576703	100576703	sarcalumenin, transcript variant X6	NC_037643.1	LG6	9016424	9025292	-1.1	1.6E-02
LOC100578919	100578919	tax1-binding protein 1 homolog, transcript variant X1	NC_037641.1	LG4	2635122	2640095	-1.1	1.5E-02
5-HT2alpha	411323	serotonin receptor	NC_037646.1	LG9	8766576	8797600	-1.1	6.3E-03
LOC102656294	102656294	uncharacterized	NC_037645.1	LG8	8389297	8398519	-1.1	4.3E-02
LOC552836	552836	uncharacterized	NC_037652.1	LG15	6976717	6981974	-1.1	2.5E-02
LOC409208	409208	bumetanide-sensitive sodium-(potassium)-chloride cotransporter	NC_037649.1	LG12	7331981	7345379	-1.1	3.2E-03
LOC409759	409759	serine-rich adhesin for platelets	NC_037648.1	LG11	15837214	15856267	-1.2	3.0E-03
LOC408564	408564	long-chain fatty acid transport protein 4, transcript variant X1	NC_037653.1	LG16	6635505	6644798	-1.2	7.1E-04
LOC724524	724524	scm-like with four MBT domains protein 2, transcript variant X1	NC_037642.1	LG5	9924193	9929029	-1.2	2.6E-03
LOC411457	411457	galactosylgalactosylxylosylprotein 3-beta-glucuronosyltransferase P, transcript variant X6	NC_037648.1	LG11	14406508	14414597	-1.2	7.1E-06
LOC413739	413739	synaptic vesicle glycoprotein 2C, transcript variant X2	NC_037645.1	LG8	1688557	1707686	-1.2	5.8E-03
LOC551623	551623	nucleolysin TIAR, transcript variant X2	NC_037638.1	LG1	26349896	26815254	-1.2	1.3E-02
Grd	413803	GABA-gated ion channel	NC_037638.1	LG1	6428662	6469994	-1.2	2.7E-03
Or109	100577813	odorant receptor 109	NC_037649.1	LG12	4927844	4930736	-1.2	2.6E-02
LOC100578388	100578388	uncharacterized	NC_037648.1	LG11	16002590	16021363	-1.2	1.6E-02
LOC412230	412230	dynein heavy chain 3, axonemal, transcript variant X1	NC_037652.1	LG15	9061000	9079509	-1.2	8.4E-03
LOC410671	410671	proto-oncogene tyrosine-protein kinase ROS, transcript variant X1	NC_037638.1	LG1	21701665	21727456	-1.2	1.3E-02
LOC411338	411338	WD repeat-containing protein 35	NC_037646.1	LG9	7687128	7690995	-1.2	3.6E-02
LOC100578059	100578059	vasorin, transcript variant X1	NC_037643.1	LG6	16527988	16543772	-1.2	1.9E-03
LOC100576249	100576249	uncharacterized	NC_037639.1	LG2	5547277	5555198	-1.2	1.3E-03
LOC100578920	100578920	zinc finger protein 251	NC_037651.1	LG14	2602509	2611448	-1.2	5.0E-02
LOC724252	724252	homeotic protein deformed	NC_037653.1	LG16	4280665	4291655	-1.2	1.6E-02
LOC10796610	107966103	uncharacterized	NC_037639.1	LG2	9945125	9949640	-1.2	3.2E-03

3								
LOC413478	413478	spermine oxidase	NC_037652.1	LG15	6530699	6533243	-1.2	2.8E-03
LOC724281	724281	probable serine/threonine-protein kinase mps1, transcript variant X2	NC_037638.1	LG1	15261405	15335892	-1.2	5.3E-03
LOC100577231	100577231	uncharacterized	NC_037642.1	LG5	5024522	5118524	-1.2	1.7E-02
LOC724946	724946	cytochrome P450 6a14-like	NC_037650.1	LG13	10541731	10546300	-1.2	8.9E-03
LOC551197	551197	probable cytochrome P450 6a13	NC_037651.1	LG14	374523	378498	-1.3	1.2E-02
LOC411685	411685	ATP-binding cassette sub-family D member 1	NC_037644.1	LG7	150367	153937	-1.3	1.5E-05
LOC413408	413408	scavenger receptor class B member 1	NC_037653.1	LG16	6263230	6289809	-1.3	3.2E-02
LOC107965421	107965421	uncharacterized	NC_037650.1	LG13	3439363	3448350	-1.3	3.4E-02
LOC551094	551094	fatty-acid amide hydrolase 2-B	NC_037649.1	LG12	3219402	3239836	-1.3	1.7E-02
LOC100577028	100577028	insulin-like growth factor I, transcript variant X2	NC_037645.1	LG8	4175629	4185151	-1.3	7.1E-04
LOC113218910	113218910	uncharacterized	NC_037638.1	LG1	12461591	12468303	-1.3	1.8E-02
Mrjp6	406149	major royal jelly protein 6	NC_037648.1	LG11	2286675	2289715	-1.3	1.3E-03
LOC551437	551437	alpha-N-acetylglucosaminidase, transcript variant X2	NC_037644.1	LG7	2082573	2089958	-1.3	1.0E-02
LOC102654730	102654730	uncharacterized	NC_037650.1	LG13	2611215	2614356	-1.3	1.6E-02
LOC409173	409173	esterase FE4	NC_037641.1	LG4	5944397	5947076	-1.3	1.2E-02
LOC410021	410021	protein Skeletor, isoforms B/C, transcript variant X2	NC_037638.1	LG1	15659500	15720213	-1.3	2.7E-03
LOC727593	727593	UDP-glucuronosyltransferase 2C1	NC_037648.1	LG11	10461904	10466548	-1.3	9.1E-04
LOC113219370	113219370	organic cation transporter protein-like	NW_020555886.1	uncharacterized	8785	19479	-1.3	3.4E-03
LOC412467	412467	bifunctional purine biosynthesis protein PURH	NC_037646.1	LG9	5768642	5771517	-1.3	4.0E-03
LOC113218561	113218561	cytoplasmic dynein 2 heavy chain 1-like	NC_037643.1	LG6	2238792	2247558	-1.3	4.4E-02
LOC102654393	102654393	major royal jelly protein 2-like	NC_037648.1	LG11	2297915	2298564	-1.3	9.3E-03
LOC412222	412222	uncharacterized	NC_037648.1	LG11	6826251	6862437	-1.4	5.0E-02
LOC102656717	102656717	uncharacterized	NC_037648.1	LG11	1808816	1816503	-1.4	3.8E-02
LOC411140	411140	putative aldehyde dehydrogenase family 7 member A1 homolog	NC_037643.1	LG6	17075160	17078082	-1.4	2.0E-03

LOC107965219	107965219	feline leukemia virus subgroup C receptor-related protein 2, transcript variant X1	NC_037648.1	LG11	8353573	8359916	-1.4	9.1E-03
LOC725123	725123	activity-regulated cytoskeleton associated protein 2	NC_037640.1	LG3	12863157	12867241	-1.4	3.7E-02
LOC413471	413471	sodium/potassium-transporting ATPase subunit alpha-B	NC_037638.1	LG1	9268131	9271607	-1.4	1.8E-02
LOC411036	411036	two pore potassium channel protein sup-9, transcript variant X1	NC_037641.1	LG4	6572857	6689476	-1.4	3.0E-03
LOC412570	412570	RYamide receptor, transcript variant X2	NC_037649.1	LG12	9166071	9184225	-1.4	3.6E-04
LOC725400	725400	4-hydroxyphenylpyruvate dioxygenase, transcript variant X2	NC_037640.1	LG3	10246959	10253406	-1.4	1.1E-02
LOC551850	551850	uncharacterized	NC_037640.1	LG3	2607888	2645475	-1.4	2.4E-03
LOC102656473	102656473	CB1 cannabinoid receptor-interacting protein 1	NC_037649.1	LG12	7811417	7822865	-1.4	3.7E-02
LOC113219054	113219054	probable phosphoserine aminotransferase	NC_037647.1	LG10	5897572	5905695	-1.5	5.9E-03
LOC102654151	102654151	uncharacterized	NC_037653.1	LG16	1892438	1894952	-1.5	4.7E-02
eve	724845	segmentation protein even-skipped	NC_037651.1	LG14	1545762	1551144	-1.5	3.2E-02
LOC724552	724552	elongation of very long chain fatty acids protein AAEL008004	NC_037653.1	LG16	2466367	2479648	-1.5	1.1E-02
LOC550749	550749	C-1-tetrahydrofolate synthase, cytoplasmic, transcript variant X2	NC_037642.1	LG5	3001133	3007290	-1.5	3.0E-03
LOC100577717	100577717	uncharacterized	NC_037642.1	LG5	9883630	9888491	-1.5	7.1E-06
Or2	725384	odorant receptor 2	NC_037638.1	LG1	5723757	5749095	-1.5	6.3E-03
LOC113219372	113219372	organic cation transporter protein-like	NW_020555891.1	uncharacterized	7841	13899	-1.5	2.9E-03
LOC100576638	100576638	uncharacterized	NC_037640.1	LG3	6923648	6926794	-1.5	2.2E-02
LOC725590	725590	homeobox protein B-H2-like	NC_037640.1	LG3	3874732	3908709	-1.5	9.9E-04
LOC102654777	102654777	organic cation transporter protein, transcript variant X6	NC_037644.1	LG7	10701571	10711503	-1.5	3.4E-04
LOC410657	410657	inhibitory POU protein, transcript variant X2	NC_037653.1	LG16	6321560	6360034	-1.6	1.7E-02
LOC107964635	107964635	uncharacterized	NC_037643.1	LG6	3664846	3667362	-1.6	6.9E-03
LOC113218752	113218752	uncharacterized	NC_037641.1	LG4	5972614	6001185	-1.6	1.2E-02

LOC724515	724515	centromere-associated protein E	NC_037642.1	LG5	4233791	4242240	-1.6	8.9E-03
Mir3754	100629117	microRNA 3754	NC_037646.1	LG9	12029003	12029131	-1.7	3.4E-02
LOC412797	412797	facilitated trehalose transporter Tret1	NC_037649.1	LG12	1373461	1376596	-1.7	1.7E-03
LOC413568	413568	probable G-protein coupled receptor 158	NC_037645.1	LG8	9161606	9330054	-1.7	6.8E-06
CPR19	413115	cuticular protein 19	NC_037641.1	LG4	8665666	8667293	-1.8	1.3E-02
LOC102656724	102656724	uncharacterized	NC_037647.1	LG10	10672403	10674934	-1.8	6.8E-03
Est-6	410928	venom carboxylesterase-6	NC_037640.1	LG3	9461728	9464050	-1.8	3.4E-03
LOC100576591	100576591	uncharacterized	NC_037651.1	LG14	3404388	3410945	-1.8	7.7E-03
LOC550958	550958	uncharacterized	NC_037651.1	LG14	3410782	3412416	-1.8	1.4E-02
LOC726362	726362	4-coumarate--CoA ligase 1, transcript variant X1	NC_037644.1	LG7	4974286	4991578	-1.8	1.2E-03
LOC100578213	100578213	probable serine/threonine-protein kinase dyrk2	NC_037639.1	LG2	11770170	11772831	-1.9	9.4E-07
Obp2	406103	odorant binding protein 2	NC_037646.1	LG9	11974257	11975390	-1.9	7.4E-06
LOC113218711	113218711	uncharacterized	NC_037641.1	LG4	4451496	4456060	-1.9	1.6E-03
Obp6	406109	odorant binding protein 6	NC_037646.1	LG9	11946731	11971739	-2.0	1.3E-03
LOC552024	552024	myo-inositol 2-dehydrogenase	NC_037645.1	LG8	8626452	8629999	-2.1	4.3E-06
LOC726446	726446	uncharacterized	NC_037638.1	LG1	367913	369386	-2.8	3.2E-07
LOC102654654	102654654	uncharacterized	NC_037645.1	LG8	9194027	9201573	-2.9	6.1E-06

[a]Gene symbol, gene ID, genomic accession, chromosome, start, and end based on the BeeBase gene identifiers of the Honey bee genome (Amel_HAv3.1; ftp://ftp.ncbi.nlm.nih.gov/genomes/Apis_mellifera: National Center for Biotechnology Information (Bethesda (MD) [24]: National Library of Medicine (US), National Center for Biotechnology Information; [1988] - [cited 2024 Feb 15]). Available from https://www.ncbi.nlm.nih.gov/
[b]Gene annotation based on g:profiler search for biological process terms, considering a depth of two hierarchical levels [30]. ShinyGO v.077 shearch (https://biosnips.org/category/next-generation-sequencing-analysis/ [32] - [cited 2024 Feb 15]).
[c]Log2 fold change was calculated using the DESeq2 R package (Anders & Huber, 2010) and edge R Bioconductor package (Robinson et al., 2010) based on the raw read counts.

Table S8. Characteristics of down-regulated unique DEGs from LVG-V versus LVG-C comparison (p<0.05, Log2FC).

Gene symbol	Gene ID	Gene annotation	Genomic accession	Chromosome	Start	End	log2FC	p-value
LOC725461	725461	leucine-rich repeat protein 1	NC_037647.1	LG10	12030450	12032059	-2.0	7.2E-03
LOC113218859	1.13E+08	uncharacterized	NC_037643.1	LG6	14679009	14683880	-1.7	4.2E-02
LOC552313	552313	sterol O-acyltransferase 1	NC_037648.1	LG11	8114746	8122087	-1.5	2.9E-03
LOC724187	724187	toll-like receptor 4, transcript variant X4	NC_037639.1	LG2	11056209	11060453	-1.5	1.3E-02
LOC100576623	1.01E+08	piggyBac transposable element-derived protein 4	NC_037639.1	LG2	1051119	1052985	-1.4	4.7E-02
LOC411226	411226	tubulin epsilon chain, transcript variant X3	NC_037644.1	LG7	3615704	3618215	-1.4	4.7E-02
LOC724513	724513	zinc finger protein 816	NC_037642.1	LG5	1933260	1936480	-1.3	2.9E-02
Crzr	409042	corazonin receptor	NC_037638.1	LG1	10198903	10212094	-1.3	1.5E-02
LOC102654930	1.03E+08	uncharacterized	NC_037643.1	LG6	12175293	12181058	-1.3	2.2E-02
LOC724934	724934	paired mesoderm homeobox protein 2A	NC_037645.1	LG8	9527002	9548774	-1.2	4.3E-02
LOC412569	412569	probable 2-oxoglutarate dehydrogenase E1 component DHKTD1 homolog, mitochondrial, transcript variant X2	NC_037647.1	LG10	8110582	8116557	-1.2	1.8E-02
LOC726650	726650	uncharacterized	NC_037652.1	LG15	6523658	6530839	-1.2	1.7E-03
LOC551918	551918	chromatin assembly factor 1 subunit B	NC_037641.1	LG4	3679392	3681635	-1.2	4.7E-02
LOC102653826	1.03E+08	uncharacterized	NC_037640.1	LG3	12650650	12654378	-1.1	4.9E-02
LOC725178	725178	chitooligosaccharidolytic beta-N-acetylglucosaminidase	NC_037646.1	LG9	7546823	7550400	-1.1	4.5E-02
LOC550918	550918	ATP-binding cassette sub-family G member 4, transcript variant X1	NC_037641.1	LG4	12583497	12592860	-1.1	1.9E-02
LOC412198	412198	UDP-glucose:glycoprotein glucosyltransferase, transcript variant X2	NC_037647.1	LG10	10105234	10113110	-1.1	3.4E-02
LOC724703	724703	modular serine protease	NC_037643.1	LG6	5361607	5365983	-1.0	1.7E-02
LOC413740	413740	iodotyrosine deiodinase 1, transcript variant X2	NC_037645.1	LG8	3883175	3885050	-1.0	1.0E-02
LOC100577501	1.01E+08	nuclear pore membrane glycoprotein 210	NC_037638.1	LG1	22794118	22801610	-1.0	2.9E-02

LOC411602	411602	dynein heavy chain 6, axonemal, transcript variant X7	NC_037648.1	LG11	2649234	2672383	-1.0	2.8E-02
LOC100579040	1.01E+08	chondroitin sulfate synthase 2	NC_037648.1	LG11	14472319	14475344	-1.0	4.2E-02
LOC100577712	1.01E+08	uncharacterized	NC_037643.1	LG6	6622974	6628963	-1.0	3.5E-02
LOC413713	413713	glycosyltransferase 25 family member	NC_037638.1	LG1	10725659	10729172	-1.0	4.3E-02
LOC725993	725993	multidrug resistance-associated protein 7, transcript variant X1	NC_037641.1	LG4	11063345	11070389	-0.9	3.4E-02
LOC551712	551712	lipoyltransferase 1, mitochondrial, transcript variant X1	NC_037640.1	LG3	9929759	9932771	-0.9	4.5E-02
LOC410325	410325	trifunctional enzyme subunit alpha, mitochondrial	NC_037648.1	LG11	12223061	12227752	-0.9	4.2E-02
LOC413627	413627	uncharacterized	NC_037645.1	LG8	8235042	8238871	-0.9	1.6E-02
LOC724129	724129	pescadillo homolog, transcript variant X2	NC_037640.1	LG3	6130957	6134005	-0.9	2.2E-02
LOC411468	411468	GRAM domain-containing protein 1B, transcript variant X1	NC_037641.1	LG4	5691740	5698270	-0.8	4.7E-02
LOC409329	409329	mannose-1-phosphate guanyltransferase beta	NC_037648.1	LG11	14951260	14953755	-0.8	2.0E-02
LOC726944	726944	ATP-binding cassette sub-family G member 4, transcript variant X2	NC_037638.1	LG1	11565443	11582132	-0.8	3.4E-02
LOC724208	724208	serine protease snake	NC_037652.1	LG15	3808079	3810354	-0.8	3.6E-02
LOC552279	552279	sodium-independent sulfate anion transporter, transcript variant X2	NC_037645.1	LG8	2692962	2696422	-0.8	2.0E-02

[a]Gene symbol, gene ID, genomic accession, chromosome, start, and end based on the BeeBase gene identifiers of the Honey bee genome (Amel_HAv3.1; ftp://ftp.ncbi.nlm.nih.gov/genomes/Apis_mellifera: National Center for Biotechnology Information (Bethesda (MD) [24]: National Library of Medicine (US), National Center for Biotechnology Information; [1988] - [cited 2024 Feb 15]). Available from https://www.ncbi.nlm.nih.gov/

[b]Gene annotation based on g:profiler search for biological process terms, considering a depth of two hierarchical levels [30]. ShinyGO v.077 shearch (https://biosnips.org/category/next-generation-sequencing-analysis/ [32] - [cited 2024 Feb 15]).

[c]Log2 fold change was calculated using the DESeq2 R package (Anders & Huber, 2010) and edge R Bioconductor package (Robinson et al., 2010) based on the raw read counts.

Table S9. Characteristics of down-regulated DEGs shared between LVG-V versus LVG-C and HVG-V versus HVG-C comparisons ($p<0.05$, log2FC).

Gene symbol[a]	Gene ID[a]	Gene annotation[b]	Genomic accession[a]	Chromosome[a]	Start[a]	End[a]	log2FC[c]	p-value[c]
LOC724749	724749	uncharacterized	NC_037644.1	LG7	10984036	10990320	-2.0	3.7E-05
Mrjp7	409555	major royal jelly protein 7	NC_037648.1	LG11	2307856	2311141	-1.8	1.1E-03
LOC411893	411893	cytochrome P450 315a1, mitochondrial, transcript variant X2	NC_037640.1	LG3	8138875	8141925	-1.8	3.3E-03
LOC725215	725215	venom acid phosphatase Acph-1, transcript variant X1	NC_037642.1	LG5	13205306	13213175	-1.8	2.0E-04
Mrjp5	406116	major royal jelly protein 5	NC_037648.1	LG11	2291758	2298839	-1.5	2.2E-05
Mrjp4	406133	major royal jelly protein 4	NC_037648.1	LG11	2280526	2284092	-1.5	9.1E-03
LOC412630	412630	scavenger receptor class B member 1, transcript variant X5	NC_037649.1	LG12	7774616	7789073	-1.5	3.0E-02
LOC100577675	100577675	trifunctional purine biosynthetic protein adenosine-3, transcript variant X1	NC_037640.1	LG3	13077722	13083342	-1.5	1.1E-03
Mrjp2	406091	major royal jelly protein 2	NC_037648.1	LG11	2301533	2304856	-1.5	1.1E-02
LOC725967	725967	D-3-phosphoglycerate dehydrogenase, transcript variant X1	NC_037645.1	LG8	2704495	2708002	-1.5	5.4E-03
LOC552657	552657	phosphoribosylformylglycinamidine synthase	NC_037642.1	LG5	13344472	13351131	-1.4	1.1E-03
LOC551746	551746	uncharacterized	NC_037641.1	LG4	2443236	2619174	-1.4	9.3E-04
LOC727522	727522	uncharacterized	NC_037638.1	LG1	13311175	13319041	-1.3	3.4E-02
LOC413590	413590	alkylglycerol monooxygenase	NC_037641.1	LG4	10225195	10229269	-1.3	1.2E-03
LOC724933	724933	xanthine dehydrogenase	NC_037645.1	LG8	6409225	6427122	-1.3	1.0E-02
LOC100577053	100577053	glycine dehydrogenase (decarboxylating), mitochondrial, transcript variant X3	NC_037645.1	LG8	8206933	8211080	-1.3	5.8E-03
LOC413697	413697	PTS-dependent dihydroxyacetone kinase 1, dihydroxyacetone-binding subunit DhaK	NC_037652.1	LG15	9432459	9438572	-1.3	6.2E-03
LOC411597	411597	hemocytin, transcript variant X2	NC_037645.1	LG8	7303817	7318820	-1.3	4.2E-02
LOC726646	726646	probable cytochrome P450 6a13	NC_037638.1	LG1	7918849	7923739	-1.2	1.2E-03
LOC100578587	100578587	putative uncharacterized protein DDB_G0282133	NC_037639.1	LG2	2391734	2405838	-1.2	1.6E-03
LOC552249	552249	lysosomal alpha-mannosidase, transcript variant X1	NC_037641.1	LG4	10203630	10210650	-1.1	1.6E-02

LOC726935	726935	cardioacceleratory peptide receptor, transcript variant X2	NC_037653.1	LG16	1804042	1885820	-1.1	2.6E-02
LOC113218980	113218980	uncharacterized	NC_037646.1	LG9	5474150	5500614	-1.0	3.2E-02
LOC412619	412619	amidophosphoribosyltransferase, transcript variant X1	NC_037640.1	LG3	602649	607412	-1.0	1.7E-02
LOC726755	726755	pyrokinin-like receptor 2	NC_037641.1	LG4	1922920	1971322	-0.9	4.5E-02
LOC409517	409517	digestive cysteine proteinase 1	NC_037638.1	LG1	5036235	5039807	-0.9	4.5E-02
LOC413025	413025	peroxidasin, transcript variant X1	NC_037638.1	LG1	11075510	11089568	-0.9	4.5E-02
LOC411796	411796	serine hydroxymethyltransferase, transcript variant X2	NC_037648.1	LG11	7226906	7232254	-0.8	4.5E-02
LOC552505	552505	monocarboxylate transporter 9, transcript variant X1	NC_037644.1	LG7	12446292	12470578	-0.8	7.9E-03
LOC411657	411657	arylsulfatase B, transcript variant X3	NC_037642.1	LG5	10728956	10734880	-0.8	2.9E-02
LOC411355	411355	von Willebrand factor A domain-containing protein 8	NC_037643.1	LG6	5445314	5458762	-0.7	6.2E-03
LOC725552	725552	DNA-directed RNA polymerase III subunit RPC1, transcript variant X2	NC_037642.1	LG5	13121190	13140929	-0.6	3.4E-02
LOC410439	410439	basement membrane-specific heparan sulfate proteoglycan core protein, transcript variant X3	NC_037650.1	LG13	5024902	5169646	-0.6	3.4E-02

[a]Gene symbol, gene ID, genomic accession, chromosome, start, and end based on the BeeBase gene identifiers of the Honey bee genome (Amel_HAv3.1; ftp://ftp.ncbi.nlm.nih.gov/genomes/Apis_mellifera: National Center for Biotechnology Information (Bethesda (MD) [24]: National Library of Medicine (US), National Center for Biotechnology Information; [1988] - [cited 2024 Feb 15]). Available from https://www.ncbi.nlm.nih.gov/
[b]Gene annotation based on g:profiler search for biological process terms, considering a depth of two hierarchical levels [30]. ShinyGO v.077 shearch (https://biosnips.org/category/next-generation-sequencing-analysis/ [32] - [cited 2024 Feb 15]).
[c]Log2 fold change was calculated using the DESeq2 R package (Anders & Huber, 2010) and edge R Bioconductor package (Robinson et al., 2010) based on the raw read counts.

Table S10. Characteristics of down-regulated DEGs unique to the LVG-V versus HVG-V comparison (p<0.05, log2FC).

Gene symbol[a]	Gene ID[a]	Gene annotation[b]	Genomic accession[a]	Chromosome[a]	Start[a]	End[a]	log2FC[c]	p-value[c]
LOC113219341	113219341	small subunit ribosomal RNA	NW_020555813.1	LG9	2142	4064	-3.0	3.14365E-05
LOC100577331	100577331	cell wall integrity and stress response component 1	NC_037647.1	LG10	5365831	5367042.0	-2.3	0.00088488
LOC107964175	107964175	uncharacterized	NC_037640.1	LG3	2886606	2888333	-2.3	0.016359081
LOC107964569	107964569	uncharacterized	NC_037643.1	LG6	3026217	3031255	-2.2	0.00088488
LOC100578623	100578623	uncharacterized	NC_037647.1	LG10	1657374	1693623	-2.2	0.011347844
LOC102655028	102655028	uncharacterized	NC_037652.1	LG15	3555362	3559179	-2.1	0.047084104
LOC100576253	100576253	DNA ligase 1, transcript variant X2	NC_037643.1	LG6	11310906	11313905	-2.1	0.011347844
LOC102653737	102653737	uncharacterized	NC_037643.1	LG6	11310906	11313905	-2.0	0.048161753
LOC102655409	102655409	uncharacterized	NC_037641.1	LG4	12472825	12477931	-1.9	0.016359081
LOC102654882	102654882	uncharacterized	NC_037640.1	LG3	12339560	12340755	-1.9	0.042727289
LOC726509	726509	protein FRA10AC1	NC_037652.1	LG15	3708301	3710103	-1.6	0.004541664
LOC102655619	102655619	uncharacterized	NC_037653.1	LG16	4714255	4726576	-1.6	0.013179558
LOC102654781	102654781	protein G12	NC_037638.1	LG1	13302385	13304070	-1.5	0.003302796
LOC102653673	102653673	putative uncharacterized protein DDB_G0271606	NC_037646.1	LG9	8536421	8541685	-1.5	0.043504178
LOC102655506	102655506	uncharacterized	NC_037643.1	LG6	6860124	6865514	-1.2	0.028838137

[a]Gene symbol, gene ID, genomic accession, chromosome, start, and end based on the BeeBase gene identifiers of the Honey bee genome (Amel_HAv3.1; ftp://ftp.ncbi.nlm.nih.gov/genomes/Apis_mellifera: National Center for Biotechnology Information (Bethesda (MD) [24]: National Library of Medicine (US), National Center for Biotechnology Information; [1988] - [cited 2024 Feb 15]). Available from https://www.ncbi.nlm.nih.gov/
[b]Gene annotation based on g:profiler search for biological process terms, considering a depth of two hierarchical levels [30]. ShinyGO v.077 shearch (https://biosnips.org/category/next-generation-sequencing-analysis/ [32] - [cited 2024 Feb 15]).
[c]Log2 fold change was calculated using the DESeq2 R package (Anders & Huber, 2010) and edge R Bioconductor package (Robinson et al., 2010) based on the raw read counts.

Table S11. Characteristics of the down-regulated DEGs unique to the LVG-C versus LVG-C comparison (p<0.05, log2FC).

Gene symbol[a]	Gene ID[a]	Gene annotation[b]	Genomic accession[a]	Chromosome[a]	Start[a]	End[a]	log2FC[c]	p-value[c]
LOC100576126	100576126	uncharacterized	NC_037638.1	LG1	5713486	5721439	-2.466203382	3.53014E-06

[a]Gene symbol, gene ID, genomic accession, chromosome, start, and end site per chromosome was based on the Honey bee genome (Amel_HAv3.1; ftp://ftp.ncbi.nlm.nih.gov/genomes/Apis_mellifera: National Center for Biotechnology Information (Bethesda (MD) [24]: National Library of Medicine (US), National Center for Biotechnology Information; [1988] - [cited 2024 Feb 15]). Available from https://www.ncbi.nlm.nih.gov/

[b]Gene annotation based on g:profiler search for biological process terms, considering a depth of two hierarchical levels [30]. ShinyGO v.077 shearch (https://biosnips.org/category/next-generation-sequencing-analysis/ [32] - [cited 2024 Feb 15]).

[c]Log2 fold change was calculated using the DESeq2 R package (Anders & Huber, 2010) and edge R Bioconductor package (Robinson et al., 2010) based on the raw read counts.

Table S12. Characteristics of down-regulated DEGs shared between LVG-V versus HVG-V, LVG-V versus LVG-C and HVG-V versus HVG-C comparisons (p<0.05, log2FC).

Gene symbol[a]	Gene ID[a]	Gene annotation[b]	Genomic accession[a]	Chromosome[a]	Start[a]	End[a]	log2FC[c]	p-valu[c]
Mrjp3	406121	major royal jelly protein 3	NC_037648.1	LG11	2268450	2272030	-8.1	5.68582E-12

[a]Gene symbol, gene ID, genomic accession, chromosome, start, and end based on the BeeBase gene identifiers of the Honey bee genome (Amel_HAv3.1; ftp://ftp.ncbi.nlm.nih.gov/genomes/Apis_mellifera: National Center for Biotechnology Information (Bethesda (MD) [24]: National Library of Medicine (US), National Center for Biotechnology Information; [1988] - [cited 2024 Feb 15]). Available from https://www.ncbi.nlm.nih.gov/

[b]Gene annotation based on g:profiler search for biological process terms, considering a depth of two hierarchical levels [30]. ShinyGO v.077 shearch (https://biosnips.org/category/next-generation-sequencing-analysis/ [32] - [cited 2024 Feb 15]).

[c]Log2 fold change was calculated using the DESeq2 R package (Anders & Huber, 2010) and edge R Bioconductor package (Robinson et al., 2010) based on the raw read counts. was calculated using the DESeq2 R package (Anders & Huber, 2010) and edge R Bioconductor package (Robinson et al., 2010) based on the raw read counts.
